# δ2-Protocadherins organize parallel indirect basal ganglia circuits

**DOI:** 10.1126/sciadv.aec8746

**Published:** 2026-08-21

**Authors:** Naosuke Hoshina, Joshua M. Boeckers, Erin M. Johnson-Venkatesh, Miyuki Hoshina, Kana Matsumoto, Abhijnana Das, Veronica R. Rally, Jaanvi Sant, Akiko Terauchi, Shinji Kinoshita, Takafumi Inoue, Hisashi Umemori

**Affiliations:** ^1^ Department of Neurology, F.M. Kirby Neurobiology Center, Boston Children’s Hospital, Harvard Medical School, Boston, MA 02115, USA.; ^2^ Waseda Institute for Advanced Study, Waseda University, Tokyo 169-0051, Japan.; ^3^ Department of Life Science and Medical Bioscience, Faculty of Science and Engineering, Waseda University, Tokyo 162-8480, Japan.

## Abstract

The basal ganglia (BG) contain multiple parallel neural circuits, each of which may control different behaviors. However, how the distinct parallel BG circuits are molecularly organized is not known. Here, we show that two δ2-protocadherins (PCDHs), PCDH17 and PCDH10, which are homophilic cell adhesion molecules, establish and define two distinct indirect BG circuits that regulate different behaviors. PCDH17 and PCDH10 are expressed in a complementary expression pattern in the BG, anatomically defining two parallel indirect BG connections. Indirect pathway–specific *Pcdh17* and *Pcdh10* conditional knockout (cKO) mice show impaired establishment of the indirect BG circuits in a region-preferential manner. Last, the *Pcdh17* cKO mice show defects in task learning, while the *Pcdh10* cKO mice show defects in motor/sensory habituation. These results identify PCDH17 and PCDH10 as the molecular organizers for two distinct indirect BG circuits regulating different behaviors and reveal the molecular mechanisms for organizing parallel BG circuits.

## INTRODUCTION

The basal ganglia (BG) comprise a group of subcortical nuclei that include the striatum, external globus pallidus (GPe), internal globus pallidus (GPi), substantia nigra [SN; comprising pars reticulata (SNr) and pars compacta (SNc)], and subthalamic nucleus (STN). The striatum receives input from the cerebral cortex and projects to the GPi/SNr directly (direct pathway) or indirectly through the GPe and STN (indirect pathway). The GPi/SNr sends output back to the cortex via the thalamus, thereby constituting the cortico-BG-thalamo-cortical loop. Different cortical areas project to discrete regions of the BG in a topographic manner, constituting a parallel organization of functionally segregated circuits (*1*–*3*). Neurons in the ventromedial prefrontal and orbitofrontal cortices project to the ventral striatum; the dorsolateral prefrontal cortex to the anterior striatum; and the motor cortex to more caudal regions of the striatum, forming limbic, associative, and sensorimotor circuits, respectively (*4*, *5*). The limbic circuit appears to play a role in motivated behavior and empathic/socially appropriate behavior, the associative circuit is implicated in executive and cognitive function, and the sensorimotor circuit plays a role in sensorimotor modulation and movement regulation. While the anatomical distribution of parallel cortico-BG circuits has been described in mice (*6*), we do not know how these distinct circuits are organized molecularly; we do not know the molecular mechanisms by which these parallel BG circuits are established. Defects in the cortico-BG circuits are linked to many neurological and psychiatric disorders such as Parkinson’s disease, autism spectrum disorder (ASD), obsessive-compulsive disorder (OCD), schizophrenia, and depression (*1*, *7*). Thus, understanding how parallel cortico-BG circuits are organized should provide insight into potential strategies for treating the particular symptoms of each disorder.

Cell adhesion molecules play important roles in connecting appropriate neurons in the brain. For example, anatomically segregated parallel hippocampal circuits are organized on the basis of the expression of distinct cell adhesion molecules (*8*). Our previous work identified that two members of the δ2-protocadherin (PCDH) family (*9*), PCDH17 and PCDH10, are expressed in a complementary pattern in the BG (*10*), with PCDH17 and PCDH10 expression in regions implicated in associative and limbic circuits, respectively. Because δ2-PCDHs are homophilic cell adhesion molecules, we hypothesize that they serve as molecular organizers for the establishment of parallel BG circuits.

In this study, we focused on the striatal indirect pathway and examined the roles of PCDH17 and PCDH10 in the establishment of parallel BG circuits. PCDH17- and PCDH10-positive indirect spiny projection neurons (iSPNs) show complementary axonal projection patterns in the GPe that reflect parallel BG circuits. Indirect pathway–specific *Pcdh17* and *Pcdh10* conditional knockout (cKO) mice exhibit aberrant cell patterning and synapse development in the BG in a region-preferential manner. Behaviorally, the *Pcdh17* cKO mice show deficits in task learning, while the *Pcdh10* cKO mice show defects in locomotor and sensory habituation. These results show that parallel BG circuits are molecularly defined and that PCDH17 and PCDH10 organize functionally segregated, parallel indirect BG circuits that regulate distinct behaviors.

## RESULTS

### Parallel BG circuits defined by PCDH17 and PCDH10

**Fig. 1.**
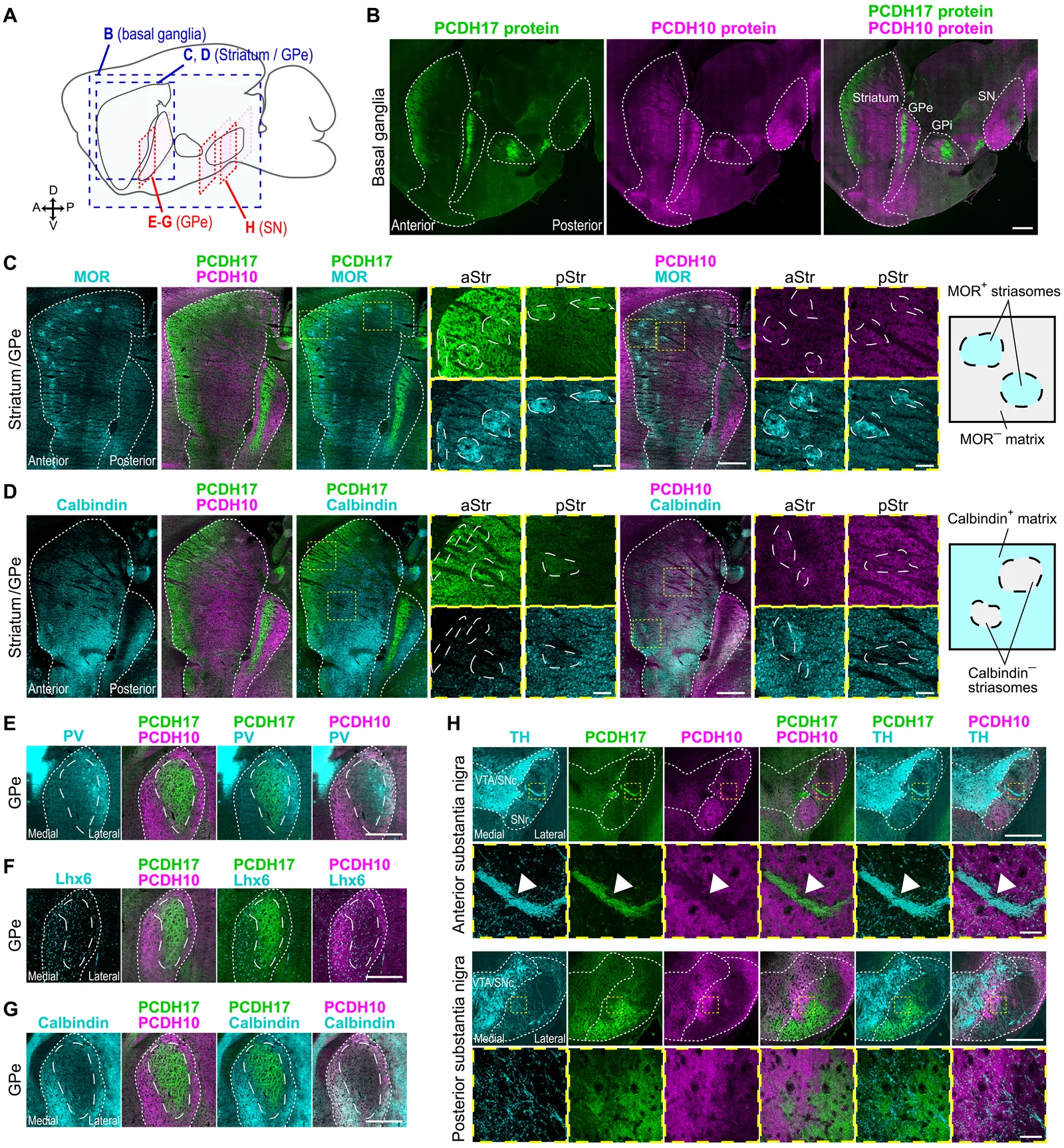
Expression patterns of PCDH17 and PCDH10 in BG circuits. (**A**) Illustration of sections shown in Fig. 1: blue frames: sagittal sections (B to D), red frames: coronal sections (E to H). (**B**) Double staining for PCDH17 (green) and PCDH10 (magenta) in a P8 sagittal BG section showing their complementary expression patterns within the BG nuclei. (**C** and **D**) Triple staining for PCDH17 (green), PCDH10 (magenta), and markers for striosome (MOR, C) or matrix (Calbindin, D) (cyan) in P9 to P12 sagittal striatum/GPe sections. Both PCDHs are enriched in striosomes (encircled by white dotted lines in high magnification images). They are also present in the matrix, particularly in regions where each PCDH is predominantly expressed [anterior striatum (aStr) for PCDH17 and posterior striatum (pStr) for PCDH10]. (**E** to **G**) Triple staining for PCDH17 (green), PCDH10 (magenta), and GPe subtype markers (cyan) in P7 to P11 coronal GPe sections. A long-dashed line marks the boundary between PCDH17^+^ inner and PCDH10^+^ outer GPe. While PV (lateral GPe, E) and Lhx6 (medial GPe, F) distribute across both PCDH domains, Calbindin (G) is prominent in the outer GPe and overlaps with the PCDH10 domain. (**H**) Triple staining for PCDH17 (green), PCDH10 (magenta), and TH (cyan) in P10 to P11 coronal SN sections. The boundary between SNc/VTA and SNr is indicated by a white dashed line. Magnified views (bottom panels) show that in the anterior SNr, PCDH17 concentrates within TH-positive “dendron bouquets” (arrowheads), while PCDH10 is excluded; in the posterior SNr, both PCDHs distribute across TH-negative and TH-positive regions. Scale bars, 0.5 mm (B, E, F, G, H, and low magnification C and D) and 0.1 mm (high magnification C and D). GPe, external globus pallidus; GPi, internal globus pallidus; SNc, substantia nigra pars compacta; SNr, substantia nigra pars reticulata; VTA, ventral tegmental area.

PCDH17 and PCDH10 are closely related PCDH members, which are transmembrane proteins with six extracellular cadherin domains. Both proteins mediate highly selective homophilic interactions, but not heterophilic interactions with each other (*10*). Building upon our previous anatomical characterization (*10*), we first examined their detailed expression patterns across the BG circuits. The double staining of BG sections for PCDH17 and PCDH10 proteins confirmed complementary expression patterns in the BG subregions, including the striatum, GPe, GPi, and SNr (Fig. 1, A and B, and fig. S1 and fig. S2). PCDH17 protein is mainly expressed in the anterior striatum, whereas PCDH10 protein is preferentially expressed in the posterior striatum, showing a complementary expression pattern along the anteroposterior axis. Their expression is also complementary in the GPe and GPi: PCDH17 protein is expressed in the inner regions of the globus pallidus (GP), whereas PCDH10 protein is expressed in the outer regions of the GP. In the SNr, PCDH17 and PCDH10 proteins are expressed in the posterior and anterior regions, respectively. We also performed in situ hybridization experiments to determine the mRNA expression of each *Pcdh* (fig. S3) (*10*). We found that the mRNA expression patterns of *Pcdh17* and *Pcdh10* are similar to the protein expression patterns of PCDH17 and PCDH10 in all subregions in the BG, where *Pcdh17* and *Pcdh10* show complementary expression patterns, with a small subset (10 to 17%) of overlap in each subregion.

To anchor this architecture within established molecular frameworks, we performed colabeling experiments with various BG markers (Fig. 1, C to H). In the striatum, costaining for μ-opioid receptor (MOR) (labeling the striosome) and Calbindin (labeling the matrix) (*11*) indicated that PCDH17 and PCDH10 are both abundant within the striosome, but they are also detected in the matrix, especially where each PCDH is abundant, i.e., PCDH17 in the anterior striatum and PCDH10 in the posterior striatum (Fig. 1, C and D). Thus, PCDH expression is not defined by the striosome/matrix structure. In the GPe, we costained for parvalbumin (PV) (labeling the lateral GPe) and LIM homeobox 6 (Lhx6) (labeling the medial GPe) (*12*). We found that both PCDH17- and PCDH10-positive regions contain PV-positive and Lhx6-positive cells, indicating that these markers do not coincide with PCDH-defined boundaries (Fig. 1, E and F). We also costained GPe sections for Calbindin, which is enriched in the outer GPe relative to the inner GPe (*13*). We found that Calbindin colocalizes with PCDH10 (outer GPe) but exhibits minimal overlap with PCDH17 (inner GPe) (Fig. 1G), suggesting that the inner-outer organization defined by these δ2-PCDHs aligns with calbindin expression patterns. In the SN, colabeling with tyrosine hydroxylase (TH) revealed that PCDH17 is localized at the SNc dendron bouquets in the anterior SNr (Fig. 1H, top); these bouquets represent the specialized terminal morphology of striosomal projections to dopaminergic neurons (*14*). In contrast, PCDH10 is excluded from the bouquets and is more diffusely distributed in the SNr, including both TH-positive and TH-negative regions. In the posterior SNr, both PCDHs are diffusely distributed in a complementary manner (Fig. 1H, bottom). Together, these results indicate that the PCDH-defined architecture does not simply match previously described molecular frameworks (*15*, *16*). The PCDH expression patterns do align with the Calbindin expression patterns in the GPe, but not with those in the striatum. Therefore, the PCDH-defined architecture in the BG represents an additional molecular dimension of BG circuit specialization (see Discussion).

### PCDH17 and PCDH10 define parallel iSPN axonal connections

**Fig. 2.**
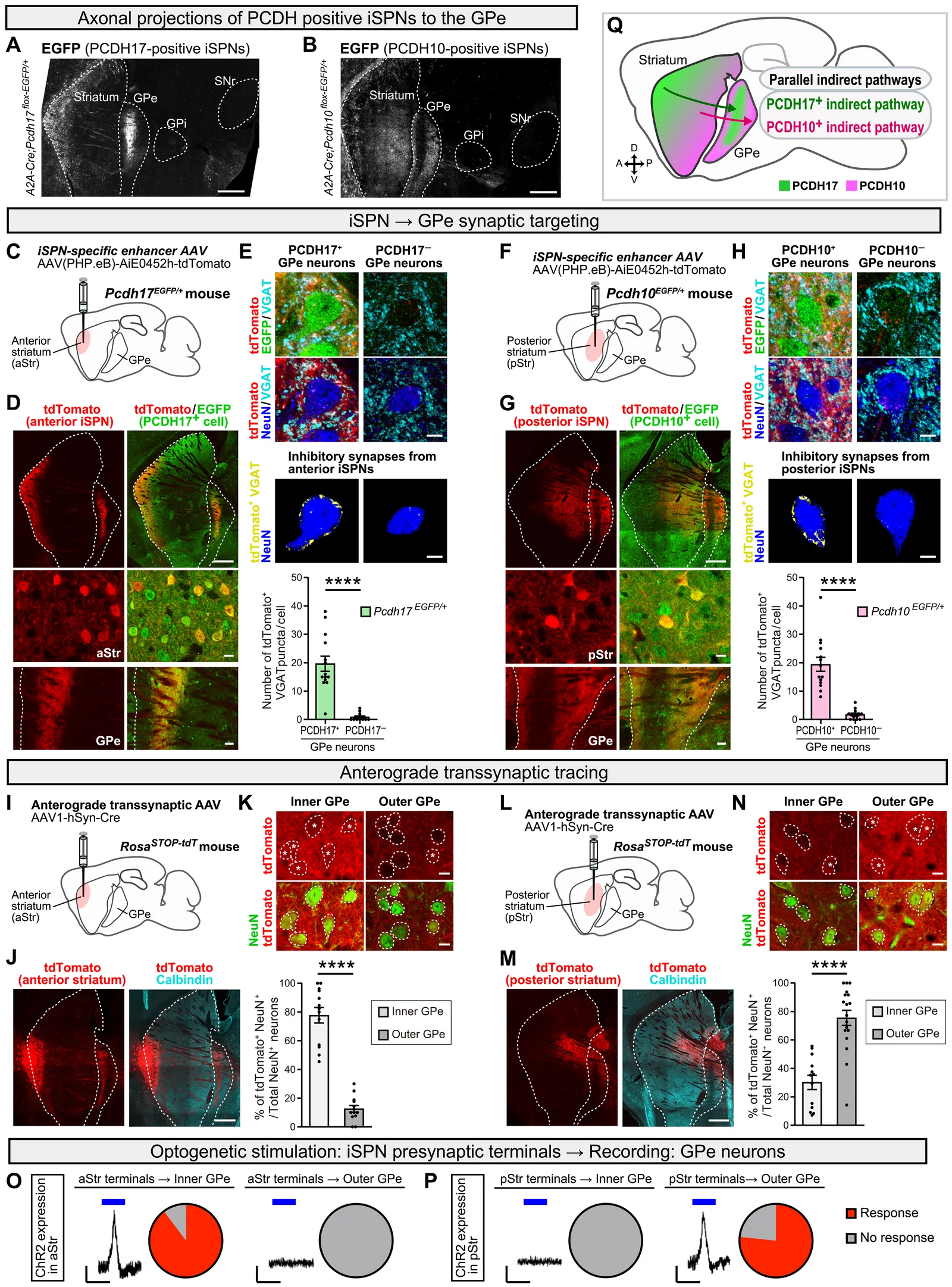
PCDH17 and PCDH10 define parallel iSPN connections. (**A** and **B**) Sagittal sections of P10-P12 *A2A-Cre;Pcdh^flox-EGFP/+^* mouse brains (EGFP in *Pcdh*-expressing iSPNs). PCDH17^+^ iSPNs are localized in aStr and project to inner GPe (A). PCDH10^+^ iSPNs are localized in pStr and project to outer GPe (B). (**C** to **H**) iSPN-specific tdTomato-expressing AAV was injected into aStr of *Pcdh17^EGFP/+^* (C) or pStr of *Pcdh10^EGFP/+^* (F) adult mice. aStr (D) or pStr (G) tdTomato-labeled iSPNs express EGFP, confirming that they are PCDH17^+^ or PCDH10^+^. Their axons specifically target inner (D) or outer (G) GPe. (E and H) iSPN inhibitory terminals (tdTomato^+^/VGAT^+^ yellow puncta) on GPe cell bodies (NeuN^+^; blue). Anterior iSPNs innervate PCDH17^+^ GPe neurons (E). Posterior iSPNs innervate PCDH10^+^ GPe neurons (H). *n* = 14 cells from two to three mice per group. (**I** to **N**) Transsynaptic AAV1-hSyn-Cre was injected into aStr (I) or pStr (L) of adult *Rosa^STOP-tdT^* mice. tdTomato-labeled aStr axons target Calbindin-negative inner GPe (J). tdTomato-labeled pStr axons target Calbindin-positive outer GPe (M). (K and N) Quantification of tdTomato^+^ (i.e., Cre^+^)/NeuN^+^ GPe neurons (asterisks). Anterior iSPNs synapse to inner GPe neurons (K). Posterior iSPNs synapse to outer GPe neurons (N). *n* = 13 to 18 fields from four to five mice per group. (**O** and **P**) ChR2 was expressed in adult aStr (O) or pStr (P). Light stimulation (blue lines) of aStr iSPN terminals evokes IPSCs in inner GPe neurons, while the stimulation of pStr iSPN terminals evokes IPSCs in outer GPe neurons. *n* = 7 to 30 neurons from five to eight mice per group. (**Q**) Schematic of PCDH17/PCDH10 defined parallel connections. Scale bars, 0.5 mm (A, B, J, M, and low magnification D and G), 50 μm (GPe in D and G), 10 μm (K, N, and aStr/pStr in D and G), 5 μm (E and H), and 10 pA/5 ms (O and P). ****P < 0.0001 by Student’s *t* test.

Since δ2-PCDHs are homophilic cell adhesion molecules, we next wanted to know whether BG subregions expressing each PCDH are preferentially connected with each other. Axons from iSPNs in the striatum project to the GPe. To test the idea that the striatal regions expressing PCDH17 and PCDH10 are preferentially connected to the GPe regions expressing PCDH17 and PCDH10, respectively, we generated conditional enhanced green fluorescent protein (EGFP) knock-in mice (fig. S4, A and B). For this, we generated an allele (“flox-EGFP”) in which the first exon of the *Pcdh* gene is flanked by loxP sequences, followed by the EGFP expression cassette. EGFP is designed to express under the *Pcdh* promoter, after the first exon of the *Pcdh* gene is removed by Cre recombinase. We mated *Pcdh^flox-EGFP/+^* mice with *A2A-Cre* mice (*17*, *18*), in which Cre is selectively expressed by iSPNs in the striatum, to label *Pcdh*-expressing iSPNs with EGFP. The EGFP staining of BG sections from *A2A-Cre;Pcdh17^flox-EGFP/+^* mice showed that *Pcdh17*-expressing iSPNs are distributed in the anterior striatum and their axon terminals project to the inner GPe (Fig. 2A), where *Pcdh17* is highly expressed (Fig. 1B and fig. S3). In *A2A-Cre;Pcdh10^flox-EGFP/+^* mice, *Pcdh10*-expressing iSPNs are distributed in the posterior striatum, and their axon terminals project to the outer GPe (Fig. 2B), where *Pcdh10* is highly expressed (Fig. 1B and fig. S3). EGFP signals were not detected in the GPi and SNr (Fig. 2, A and B), the targets of the direct spiny projection neurons (dSPNs).

We further examined whether PCDH-expressing iSPNs preferentially form synaptic contacts with GPe neurons expressing the same PCDH. For this, we used *Pcdh17^EGFP/+^* and *Pcdh10^EGFP/+^* knock-in mice, in which EGFP is constitutively expressed under the control of the endogenous *Pcdh* promoter due to germline recombination (fig. S4). In these mice, EGFP labels both PCDH-expressing neurons and their axonal terminals. Inhibitory presynaptic terminals in the GPe, which mainly originate from iSPNs, were identified by immunostaining for vesicular GABA transporter (VGAT) (inhibitory presynaptic marker). PCDH-positive inhibitory terminals were identified as EGFP^+^/VGAT^+^ terminals. We then quantified the number of PCDH-positive inhibitory terminals formed onto PCDH-positive and PCDH-negative GPe neurons. We found that in *Pcdh17^EGFP/+^* mice, PCDH17-positive inhibitory terminals selectively innervated PCDH17-positive GPe neurons, with little innervation on PCDH17-negative neurons (fig. S5, A and B). A similar pattern was observed in *Pcdh10^EGFP/+^* mice, where PCDH10-positive inhibitory terminals preferentially target PCDH10-positive GPe neurons compared to PCDH10-negative GPe neurons (fig. S5, C and D). To confirm that these contacts represent bona fide synapses with properly apposed pre- and postsynaptic structures, we further stained the sections for the inhibitory postsynaptic marker Gephyrin. The quantification of EGFP^+^/VGAT^+^ presynaptic puncta that were apposed to EGFP^+^/Gephyrin^+^ postsynaptic puncta revealed that PCDH17-positive and PCDH10-positive inhibitory synapses selectively form on their respective PCDH-positive GPe neurons (fig. S5, E and F).

Inhibitory synapses in the GPe contain inputs from iSPNs from the striatum and recurrent inhibitory inputs from the GPe. To isolate iSPN-derived projections, we utilized an iSPN-specific enhancer-driven adeno-associated virus (AAV), AAV-AiE0452h-tdTomato (*19*). Injections of the AAV into the anterior striatum of *Pcdh17^EGFP/+^* mice confirmed that the labeled iSPNs were PCDH17-positive and targeted the inner GPe (Fig. 2, C and D). Conversely, injections into the posterior striatum of *Pcdh10^EGFP/+^* mice labeled PCDH10-positive iSPNs specifically targeting the outer GPe (Fig. 2, F and G). The quantification of tdTomato^+^/VGAT^+^ terminals on PCDH-positive and PCDH-negative GPe neurons confirmed that anterior iSPN terminals selectively innervate PCDH17-positive GPe neurons (Fig. 2E), while posterior iSPN terminals preferentially innervate PCDH10-positive GPe neurons (Fig. 2H).

To show that these anatomical innervations represent functional synaptic connections, we first employed an anterograde transsynaptic tracing strategy using AAV serotype 1 expressing Cre (AAV1-hSyn-Cre). In this system, Cre is effectively transported from presynaptic terminals to postsynaptic targets (*20*). We injected AAV1-hSyn-Cre into the striatum of *Rosa^STOP-tdT^* reporter mice, which express tdTomato only in the presence of Cre recombinase, so that tdTomato expression is triggered in GPe neurons that receive direct, functional synaptic input from the infected striatal neurons. tdTomato signals from the anterior striatum were largely confined to the Calbindin-negative inner GPe (Fig. 2, I and J), while tdTomato signals from the posterior striatum were primarily distributed in the Calbindin-positive outer GPe (Fig. 2, L and M). The quantification of transsynaptically labeled tdTomato^+^ GPe neurons confirmed that anterior and posterior striatal neurons are functionally connected to inner and outer GPe neurons, respectively (Fig. 2, K and N). To provide physiological evidence for these parallel connections, we next performed optogenetic electrophysiology. The optogenetic stimulation of Channelrhodopsin-2 (ChR2)–expressing terminals originating from the anterior striatum evoked inhibitory postsynaptic currents (IPSCs) selectively in the inner GPe neurons (Fig. 2O). Conversely, the stimulation of terminals from the posterior striatum selectively elicited IPSC responses in the outer GPe neurons (Fig. 2P).

Collectively, these results provide direct anatomical and functional evidence that PCDH17 and PCDH10 define molecularly segregated, parallel indirect pathways through biased connectivity between iSPNs and GPe neurons expressing the matching PCDH (Fig. 2Q).

### Generation of iSPN-specific *Pcdh17* and *Pcdh10* cKO mice

To investigate the roles of PCDH17 and PCDH10 in iSPNs, we generated iSPN-specific *Pcdh* cKO mice (fig. S4). For this, we first generated the *Pcdh*-*flox* allele by removing the FRT-flanked EGFP cassette through mating with *ACTB-FLPe* mice. These mice were then crossed with the *A2A-Cre* mice to generate iSPN-specific cKO via the deletion of the first exon of the *Pcdh* gene. The levels of PCDH17 and PCDH10 proteins were reduced in the striatum and GPe (the target of iSPNs), but not in the SNr (a target of dSPNs), in the cKO mice (fig. S6, A and B). The protein remaining in the striatum would be from dSPNs and input neurons to the striatum. The protein remaining in the GPe would be from GPe neurons (see fig. S3). Furthermore, we confirmed that PCDH17 and PCDH10 protein levels were preserved in major input regions to the striatum—including the cerebral cortex, hippocampus, and thalamus—in the cKO mice (fig. S6, C and D).

We further examined whether PCDH17 and PCDH10 were selectively deleted in iSPNs and not in dSPNs in the striatum. Because dopamine receptors D1 (D1R) and D2 (D2R) are selectively expressed by dSPNs and iSPNs, respectively (*13*), we performed triple staining for PCDH, D1R, and D2R. In control mice, PCDH17 and PCDH10 signals were detected in the anterior and posterior striatum, respectively, in both iSPNs and dSPNs. In the anterior striatum of *Pcdh17* cKO mice, the PCDH17 signal was diminished in iSPNs but not in dSPNs (fig. S7A). In the posterior striatum of *Pcdh10* cKO, the PCDH10 signal in iSPNs was diminished, while there was no change in dSPNs (fig. S7B). The remaining signals in iSPNs in cKO mice are likely due to technical constraints in defining precise cellular boundaries during quantification. These results indicate that in both *Pcdh17* cKO and *Pcdh10* cKO mice, PCDH17 and PCDH10 are specifically inactivated in iSPNs, but not in dSPNs.

To exclude the possibility that the expression patterns of D1R and D2R are altered in *Pcdh* cKO mice, we labeled iSPNs with tdTomato by crossing *A2A-Cre* mice with *Rosa^STOP-tdT^* mice. The *A2A-Cre* mouse expresses Cre in iSPNs independent of D2R expression (*17*, *18*), allowing us to identify iSPNs as tdTomato^+^ cells. Using this strategy, we found that in *Pcdh17* control (tdT), *Pcdh17* cKO (tdT), *Pcdh10* control (tdT), and *Pcdh10* cKO (tdT) mice, tdTomato^+^ cells are D2R positive (fig. S7, C and D), indicating that the expression pattern of dopamine receptors is not changed in *Pcdh* cKO mice.

Last, to determine whether the loss of one PCDH protein affects the expression pattern of the other, we analyzed PCDH10 protein expression in iSPN-*Pcdh17* cKO mice and PCDH17 protein expression in iSPN-*Pcdh10* cKO mice. In *Pcdh17* cKO mice, the PCDH10 expression pattern and levels were not changed in the anterior striatum and inner GPe, where PCDH17 levels were decreased (fig. S6E). Similarly, in *Pcdh10* cKO mice, the PCDH17 expression pattern and levels were not changed in the posterior striatum and outer GPe, where PCDH10 levels were decreased (fig. S6F). These findings suggest that the loss of PCDH17 does not affect PCDH10 expression and vice versa.

### Region-specific abnormal SPN aggregations in iSPN-*Pcdh* cKO mice

The *iSPN-Pcdh17* cKO and *iSPN-Pcdh10* cKO mice did not die early, and the mice were fertile. In addition, the gross appearance of the brains of both cKO mice was normal. Using the iSPN-*Pcdh* cKO mice, we investigated the SPN distribution in the striatum, axonal targeting by iSPNs, SPN dendrite morphology, synapses formed by iSPNs, synapses formed onto iSPNs, synaptic physiology, and the behavioral consequences of *Pcdh* deletion.

**Fig. 3.**
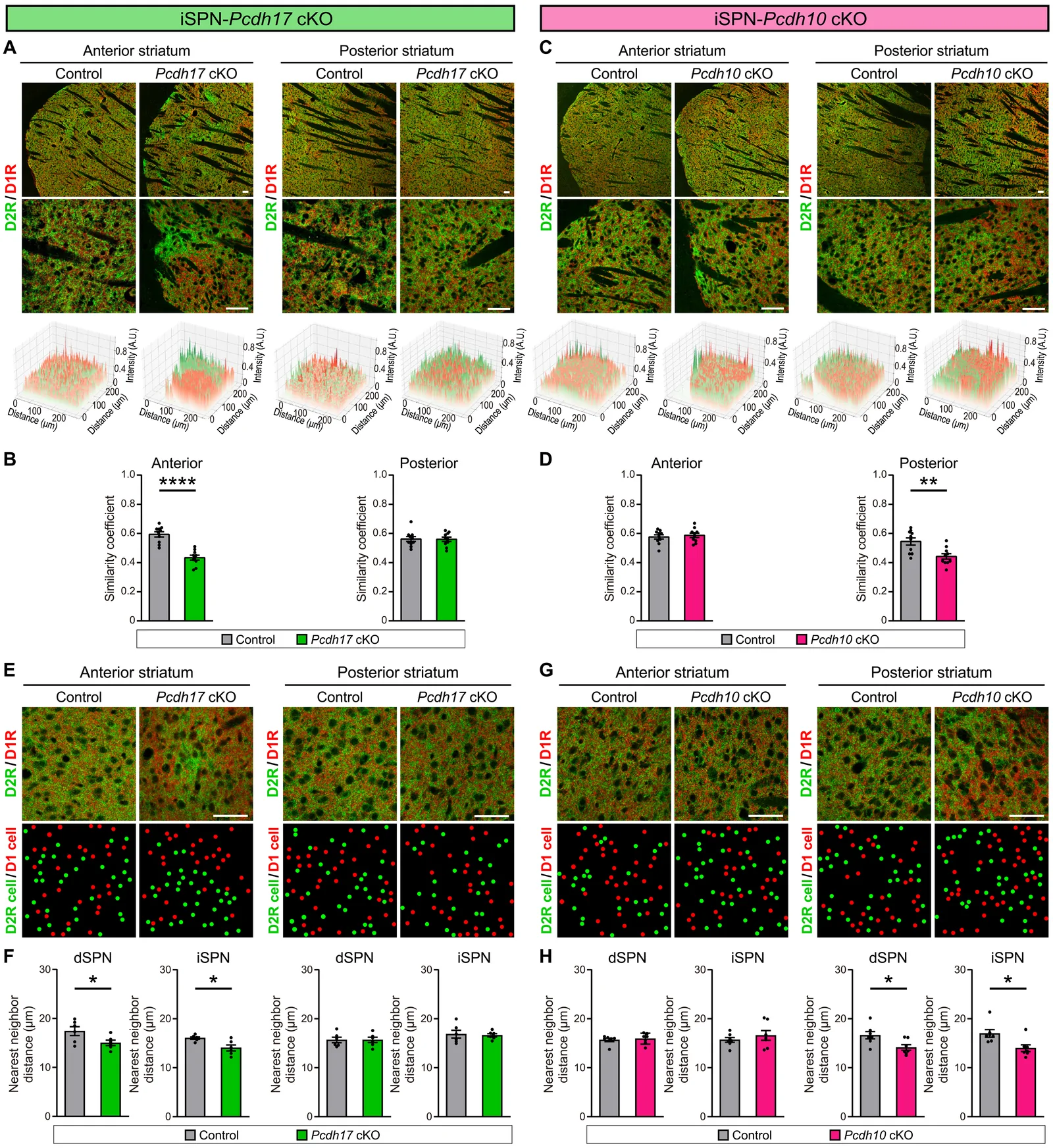
Abnormal aggregation of SPNs in the striatum of iSPN-*Pcdh* cKO mice. (**A** to **D**) Region-specific abnormal D1R and D2R distribution in iSPN-*Pcdh* cKO mice. Double staining for D2R (green) and D1R (red) in anterior and posterior striatal sections from 3- to 4-week-old *Pcdh17* cKO and Control (A) and *Pcdh10* cKO and control (C). Top panels: Low-magnification images; bottom panels: high-magnification images. Contour maps for D2R (green) and D1R (red) are shown below. (B and D) The similarity coefficient in the anterior (*P* < 0.0001) and posterior (*P* = 0.9303) striatum for *Pcdh17* cKO and control mice (B), and in the anterior (*P* = 0.6093) and posterior (*P* = 0.0037) striatum for *Pcdh10* cKO and control mice (D). *n* = 10 fields from two mice per genotype. (**E** to **H**) Region-specific abnormal cell body spacing of dSPNs (D1R positive) and iSPNs (D2R positive) in iSPN-*Pcdh* cKO mice. (E and G) Top: Double staining for D1R and D2R of anterior and posterior striatal sections from 3-week-old *Pcdh17* cKO and control (E) and *Pcdh10* cKO and control (G). Bottom: The positions of dSPN and iSPN cell bodies were extracted and marked with red and green circles, respectively. (F and H) Quantification of the nearest neighbor distance of SPN cell bodies in the anterior (dSPN; *P* = 0.0480, iSPN; *P* = 0.0123) and posterior striatum (dSPN; *P* = 0.9916, iSPN; *P* = 0.7961) for *Pcdh17* cKO and control mice and in the anterior (dSPN; *P* = 0.6388, iSPN; *P* = 0.4247) and posterior striatum (dSPN; *P* = 0.0216, iSPN; *P* = 0.0135) for *Pcdh10* cKO and Control mice. *n* = 6 to 7 fields from three to four mice per genotype. Scale bars, 50 μm. *P < 0.05, **P < 0.01, and ****P < 0.0001 by Student’s *t* test.

We first asked whether the iSPN-specific loss of PCDHs alters the spatial organization of the striatal neurons. We examined the distribution of dSPNs and iSPNs in the striatum of iSPN-*Pcdh* cKO mice by staining for D1R and D2R at 3 to 4 weeks of age. In the normal striatum at this age, SPNs expressing D1R and D2R are evenly distributed (*21*). In *Pcdh17* cKO mice, we found that D1R and D2R signals showed abnormal aggregations in the anterior striatum, where PCDH17 is highly expressed (Fig. 3, A and B). D1R and D2R signals were evenly distributed in the posterior striatum of *Pcdh17* cKO mice. In contrast, in *Pcdh10* cKO mice, D1R and D2R signals showed abnormal aggregations only in the posterior striatum, where PCDH10 is highly expressed (Fig. 3, C and D).

The changes in D1R/D2R signals could be due to changes in SPN distribution and/or changes in SPN morphology. To further assess SPN distribution, we identified the cell bodies of dSPNs and iSPNs based on D1R and D2R expression (Fig. 3, E and G, and see also fig. S7) (*22*). We then performed the nearest neighbor analysis to quantify the clustering of dSPNs and iSPNs in *Pcdh17* cKO and *Pcdh10* cKO mice. We found that in the anterior striatum, but not the posterior striatum, of *Pcdh17* cKO mice, the nearest neighbor distances for dSPNs and iSPNs were reduced relative to control mice (Fig. 3, E and F). On the other hand, in the posterior striatum, but not the anterior striatum, of *Pcdh10* cKO mice, the nearest neighbor distances for dSPNs and iSPNs were reduced relative to control mice (Fig. 3, G and H). These results indicate that in regions where each PCDH is abundant, PCDH-positive dSPNs and PCDH-negative iSPNs were segregated and abnormally clustered, suggesting that PCDH17 and PCDH10 play important roles in evenly distributing dSPNs and iSPNs in a region-specific manner.

To assess SPN morphology, we evaluated cell body size and dendritic morphology by sparsely labeling SPNs. For this, we retro-orbitally injected AAVs expressing membrane-tagged EGFP into *Pcdh17* cKO (tdT) and *Pcdh10* cKO (tdT) mice, in which iSPNs are labeled with tdTomato. tdTomato^+^/mEGFP^+^ striatal cells were identified as iSPNs, while tdTomato^−^/mEGFP^+^ striatal cells were identified as dSPNs. We found that the size of cell bodies and the complexity of dendrites, assessed by the number of dendritic crossings, were normal in both the anterior and posterior striatum of *Pcdh17* cKO (tdT) and *Pcdh10* cKO (tdT) mice (fig. S9). These findings suggest that the loss of PCDH17 or PCDH10 in iSPNs does not affect the morphology of either dSPNs or iSPNs, supporting the notion that the changes in D1R/D2R signals (Fig. 3, A to D) reflect the redistribution of SPNs rather than morphological changes of SPNs.

To determine when abnormal SPN aggregations occur during development, we examined the distribution of dSPNs and iSPNs at an earlier time point, postnatal day 0 (P0). We found no differences in the distribution patterns of dSPNs and iSPNs at P0 in either the anterior or posterior striatum of *Pcdh17* cKO and *Pcdh10* cKO mice (fig. S8). Thus, while the distribution patterns of dSPNs and iSPNs are similar immediately after birth, differences emerge by 3 to 4 weeks of age. The striatum contains two compartments termed the patch and the matrix. In wild-type mice at P0, both dSPNs and iSPNs are highly enriched in the patch structure of the striatum. By P28, they are distributed into both the patch and matrix structures (*23*). Therefore, abnormal aggregations occur when SPNs distribute to the patch and matrix structures between P0 and P28 in iSPN-*Pcdh* cKO mice.

### Normal iSPN axonal targeting in iSPN-*Pcdh* cKO mice

We next asked whether PCDH17 and PCDH10 regulate the axonal projections of iSPNs. To visualize *Pcdh*-expressing iSPN axons, we used *A2A-Cre;Rosa^STOP-tdT^;Pcdh^flox-EGFP^* mice, in which all iSPNs express tdTomato, and *Pcdh*-expressing iSPNs express EGFP (fig. S10). We compared iSPN axonal projections between adult *A2A-Cre; Rosa^STOP-tdT^; Pcdh^flox-EGFP/+^* (Het) and *A2A-Cre;Rosa^STOP-tdT^;Pcdh^flox-EGFP/flox^* (cKO) mice. We assessed the targeting of PCDH17- and PCDH10-expressing iSPN axons in the GPe by evaluating the EGFP-positive regions in the GPe (fig. S10, H, I, K, and L; see fig. S10, A to G for the evaluation methods). We did not find differences in the EGFP expression patterns between Het and cKO mice. The density of iSPN axon terminals in the GPe, assessed by quantifying the EGFP intensity in the GPe (normalized to that in the striatum), was also similar between Het and cKO mice for both PCDHs (fig. S10, J and M). These results suggest that the targeting and density of iSPN axon terminals in the GPe are normal in the absence of PCDH17 or PCDH10.

### Impaired inhibitory synapses in the GPe in iSPN-*Pcdh* cKO mice

**Fig. 4.**
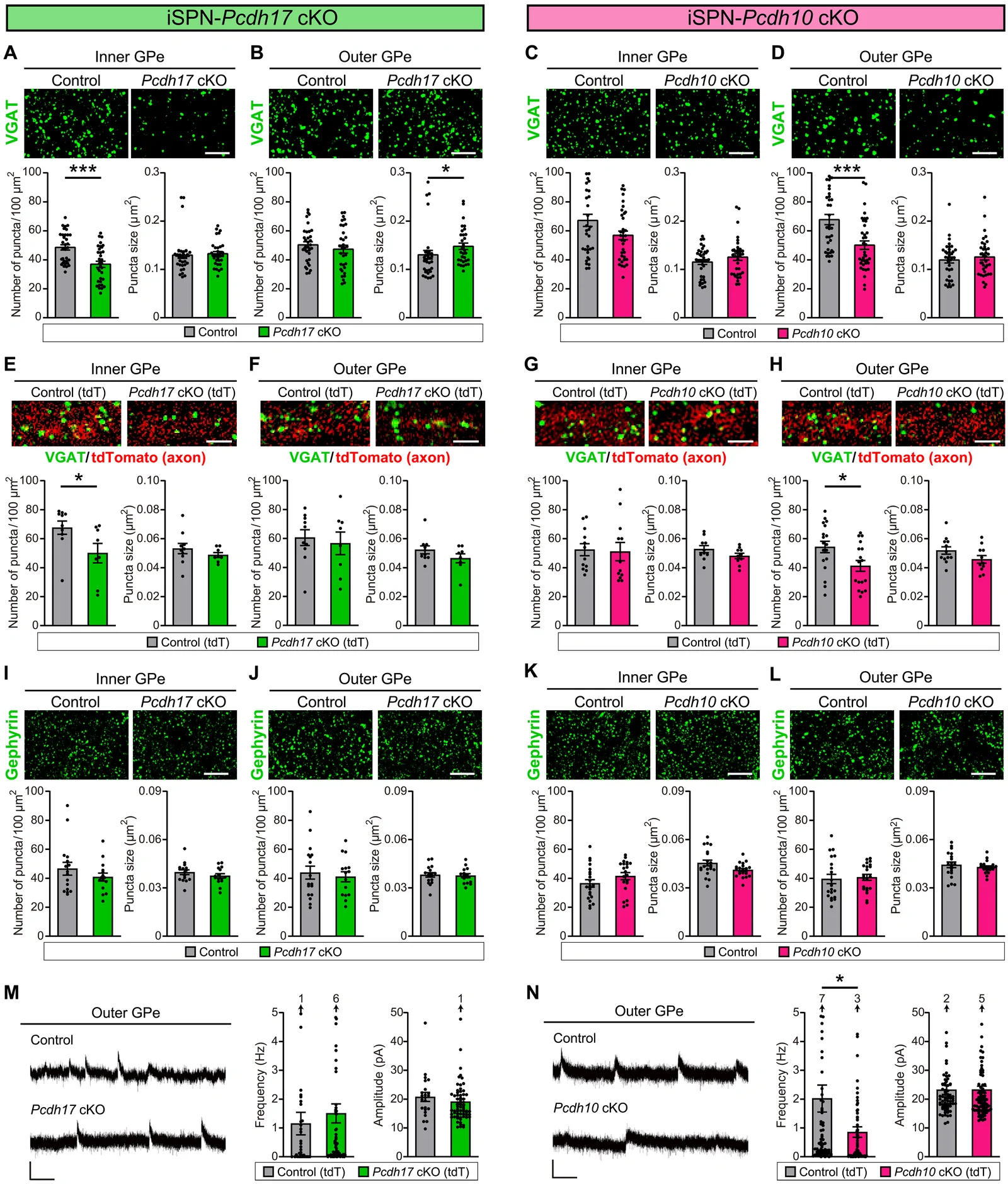
Region-specific inhibitory synapse impairment in the GPe of *Pcdh* cKO mice. (**A** to **L**) VGAT (A to D), VGAT in tdTomato-labeled iSPN axons (E to H), and Gephyrin (I to L) puncta in the inner GPe (iGPe) and outer GPe (oGPe) of 2-week-old mice. In *Pcdh17* cKO, VGAT density is decreased in the iGPe (A, *P* = 0.0003) but not the oGPe (B, *P* = 0.3729). VGAT size is increased in the oGPe (B, *P* = 0.0101) but not the iGPe (A, *P* = 0.2068). In iSPN axons, VGAT density is reduced in the iGPe (E, *P* = 0.0250) but not the oGPe (F, *P* = 0.6498). Gephyrin density (I, *P* = 0.4519; J, *P* = 0.8158) and size (I, *P* = 0.4402; J, *P* = 0.6479) remain unchanged. In *Pcdh10* cKO, VGAT density is decreased in the oGPe (D, *P* = 0.0002) but not the iGPe (C, *P* = 0.0880). VGAT size remains unchanged (C, *P* = 0.3075; D, *P* = 0.5850). In iSPN axons, VGAT density is decreased in the oGPe (H, *P* = 0.0386) but not the iGPe (G, *P* = 0.4172). Gephyrin density (K, *P* = 0.1339; L, *P* = 0.5396) and size (K, *P* = 0.0687; L, *P* = 0.4038) remain unchanged. (**M** and **N**) sIPSCs from oGPe neurons at P12 to P18. (M) In *Pcdh17* cKO, frequency (*P* = 0.5245) and amplitude (*P* = 0.3836) are unchanged. (N) In *Pcdh10* cKO, frequency is decreased (*P* = 0.0128), while amplitude is unchanged (*P* = 0.9701). *n* = 8 to 37 fields from 4 to 10 mice per genotype (imaging); *n* = 23 to 99 neurons from 4 to 12 mice per group (electrophysiology). Scale bars, 2 μm (imaging) and 20 pA/100 ms (electrophysiology). *P < 0.05 and ***P < 0.001 by Mann-Whitney *U* test (imaging) and Student’s *t* test (electrophysiology).

Our previous studies showed that PCDH17 and PCDH10 are localized at both excitatory and inhibitory synapses (*10*, *24*). Therefore, we tested whether synapse development is impaired in iSPN-*Pcdh* cKO mice. We first investigated synapses formed by the iSPNs, i.e., inhibitory synapses in the GPe. For this, we quantified the densities and sizes of VGAT and Gephyrin puncta in the inner GPe, the target of PCDH17-expressing iSPNs, and the outer GPe, the target of PCDH10-expressing iSPNs (see Fig. 2Q for the circuit scheme). In *Pcdh17* cKO mice, the density of VGAT was significantly decreased in the inner GPe [Fig. 4A; *P* = 0.0003; mean difference (MD) = −11.7], but not in the outer GPe (Fig. 4B). The size of VGAT puncta was increased in the outer GPe (Fig. 4B). In contrast, in *Pcdh10* cKO mice, the density of VGAT was significantly decreased in the outer GPe (Fig. 4D; *P* = 0.0002; MD = −17.7), but not in the inner GPe (Fig. 4C). The size of VGAT puncta was similar between *Pcdh10* cKO and control mice (Fig. 4, C and D). To compare the puncta density of VGAT between the inner GPe and outer GPe, we normalized the puncta density in *Pcdh* cKO mice to that in relevant controls and compared the normalized density between the two regions. We found a significant decrease in VGAT density in the inner GPe relative to outer GPe in *Pcdh17* cKO mice (fig. S11A; *P* = 0.0178). A modest reduction was also observed in the outer GPe compared to the inner GPe in *Pcdh10* cKO mice (fig. S11B; *P* = 0.0764). To specifically analyze VGAT signals in iSPN axons, we labeled iSPN axons with tdTomato by crossing *Pcdh* cKO mice with *Rosa^STOP-tdT^* mice (tdT). In *Pcdh17* cKO (tdT) mice, VGAT density was decreased in iSPN axons in the inner GPe (Fig. 4E; *P* = 0.0250), but not in the outer GPe (Fig. 4F). In contrast, in *Pcdh10* cKO (tdT) mice, VGAT density was decreased in iSPN axons in the outer GPe (Fig. 4H; *P* = 0.0386), but not in the inner GPe (Fig. 4G). The size of VGAT puncta in iSPN axons did not differ between *Pcdh* cKO (tdT) and control (tdT) mice (Fig. 4, E to H). The density and size of Gephyrin puncta were similar between *Pcdh* cKO and control mice (Fig. 4, I to L).

To functionally assess inhibitory synapses, we performed electrophysiological recordings of spontaneous iIPSCs (sIPSCs) from neurons in the outer GPe. In *Pcdh17* cKO (tdT) mice, both the amplitude and frequency of sIPSCs were comparable to control (tdT) mice (Fig. 4M). However, in *Pcdh10* cKO (tdT) mice, the frequency of sIPSCs was significantly reduced in the outer GPe compared to control (tdT) mice, while the amplitude remained unchanged (Fig. 4N). In the outer GPe of *Pcdh10* cKO (tdT) mice, the decrease in frequency, which suggests an impaired inhibitory presynaptic function, alongside preserved amplitude, which suggests normal inhibitory postsynaptic strength, is consistent with our findings that VGAT was reduced while Gephyrin remained unchanged. Together, these results indicate that in *Pcdh17* cKO and *Pcdh10* cKO mice, inhibitory presynaptic development is preferentially impaired in the region of the GPe targeted by PCDH17- or PCDH10-expressing iSPNs.

We next examined whether the synaptic deficits persisted into adulthood. For this, we examined the synaptic deficits in 2-month-old mice (at the age they are used for behavioral tests; see below). The density and size of VGAT puncta were significantly decreased in the inner GPe of *Pcdh17* cKO mice, while a selective reduction in VGAT density was observed in the outer GPe of *Pcdh10* cKO mice (fig. S12). These results indicate that region-specific synaptic impairments persist into adulthood and that the impairments are not a transient delay.

We then examined whether these PCDHs can induce inhibitory presynaptic differentiation. For this, we used a heterologous coculture system. Human embryonic kidney (HEK) 293T cells overexpressing either EGFP, PCDH17-EGFP, or PCDH10-EGFP were cocultured with striatal neurons for 24 hours and stained for VGAT. We found that there were no significant differences in the number and size of VGAT puncta on HEK cells between the three conditions (fig. S13). These results suggest that while PCDH17 and PCDH10 are essential for iSPN-GPe synapses in vivo, they do not induce synapses, but rather that they may contribute to the stabilization of synapses (see Discussion).

### Impaired synapses formed onto iSPNs in the striatum of iSPN-*Pcdh* cKO mice

**Fig. 5.**
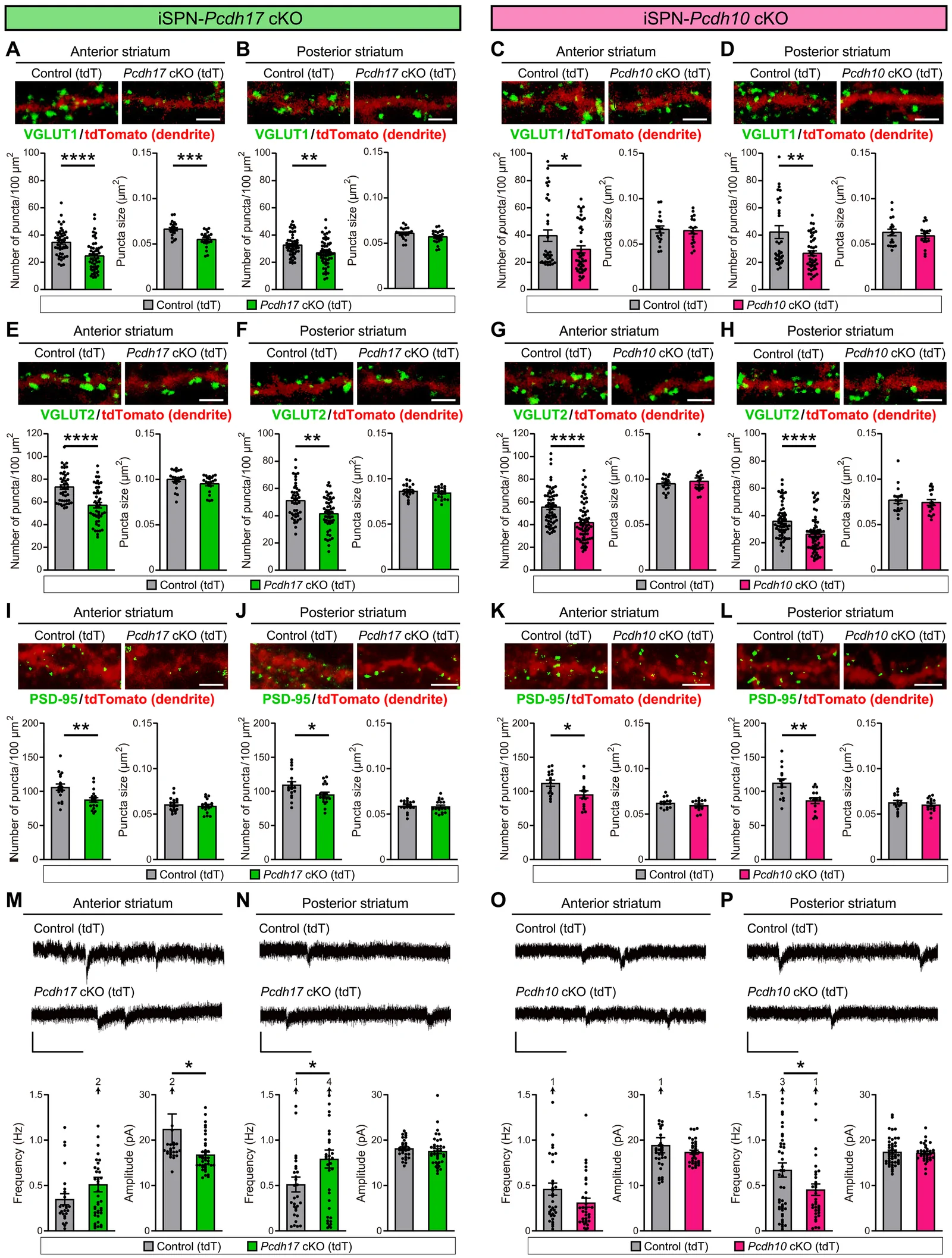
Reduced excitatory synapses formed on iSPNs in *Pcdh* cKO mice. (**A** to **L**) VGLUT1 (A to D), VGLUT2 (E to H), and PSD-95 (I to L) puncta on tdTomato-labeled iSPN dendrites in aStr and pStr of 2-week-old mice. In *Pcdh17* cKO aStr and pStr, the densities of VGLUT1 (A, *P* < 0.0001; B, *P* = 0.0023), VGLUT2 (E, *P* < 0.0001; F, *P* = 0.0014), and PSD-95 (I, *P* = 0.0037; J, *P* = 0.0290) are decreased. VGLUT1 size is only reduced in aStr (A, *P* = 0.0001; B, *P* = 0.1024). VGLUT2 and PSD-95 sizes remain unchanged [*P* = 0.1212 (E), 0.4573 (F), 0.6337 (I), 0.4570 (J)]. In *Pcdh10* cKO aStr and pStr, the densities of VGLUT1 (C, *P* < 0.0001; D, *P* = 0.0080), VGLUT2 (G, *P* < 0.0001; H, *P* < 0.0001) and PSD-95 (K, *P* = 0.0367; L, *P* = 0.0043) are decreased. Puncta sizes are unchanged [*P* = 0.9378 (C), 0.6960 (D), 0.9378 (G), 0.6837 (H), 0.5598 (K), and 0.5125 (L)]. (**M** to **P**) sEPSCs from iSPNs in aStr and pStr at P12 to P18. In *Pcdh17* cKO, sEPSC amplitude was decreased in aStr (M, *P* = 0.0431; N, *P* = 0.4118); frequency was increased in pStr (M, *P* = 0.1521; N, *P* = 0.0372). In *Pcdh10* cKO, sEPSC frequency was decreased in pStr (O, *P* = 0.0792; P, *P* = 0.0422); amplitude was unchanged (O, *P* = 0.3864; P, *P* = 0.3644). *n* = 15 to 68 fields from 5 to 10 mice per genotype (A to L) and 25 to 46 neurons from 5 to 8 mice per genotype (M to P). Scale bars, 2 μm (A to L) and 20 pA/100 ms (M to P). *P < 0.05, **P < 0.01, ***P < 0.001, and ****P < 0.0001 by Mann-Whitney *U* test (A to H) and Student’s *t* test (M to P).

We next investigated excitatory synapses formed onto iSPNs in the striatum of iSPN-*Pcdh* cKO mice. Striatal iSPNs receive vesicular glutamate transporter 1 (VGLUT1)–positive excitatory synapses from the cerebral cortex and VGLUT2-positive excitatory synapses from the thalamus (*25*). We have previously shown that PCDH17 and PCDH10 show complementary expressions in the cortex and thalamus as well (*10*). Therefore, we examined these types of excitatory synapses on iSPNs. To evaluate presynaptic development, we examined the densities and sizes of VGLUT1 and VGLUT2 puncta on iSPN dendrites in the anterior and posterior striatum. iSPN dendrites were labeled with tdTomato by mating with *Rosa^STOP-tdT^* mice (tdT). The density of VGLUT1 puncta on iSPN dendrites was decreased both in the anterior (Fig. 5A; *P* < 0.0001; MD = −9.99) and posterior (Fig. 5B; *P* = 0.0023; MD = −5.31) striatum of *Pcdh17* cKO (tdT) mice compared to control (tdT) mice. The size of VGLUT1 puncta was only decreased in the anterior striatum (Fig. 5, A and B). In *Pcdh10* cKO (tdT) mice, the density of VGLUT1 puncta was also reduced in the anterior (Fig. 5C; *P* = 0.0315; MD = −10.0) and posterior (Fig. 5D; *P* = 0.0080; MD = −15.6) striatum. We then compared the normalized puncta density of VGLUT1 between the anterior and posterior striatum. We found a significant decrease in VGLUT1 density in the anterior striatum relative to the posterior striatum in *Pcdh17* cKO (tdT) mice (fig. S11C; *P* = 0.0371), while it was similar between the anterior and posterior regions in *Pcdh10* cKO (tdT) mice (fig. S11D; *P* = 0.3427).

The density of VGLUT2 puncta was reduced in both the anterior (Fig. 5E; *P* < 0.0001; MD = −16.0) and posterior (Fig. 5F; *P* = 0.0014; MD = −9.67) striatum in *Pcdh17* cKO (tdT) mice. In *Pcdh10* cKO (tdT) mice, the density of VGLUT2 puncta was also reduced in the anterior (Fig. 5G; *P* < 0.0001; MD = −13.5) and posterior (Fig. 5H; *P* < 0.0001; MD = −9.56) striatum. The normalized puncta density of VGLUT2 was comparable between the anterior and posterior regions in *Pcdh17* and *Pcdh10* cKO (tdT) mice (fig. S11, E and F; *P* = 0.3900 and *P* = 0.5756).

To evaluate postsynaptic development, we examined the clustering of PSD-95 along the iSPN dendrites. We found that the density of PSD-95 puncta was decreased both in the anterior (Fig. 5, I and K; *P* = 0.0037 for *Pcdh17* cKO and 0.0367 for *Pcdh10* cKO) and posterior (Fig. 5, J and L; *P* = 0.0290 for *Pcdh17* cKO and 0.0043 for *Pcdh10* cKO) striatum of *Pcdh* cKO (tdT) mice. These results indicate that *Pcdh17* cKO and *Pcdh10* cKO mice show impaired development of excitatory synapses formed onto the iSPNs. The results not only suggest that there appears to be more robust decreases in the areas where each PCDH is highly expressed (e.g., VGLUT1 in *Pcdh17* cKO mice) but also show that changes were observed outside of the regions with high expression, which may reflect circuit-level compensatory mechanisms or be secondary to abnormal cell aggregation (see below). Given that the anterior striatum expresses low levels of PCDH10 and the posterior striatum expresses low levels of PCDH17, the observed changes in VGLUT1/2 could also be attributed to these low but detectable levels of PCDH expression.

**Fig. 6.**
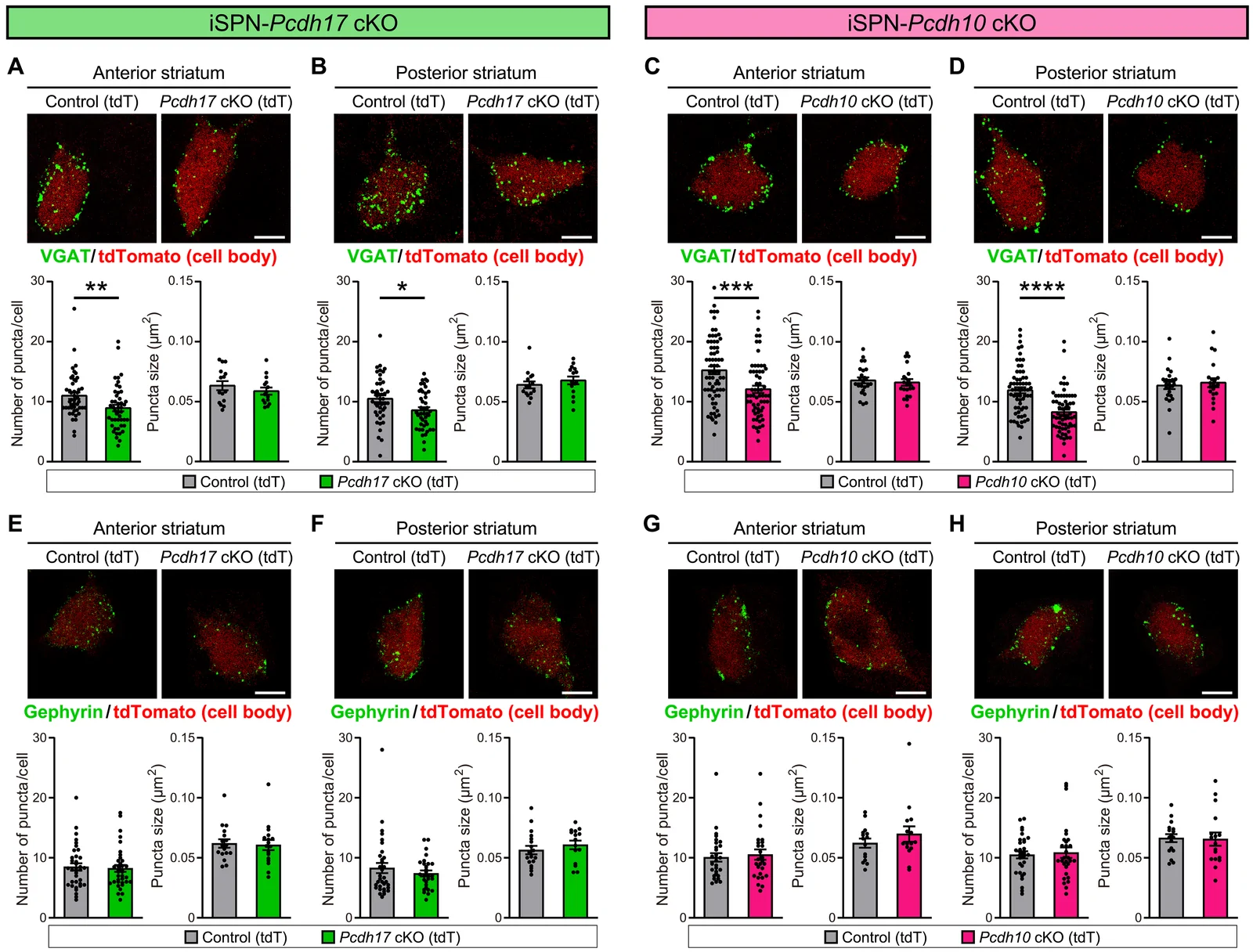
Reduced inhibitory synapses formed on iSPNs in *Pcdh* cKO mice. (**A** to **H**) VGAT (A to D) and Gephyrin (E to H) puncta on tdTomato-labeled iSPN cell bodies in the aStr and pStr of 2-week-old mice. In *Pcdh17* cKO aStr and pStr, VGAT density is decreased (A, *P* = 0.0035; B, *P* = 0.0138). VGAT size remains unchanged (A, *P* = 0.4547; B, *P* = 0.2129). Gephyrin density (E, *P* = 0.8213; F, *P* = 0.7857) and size (E, *P* = 0.9456; F, *P* = 0.2904) remain unchanged. In *Pcdh10* cKO aStr and pStr, VGAT density is decreased (C, *P* = 0.0005; D, *P* < 0.0001). VGAT size remains unchanged (C, *P* = 0.5389; D, *P* = 0.8703). Gephyrin density (G, *P* = 0.8040; H, *P* = 0.7051) and size (G, *P* = 0.5596; H, *P* = 0.5801) remain unchanged. *n* = 15 to 70 fields from four to nine mice per genotype. Scale bars, 2 μm. *P < 0.05, **P < 0.01, ***P < 0.001, and ****P < 0.0001 by Mann-Whitney *U* test.

To investigate the functional consequences of the synaptic defects in *Pcdh17* cKO and *Pcdh10* cKO mice, we recorded spontaneous excitatory postsynaptic currents (sEPSCs) from iSPNs (labeled with tdT) in the anterior and posterior striatum. In *Pcdh17* cKO (tdT) mice, the amplitude of sEPSCs was significantly reduced in the anterior striatum (Fig. 5M; *P* = 0.0431), but not in the posterior striatum (Fig. 5N). The frequency of sEPSCs was not changed in the anterior striatum in *Pcdh17* cKO (tdT) mice; however, it was increased in the posterior striatum. The EPSC frequency can be affected by various factors, including the number of synapses and neurotransmitter release probability. In the posterior striatum of *Pcdh17* cKO mice, compensatory presynaptic changes may have increased neurotransmitter release probability or vesicle release efficiency, thereby increasing the EPSC frequency despite a reduction in synapse number. In *Pcdh10* cKO (tdT) mice, the frequency of sEPSCs was significantly decreased in the posterior striatum (Fig. 5P; *P* = 0.0422), but not in the anterior striatum (Fig. 5O). The amplitude of sEPSC was not changed in *Pcdh10* cKO (tdT) mice. These results indicate that synaptic currents from iSPNs are altered both in *Pcdh17* cKO and *Pcdh10* cKO mice, but the changes are different between them. The complex physiological changes may be because PCDH17 and PCDH10 are involved in both excitatory and inhibitory synapse development (Figs. 5 and 6). In addition, iSPNs in the anterior and posterior striatum may be influencing each other. One may also ask if the abnormal aggregation of iSPNs (Fig. 3) is affecting their physiology. It should be noted that we recorded sEPSCs from iSPNs in striatal regions where PCDH10 and PCDH17 show the highest expression specificity, regardless of the D1R/D2R clustering patterns. We also recorded sEPSCs from dSPNs (identified as tdTomato-negative cells) and found no significant changes in the same regions where iSPNs exhibited phenotypes. Specifically, in the anterior striatum, sEPSC amplitude in dSPNs remained unchanged [*Pcdh17* control; 22.19 ± 3.60 pA, *Pcdh17* cKO; 21.61 ± 2.36 pA, *P* = 0.852 (Student’s *t* test)]. Similarly, in the posterior striatum, the sEPSC frequency in dSPNs showed no significant differences in either *Pcdh17* cKO [*Pcdh17* control; 0.482 ± 0.429 Hz, *Pcdh17* cKO; 0.610 ± 0.505 Hz, *P* = 0.294 (Student’s *t* test)] or *Pcdh10* cKO mice [*Pcdh10* control; 0.618 ± 0.609 Hz, *Pcdh10* cKO; 0.537 ± 0.379 Hz, *P* = 0.504 (Student’s *t* test)]. These data suggest that abnormal clustering may not play a critical role in the observed synaptic phenotypes.

We next analyzed short-term synaptic plasticity at cortico-striatal excitatory synapses in *Pcdh17* cKO mice, as global *Pcdh17* KO mice have been shown to exhibit altered short-term synaptic plasticity (*10*). We found that, while the paired-pulse ratio (PPR) was not significantly different between control and *Pcdh17* cKO mice (fig. S14A), synaptic depression by prolonged repetitive stimulation was significantly weaker in *Pcdh17* cKO mice compared to controls (fig. S14B; main effect, *P* = 0.0493; interaction, *P* = 0.0002). These results suggest that, same as global *Pcdh17* KO mice, *Pcdh17* cKO mice show impaired presynaptic short-term plasticity.

We then examined inhibitory synapses on iSPNs. For this, we quantified the densities and sizes of VGAT and Gephyrin puncta on iSPN cell bodies because many inhibitory synapses are formed around iSPN cell bodies. We found that the density of VGAT puncta on iSPN cell bodies was decreased both in the anterior (Fig. 6A; *P* = 0.0035; MD = −2.06) and posterior (Fig. 6B; *P* = 0.0138; MD = −1.91) striatum of *Pcdh17* cKO (tdT) mice. In *Pcdh10* cKO (tdT) mice, the density of VGAT puncta was also decreased in the posterior striatum (Fig. 6D; *P* < 0.0001; MD = −3.64) and the anterior striatum (Fig. 6C; *P* = 0.0005; MD = −3.18). The normalized puncta density of VGAT on iSPNs was comparable between the anterior and posterior regions in *Pcdh17* cKO (tdT) mice (fig. S11G; *P* = 0.7646), while a modest reduction was observed in the posterior regions compared to the anterior regions in *Pcdh10* cKO (tdT) mice (fig. S11H; *P* = 0.0963). The density and size of Gephyrin puncta were similar in *Pcdh17* cKO and *Pcdh10* cKO mice compared to their control mice (Fig. 6, E to H). These results suggest that *Pcdh17* cKO and *Pcdh10* cKO mice show impaired presynaptic development of inhibitory synapses on iSPNs. In summary, these anatomical and physiological findings demonstrate that PCDH17 and PCDH10 contribute to the establishment of both excitatory and inhibitory synapses onto iSPNs.

### Impaired task learning and behavioral flexibility in *Pcdh17* cKO mice, but not in *Pcdh10* cKO mice

Given the differentially impaired synaptic development of the indirect BG circuits in *Pcdh17* cKO and *Pcdh10* cKO mice, we next investigated the behavioral consequences of *Pcdh* inactivation to determine what kind of behavior is regulated by the circuits organized by PCDH17 and PCDH10. For this, we performed several behavioral paradigms associated with BG functions using 2- to 4-month-old male mice, including task learning (*26*), motor and sensory behaviors (*27*), and anxiety-related behaviors (*28*).

**Fig. 7.**
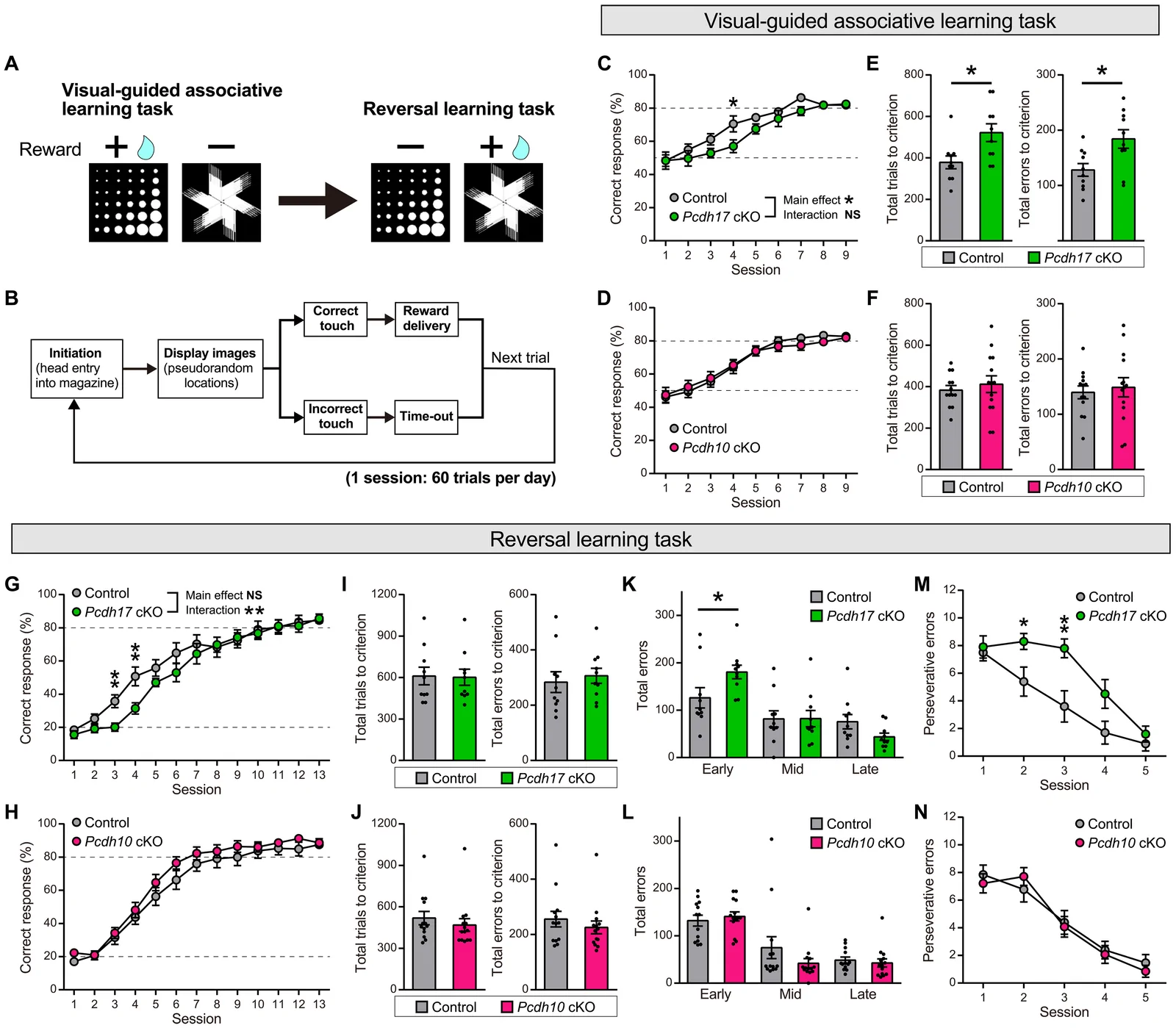
*Pcdh17* cKO but not *Pcdh10* cKO mice show impaired task learning. (**A**) Task schematics. (**B**) Trial timeline. (**C** to **F**) Visual-guided associative learning task. (C and D) Correct response is decreased in *Pcdh17* cKO (C; session 4: *P* = 0.0427; main effect: *P* = 0.0489; interaction: *P* = 0.2914), but not in *Pcdh10* cKO (D; main effect: *P* = 0.8281; interaction: *P* = 0.8392). (E and F) Total trials and errors are increased in *Pcdh17* cKO (E; trials: *P* = 0.0143; errors: *P* = 0.0132), but not in *Pcdh10* cKO (F; trials: *P* = 0.5453; errors: *P* = 0.6695). (**G** to **N**) Reversal learning task. (G and H) Correct responses are reduced in *Pcdh17* cKO (G; sessions 3 and 4: *P* = 0.0033, 0.0090; main effect: *P* = 0.2346; interaction: *P* = 0.0044), but not in *Pcdh10* cKO mice (H; main effect: *P* = 0.2157; interaction: *P* = 0.9238). (I and J) Total trials and errors are unchanged in *Pcdh17* cKO (I; trials: *P* = 0.9210; errors: *P* = 0.6224) and in *Pcdh10* cKO (J; trials: *P* = 0.4571; errors: *P* = 0.4164). (K and L) Total errors are increased in the early phase in *Pcdh17* cKO (K; early: *P* = 0.0489; middle: *P* = 0.9806; late: *P* = 0.0791), but not in *Pcdh10* cKO (L; early: *P* = 0.5604; middle: *P* = 0.1909; late: *P* = 0.5815). (M and N) *Pcdh17* cKO made more perseverative errors in sessions 2 and 3 (*P* = 0.0270, 0.0051). *n* = 10 mice for *Pcdh17* control, 10 for *Pcdh17* cKO, 13 for *Pcdh10* control, and 14 for *Pcdh10* cKO. Two- to 4-month-old male mice were used. *P < 0.05 and **P < 0.01 by Student’s *t* test (C to N) and two-way repeated-measures analysis of variance (ANOVA), followed by Šidák test (C, D, G, and H). NS, not significant.

To evaluate task learning, we used an operant learning system, a reward-based learning system that has been used to assess many types of goal-directed learning, cognitive, and executive functions (*29*, *30*). The mice first went through three stages of pretraining: acclimation to the operant chamber, reward acquisition training, and stimulus touch training (fig. S15, A to F). During pretraining, days to criterion and reaction times (reward collection latency and touch latency) were similar between iSPN-*Pcdh* cKO and control mice, indicating that neither cKO mice have abnormalities in simple reward-based behaviors. Mice that met the criterion in pretraining then proceeded to the pairwise visual discrimination (perception-based cognitive learning) task. In this task, mice were rewarded when they touched the correct image from a pair of visual images (Fig. 7A; visual-guided associative learning task). Mice performed 60 trials (= one session) per day (Fig. 7B). We found that *Pcdh17* cKO mice acquired the task more slowly than controls (Fig. 7C; main effect, *P* = 0.0489; interaction, *P* = 0.2914). They required more total trials and made more total errors before reaching the learning criterion (average correct response of 80% for two consecutive days) compared to control mice (Fig. 7E). On the other hand, *Pcdh10* cKO mice are comparable to control mice in terms of the percentage of correct responses across sessions (Fig. 7D) and the total number of trials and total errors to reach the criterion (Fig. 7F). Neither *Pcdh17* cKO nor *Pcdh10* cKO mice showed any change in the correct response latency compared to their controls (fig. S15, G and H), suggesting that the response speed is normal in both *Pcdh* cKO mice. Together, *Pcdh17* cKO mice, but not *Pcdh10* cKO mice, show impaired perception-based cognitive learning.

After the mice achieved the criterion in the visual-guided associative learning task, the reward contingency of stimulus images was reversed to investigate the capacity for updating learning based on behavioral flexibility (reversal learning task; Fig. 7A). The correct response rate started at ∼20% as expected and gradually increased with repeated training (Fig. 7, G and H). We separated the reversal performance phases into three phases (*31*): the low accuracy phase in the early reversal sessions (early: 0 to 40% correct response), the chance-level phase around the midpoint of reversal sessions (mid: 40 to 60% correct), and the high accuracy phase in the late reversal sessions (late: 60 to 80% correct). *Pcdh17* cKO mice exhibited a transient performance deficit during the early phase (sessions 3 and 4) (Fig. 7G; main effect, *P* = 0.2346; interaction, *P* = 0.0044) relative to the control mice; however, *Pcdh17* cKO mice caught up later, and as a result, the total trials and total errors to meet the final criterion are similar between the cKO and control (Fig. 7I). Consistently, *Pcdh17* cKO mice exhibited increased errors in the early phase but not in the mid or late phases (Fig. 7K). In contrast, *Pcdh10* cKO mice performed similarly to control mice in terms of the percentage of correct responses (Fig. 7H) and total trials and errors to reach the criterion (Fig. 7J). *Pcdh10* cKO mice did not differ in errors in any reversal performance phases compared to control mice (Fig. 7L). These results indicate a slower learning rate in the early phase of reversal learning in *Pcdh17* cKO mice, but not in *Pcdh10* cKO mice. We then analyzed perseverative errors (four consecutive errors were counted as one perseverative error) during the early sessions of reversal learning (sessions 1 to 5), which represent a tendency for persistent and inflexible behavior following an incorrect response (*32*). While *Pcdh10* cKO mice do not show a difference in perseverative errors compared to control mice (Fig. 7N), *Pcdh17* cKO mice made more perseverative errors in sessions 2 and 3 than control mice (Fig. 7M), suggesting that *Pcdh17* cKO mice exhibit reduced behavioral flexibility. Neither *Pcdh17* cKO nor *Pcdh10* cKO mice showed a difference in correct response latency compared to control mice (fig. S15, I and J), indicating that both *Pcdh* cKO mice showed a normal reaction speed to the correct stimulus. Together, iSPN-*Pcdh17* cKO mice show impaired task learning and behavioral inflexibility, while iSPN-*Pcdh10* cKO mice show normal learning and flexibility.

### Reduced locomotor and sensory habituation in *Pcdh10* cKO mice, but not in *Pcdh17* cKO mice

**Fig. 8.**
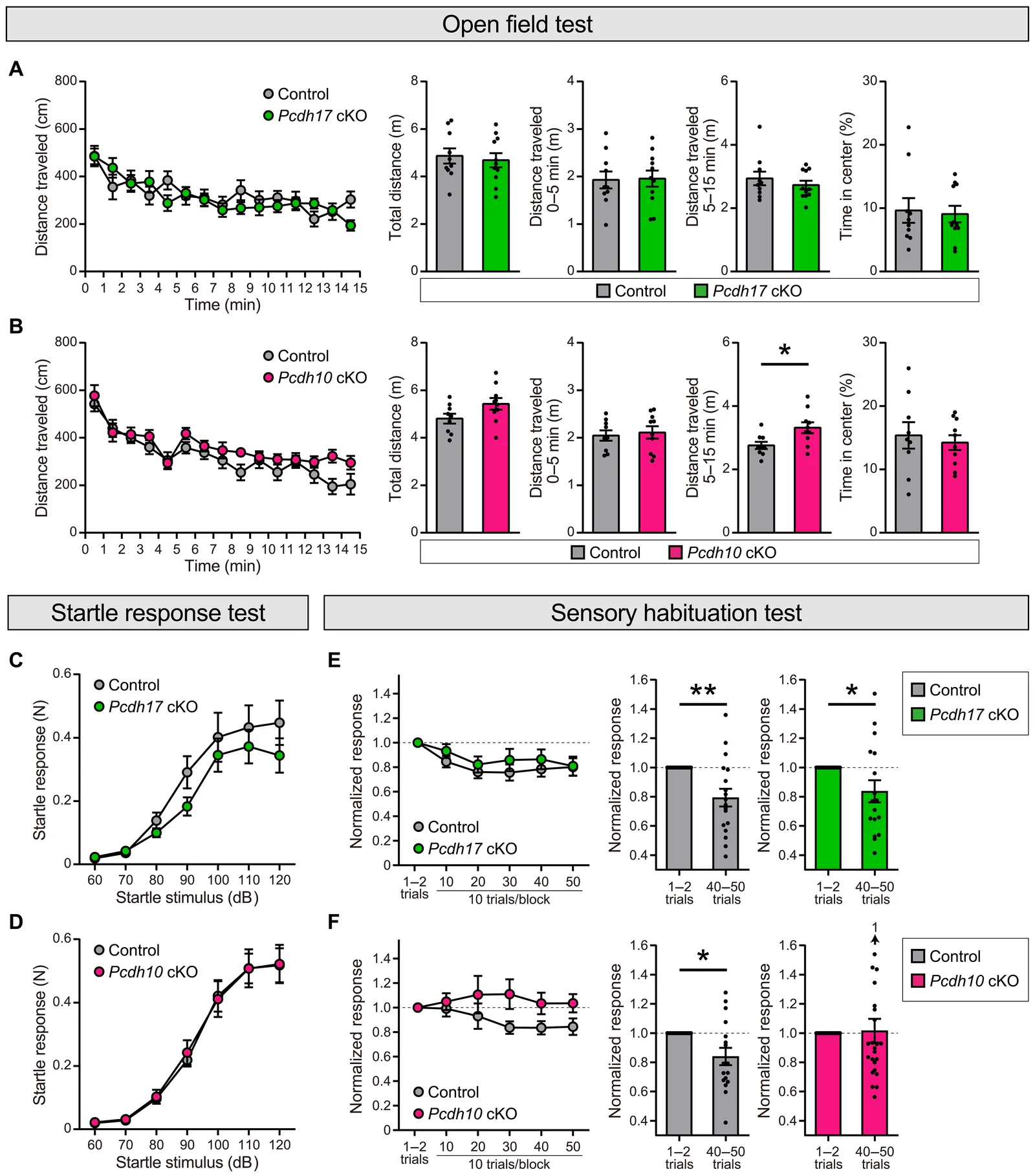
*Pcdh10* cKO but not *Pcdh17* cKO mice show impaired locomotor and sensory habituation. (**A** and **B**) Open field test. The distance traveled in each minute during the 15-min test session, total distance for 15 min (*Pcdh17*; *P* = 0.6848, *Pcdh10*; *P* = 0.0700), total distance for 0 to 5 min (before habituation, *Pcdh17*; *P* = 0.9062, *Pcdh10*; *P* = 0.7076), total distance for 5 to 15 min (after habituation, *Pcdh17*; *P* = 0.4138, *Pcdh10*; *P* = 0.0172), and time spent in the center area (*Pcdh17*; *P* = 0.8048, *Pcdh10*; *P* = 0.6298). The total distance for 5 to 15 min is increased in *Pcdh10* cKO mice relative to control mice. *n* = 10 mice for *Pcdh17* control, 11 for *Pcdh17* cKO, 9 for *Pcdh10* control, and 10 for *Pcdh10* cKO. (**C** and **D**) Startle response test. Startle responses for different startle stimuli are similar between *Pcdh17* cKO and control mice (C) and between *Pcdh10* cKO and control mice (D). *n* = 11 mice for *Pcdh17* control, 9 for *Pcdh17* cKO, 11 for *Pcdh10* control, and 11 for *Pcdh10* cKO. (**E** and **F**) Sensory habituation test. Time course of normalized startle responses (left) and the comparison of startle responses at the beginning (one to two trials) and the end (40 to 50 trials) of trials (right). *Pcdh17* control (*P* = 0.0017), *Pcdh17* cKO (*P* = 0.0397), and *Pcdh10* control (*P* = 0.0104), but not *Pcdh10* cKO mice (*P* = 0.8452), show decreased normalized responses at the end of trials. *n* = 18 mice for *Pcdh17* control, 17 for *Pcdh17* cKO, 17 for *Pcdh10* control, and 24 for *Pcdh10* cKO. Two- to 4-month-old male mice were used. *P < 0.05 and **P < 0.01 by Student’s *t* test.

We next assessed motor and sensory functions. To examine spontaneous locomotor activity in a novel environment, we performed the open field test. We monitored the distance traveled in the open field box (Fig. 8, A and B). Because the travel distance decreases with habituation to a new environment, the distance traveled was also quantified for the first 5 min (before habituation) and the next 5 to 15 min (after habituation). All these parameters were similar between *Pcdh17* cKO mice and control mice (Fig. 8A), suggesting that spontaneous locomotor activity and habituation are normal in *Pcdh17* cKO mice. *Pcdh10* cKO mice also showed a similar distance traveled for the first 5 min compared to control mice. However, *Pcdh10* cKO mice showed a significantly higher distance traveled during the 5- to 15-min period (Fig. 8B) than control, suggesting that locomotor habituation in the novel environment was reduced in *Pcdh10* cKO mice.

We then examined sensory habituation of the acoustic startle response. Acoustic startle responses to different startle stimuli were similar between *Pcdh* cKO and control mice (Fig. 8, C and D), suggesting that both *Pcdh17* cKO and *Pcdh10* cKO mice have a normal response to sensory stimulation. Repeated acoustic stimulation causes a decreased startle response, referred to as “habituation of the acoustic startle response” (*33*, *34*). We found that the startle responses after 40 to 50 trials in *Pcdh17* control, *Pcdh17* cKO, and *Pcdh10* control mice were significantly lower than those of the initial (the first and second) trials (Fig. 8, E and F), indicating that these mice show sensory habituation of the acoustic startle response. In contrast, *Pcdh10* cKO mice exhibited a similar level of startle response even after 40 to 50 trials (Fig. 8F), indicating that they show impaired sensory habituation.

Last, we investigated whether impaired habituation in *Pcdh10* cKO mice is due to changes in their anxiety level. In the open field test, time spent in the center area was similar between *Pcdh10* cKO and control mice and between *Pcdh17* cKO and control mice (Fig. 8, A and B, right). In the elevated plus maze, relative to control mice, *Pcdh10* cKO and *Pcdh17* cKO mice spent a similar amount of time in the open arms (fig. S16, A and B). In the light-dark transition test, the time spent in the light area and the transition number were not significantly different between *Pcdh10* cKO and control mice and between *Pcdh17* cKO and control mice (fig. S16, C and D). These results suggest that the anxiety level of *Pcdh10* cKO mice is normal and that the changes in locomotor and sensory habituation in *Pcdh10* cKO mice are not due to changes in their anxiety levels. In addition, prepulse inhibition, in which a weak stimulus given before the startle stimulus suppresses the startle response, was normal in *Pcdh10* cKO and *Pcdh17* cKO mice (fig. S16, E and F), suggesting that sensorimotor gating abilities are normal in both *Pcdh10* cKO and *Pcdh17* cKO mice. Together, these behavioral experiments revealed that *Pcdh10* cKO mice, but not *Pcdh17* cKO mice, exhibit reduced locomotor and sensory habituation.

## DISCUSSION

Here we showed that in the parallel BG circuits, there are specific molecular organizers that establish individual circuits regulating distinct behaviors. We identified PCDH17 and PCDH10 as molecular organizers for parallel indirect BG circuits. In addition to the indirect BG circuits, these PCDHs show complementary expression in the direct BG circuits (Fig. 1B and fig. S3) as well as in the cortex and thalamus (*10*), suggesting that these PCDHs may organize the entire parallel cortico-BG-thalamo-cortical loop.

### PCDH17 and PCDH10 define functionally distinct and anatomically segregated parallel BG circuits

Functionally distinct and anatomically segregated parallel neural circuits are thought to underlie parallel information processing in the brain (*35*, *36*). However, how individual circuits are organized was largely unknown, and the molecular mechanisms that establish parallel circuits remain to be elucidated. In the hippocampal circuits, complementary expression of teneurin-3 (Ten3) and latrophilin-2 (Lphn2) has been shown to regulate anatomically segregated circuits (*8*, *37*, *38*) via reciprocal repulsions between these two molecules. However, it is not yet clear whether Ten3 and Lphn2 specify functionally distinct circuits regulating different behaviors. In the BG, it has recently been demonstrated that anatomically segregated BG circuits allow parallel behavioral modulation in mice (*3*). However, how these circuits are established was not known. In the present study, we demonstrated that PCDH17 and PCDH10 not only anatomically define parallel indirect BG circuits but also contribute to the functional circuit organization associated with task learning and sensorimotor habituation, respectively. Based on the features of the complementary expression and selective homophilic interaction of PCDH17 and PCDH10, parallel BG circuits allow for specific synaptic control, thereby appropriately regulating different behaviors. Thus, PCDH17 and PCDH10 are molecular organizers by which functionally distinct and anatomically segregated parallel circuits are organized for parallel information processing in the brain.

The parallel BG circuit architecture defined by PCDH17 and PCDH10 represents an additional molecular dimension that intersects with, and extends beyond, established anatomical and molecular frameworks (Fig. 1) (*15*, *16*). Lazaridis *et al.* (*16*) identified a striosome pathway that targets SNc dopamine neurons—especially the “dendron bouquets”—via the central GPe. Consistent with this, we found that PCDH17 is enriched in striosomes and the inner GPe, and localized at these bouquets, the hallmark of striatal projections to dopamine neurons. However, the PCDH17/10 code provides a broader structural framework. Both molecules are robustly expressed in the matrix SPNs of the anterior and posterior striatum, respectively, suggesting that they organize entire parallel loops rather than being restricted to a striosome-specific bypass. Furthermore, the PCDH17/10-defined “inner-outer” GPe organization does not coincide with established molecular subtypes aligned with the medial-lateral axes defined by Lhx6 and PV. While Evans *et al.* (*15*) demonstrated that Lhx6^+^ GPe neurons provide potent inhibitory control over SNc neurons, our results show that PCDH17/10-positive populations are cellularly independent of these markers. The PCDH-defined inner-outer GPe organization aligns with the distribution of Calbindin; however, the PCDH expression patterns do not align with the Calbindin expression in the striatum, where Calbindin is a marker for the matrix (*13*). In the SN, PCDH17 is distributed in both the dopamine dendron bouquets and the posterior SNr, while PCDH10 is localized to the anterior SNr. These findings indicate that PCDH17 and PCDH10 do not serve as markers for known compartments. Instead, these δ2-PCDHs define a distinct adhesion-based layer of BG circuit organization.

### PCDH17 and PCDH10 as synaptic organizers: Potential molecular mismatch and circuit disruption

Global *Pcdh17* KO mice have been reported to show increased synaptic vesicles in the anterior striatum and inner GPe (*10*), while our study with iSPN-*Pcdh17* cKO mice showed impaired development of synapses. This difference may arise from differences between global and conditional KO models. In global *Pcdh17* KO mice, PCDH17 is deleted in all cell types, potentially triggering compensatory mechanisms that restore or even overcompensate for synaptic phenotypes. In contrast, deleting PCDH17 in specific neurons (*Pcdh17* cKO) may prevent such compensatory responses. In iSPN-*Pcdh17* cKO mice, PCDH17 is absent from the postsynaptic side in the striatum and the presynaptic side in the GP, creating a mismatch where PCDH17-expressing cells interact with nonexpressing neurons. Studies on PCDH19 and other δ2-PCDHs have shown that these mismatched interactions significantly affect cell adhesion and synaptic development (*39*–*41*). Therefore, in iSPN-specific *Pcdh17* cKO mice, this mismatch may have resulted in a more severe effect than complete deletion. This idea is further supported by our in vitro studies. The finding that PCDH17 and PCDH10 are not sufficient to induce de novo synapse formation in heterologous cocultures (fig. S13) indicates that their primary function is not as simple synaptogenic triggers. Instead, their role in synaptic development appears to be strictly dependent on precise matching between pre- and postsynaptic sites. This notion is supported by our previous studies demonstrating that the unilateral overexpression of PCDH17 (on either the pre- or postsynaptic side alone) does not increase, but rather reduces the number of synaptic vesicles and dendritic spines (*10*, *42*). These phenotypes suggest that asymmetric expression of PCDHs disrupts synaptic integrity rather than promoting synaptogenesis. Therefore, these δ2-PCDHs may contribute to the stabilization of synapses through this precise matching mechanism.

### Fine-tuning of the parallel indirect BG circuits by PCDHs

PCDH17 and PCDH10 organize parallel indirect BG circuits along the anteroposterior axis in the striatum to the inner-outer axis in the GPe. However, the indirect BG circuits appear more complex—it has been reported that there are 36 domains in the GPe that receive different projections from the striatum (*6*). Thus, additional molecules may exist to fine-tune the parallel indirect BG circuits. The expression analysis of Cadherin superfamily members, including δ1- and δ2-PCDH members, revealed region-specific expression patterns along different axes in the BG (*43*). Their expression patterns are relatively complementary, but there is some overlap. We also identified a small population of neurons coexpressing *Pcdh17* and *Pcdh10* within BG subregions. Our immunostaining shows that in the GPe, these dually expressing neurons are primarily localized between the PCDH17-positive (inner) and PCDH10-positive (outer) domains. Similarly, in the striatum, coexpressing neurons occupy the boundary between the anterior (PCDH17) and posterior (PCDH10) regions (Fig. 1). This double-positive population may have distinct functional implications. First, *Pcdh17*/*Pcdh10* double-positive striatal neurons may preferentially connect with target neurons that also coexpress both PCDHs, thereby forming molecularly matched subcircuits. Second, these double-positive neurons might act as bridging populations that interact with both *Pcdh17*-positive and *Pcdh10*-positive neurons, mediating cross-talk between the PCDH17 and PCDH10 pathways. Previous studies have shown that the adhesive properties of δ-PCDHs are tuned by the combinatorial diversity of δ-PCDH expression in neurons (*41*, *44*). Thus, it is possible that the combinatorial diversity of δ-PCDH expressions defines a more detailed parallel circuit structure and produces more diverse circuit functions in the BG.

In addition, clustered PCDHs encoded by the *Pcdh*α, *Pcdh*β, and *Pcdh*γ gene clusters are capable of imparting diverse molecular identities at the cell surface through combinatorial expression (*45*). It has been shown that patterned clustered PCDH expression in cortical neurons regulates the fine organization of neural circuits (*46*). Clustered PCDHs also regulate dendritic self-avoidance through homophilic repulsion (*47*). In contrast, our results indicate that the loss of PCDH17 and PCDH10 does not disrupt dendritic self-avoidance (i.e., no change in dendritic crossing, fig. S9), suggesting that the function of δ2-PCDHs in vivo is not repulsive and differs from that of clustered PCDHs. This highlights the functional diversity of the PCDH family and suggests that multiple PCDH family members may cooperate to fine-tune the organization of parallel BG circuits.

### Potential roles of PCDH17 and PCDH10 in distinct neuropsychiatric disorders

iSPN-*Pcdh17* cKO mice showed deficits in task learning and behavioral flexibility, and iSPN-*Pcdh10* cKO mice showed defects in locomotor and sensory habituation. Are they related to human disorders? In humans, PCDH17 and PCDH10 are implicated in different disorders. The human *PCDH17* gene is associated with mood disorders, including bipolar disorder (BPD) and major depressive disorder (MDD) (*42*). Patients with MDD tend to make poor decisions, which often have a negative impact on their lives (*48*). BPD is associated with impaired decision-making in terms of impulsivity and risk-taking (*49*). In contrast, the human *PCDH10* gene is linked to ASD and OCD (*50*–*52*). While the core symptoms of ASD include decreased social motivation, language problems, and restricted behaviors (*53*), many ASD patients also exhibit abnormal sensory sensitivity, described as both hyper- and hyporeactivity (*54*). Patients with OCD also often exhibit abnormal sensory processing and a reduced ability to filter stimuli that most people do not find bothersome (*55*). While further research is needed, these disease symptoms are consistent with the idea that PCDH17 and PCDH10 mutations may contribute to symptoms of BPD/MDD and ASD/OCD, respectively, through the impairment of distinct indirect BG circuits.

### Limitations of the study and future directions

In this study, we showed that there is no abnormality in the axonal projection patterns of *Pcdh17-* and *Pcdh10-*expressing iSPNs or in the dendritic morphology of iSPNs and dSPNs in *Pcdh17* cKO and *Pcdh10* cKO mice. Therefore, we tentatively conclude that the main defect contributing to the behavioral changes is the synaptic impairment by *Pcdh17-* and *Pcdh10-*expressing iSPNs. However, the abnormal aggregation of SPNs observed in the cKO mice could have secondary effects on circuit function. In addition, there could be other changes that might affect behavioral changes, such as differences in dopamine secretion patterns. Furthermore, *A2A-Cre* mice can induce recombination not only in the striatum but also in the cortex and hippocampus, which may also contribute to the phenotypes. In future studies, we plan to use optogenetic and chemogenetic strategies to more directly implicate the circuits organized by PCDH17 or PCDH10 in specific behaviors.

## MATERIALS AND METHODS

The experimental procedures described below, including histological, imaging, electrophysiological, and behavioral analyses, were based on protocols established in our previous studies (*10*, *24*, *39*) and were adapted for the current study.

### Mice

*Pcdh17* and *Pcdh10 flox-EGFP*, cKO *EGFP*, *EGFP knock-in*, *flox*, and cKO mice were generated in our laboratory (see fig. S4 and below). *ACTB-FLPe* (the Jackson Laboratory, JAX:003800) (*56*), *A2A-Cre* (Mutant Mouse Regional Resource Centers, MMRRC_036158) (*17*), *Rosa-STOP-tdTomato* (a kind gift from Dr. Fan Wang, MIT) (*57*), *Rbp4-Cre* (Mutant Mouse Regional Resource Centers, MMRRC_037128) (*58*), and *Slc17a7-Cre* (the Jackson Laboratory, JAX:037512) (*58*) mice were described previously. Mice were maintained in standard housing conditions on a 12-hour light/12-hour dark cycle with food and water provided ad libitum. All animal care and use were in accordance with the institutional guidelines and approved by the Institutional Animal Care and Use Committees at Boston Children’s Hospital (13-11-2528, 16-09-3292R, 19-08-4000R, 00001725, and 00002637) and Waseda University (A23-037, A24-047, A25-031, and A26-114). All animal experiments were performed in accordance with the ARRIVE (Animal Research: Reporting of In Vivo Experiments) guidelines. The completed ARRIVE checklist is provided.

### Antibodies

Rabbit and guinea pig polyclonal anti-PCDH17 antibodies and rabbit and rat polyclonal anti-PCDH10 antibodies were described previously (*10*, *24*). The PCDH17 and PCDH10 antibodies were used at 1 to 4 μg/ml. The following primary antibodies were also used: rabbit anti-MOR (1:500; Nittobo Medical, MOR-Rb-Af240), rabbit anti-Calbindin (1:4000; Nittobo Medical, Calbindin-Rb-Se0.1), rabbit anti-PV (1:500; Nittobo Medical, PV-Rb-Af750), mouse anti-Lhx6 (1:500; Santa Cruz Biotechnology, sc-271433; RRID:AB_10649856), rabbit anti-TH (1:2000; EMD Millipore, AB152; RRID:AB_390204), chicken anti-GFP (1:2000; Aves Labs, GFP-1010; RRID:AB_10000240), mouse anti-NeuN (1:1000; Sigma-Aldrich, MAB377; RRID:AB_2298772), rabbit anti-NeuN (1:1000; GeneTex, GTX133127; RRID:AB_2886836), guinea pig anti-D1R (1:1000; Nittobo Medical, D1R-GP-Af500), rat anti-D1R (1:1000; Sigma-Aldrich, D2944; RRID:AB_1840787), rabbit anti-D2R (1:1000; Nittobo Medical, D2R-Rb-Af960), rabbit anti-dsRed (1:500; Clontech, 632496; RRID:AB_10013483), guinea pig anti-VGLUT1 (1:5000; EMD Millipore, AB5905; RRID:AB_2301751), guinea pig anti-VGLUT2 (1:1000; Nittobo Medical, VGLUT2-GP-Af810), guinea pig anti-VGAT (1:1000; Nittobo Medical; VGAT-GP-Af1000), rabbit anti-VGAT (1:1000; Synaptic Systems, 131003; RRID:AB_887869), mouse anti–PSD-95 (1:1000; NeuroMab, 75-028; RRID:AB_2292909), and mouse anti-Gephyrin (1:500; Synaptic Systems, 147021; RRID:AB_2232546). The following secondary antibodies (Thermo Fisher Scientific) were used for immunohistochemistry: donkey anti-rabbit immunoglobulin G (IgG) Alexa Fluor 488 (A-21206; RRID:AB_2535792), goat anti-rabbit IgG Alexa Fluor 488 (A-11034; RRID:AB_2576217), Alexa Fluor 568 (A-11036; RRID:AB_10563566), Alexa Fluor 647 (A-21245; RRID:AB_2535813), DyLight 405 (35551; RRID:AB_1307537), donkey anti-mouse IgG Alexa Fluor 488 (A-21202; RRID:AB_141607), goat anti-mouse IgG Alexa Fluor 594 (A-11032; RRID:AB_2534091), goat anti-guinea pig IgG Alexa Fluor 488 (A-11073; RRID:AB_2534117), Alexa Fluor 568 (A-11075; RRID:AB_141954), Alexa Fluor 647 (A-21450; RRID:AB_2535867), goat anti-chicken IgY Alexa Fluor 488 (A-11039; RRID:AB_142924), and donkey anti-rat IgG Alexa Fluor 488 (A-21208; RRID:AB_2535794), Alexa Fluor 555 (A-78945; RRID:AB_2910652). In addition, donkey anti-mouse IgG Alexa Fluor 488 (Abcam, ab150109; RRID:AB_2571721) and goat anti-mouse IgG Alexa Fluor 647 (Jackson ImmunoResearch, 115-605-146; RRID:AB_2338912) were used.

### DNA constructs

The cDNAs encoding mouse PCDH17 and PCDH10 were cloned into the pEGFP-N vector (Clontech) to generate C-terminal EGFP-tagged fusion proteins, as described previously (*10*). For AAV production, the following plasmids were used: pAAV-hSyn-EGFP-CAAX (Addgene, #208684) to express membrane-targeted EGFP (mEGFP), pAAV-hSyn-Cre (Addgene, #105553) for Cre-dependent recombination, and pAAV-AiE0452h-tdTomato (derived from Addgene, #191708) for iSPN-specific enhancer expression. To package these constructs into AAV vectors, pAdDeltaF6 (Addgene, #112867) was used as the adenoviral helper plasmid. For serotype-specific packaging, pAAV2/1 (Addgene, #112862) was used for AAV1 production, and pUCmini-iCAP-PHP.eB (Addgene, #103005) was used for AAV-PHP.eB production.

### Generation of *Pcdh17* and *Pcdh10* conditional knock-out mice

*Pcdh17* and *Pcdh10 flox-EGFP* mice were generated using the CRISPR-Cas9 system. Two guide RNAs (gRNAs) were designed to remove the first exon of *Pcdh17* or *Pcdh10* from the wild-type allele (see fig. S4). The target sequences of *Pcdh17* gRNAs were 5′-AGGTTCAAACTTGCGTGATG*AGG*-3′ and 5′-TGACCCTGTGTGAATACCAT*TGG*-3′ (the PAM sequence is in italic). The target sequences of *Pcdh10* gRNAs were 5′-CTCCTTTATTCCGACAGTGT*TGG*-3′ and 5′-GAGGGGCCAATCACTGACAA*AGG*-3′. Sigma-Aldrich provided the CRISPR RNA (crRNA) and trans-activating CRISPR RNA (tracrRNA). The donor vector, with a pSV backbone (*59*), contained the loxP-flanked first exon of the *Pcdh* gene and the FRT-flanked EGFP–polyadenylate [poly(A)] cassette, with 5′ and 3′ homology arms spanning ∼4 kb for homology-directed repair. The pSV backbone vector was described previously (*59*). The donor vector, gRNAs, crRNA, and tracrRNA were injected with Cas9 protein (PNA Bio) into the pronucleus of fertilized zygotes (C57BL/6N), and the injected zygotes were transferred into the oviducts of pseudo-pregnant female mice. The positive F0 pups having a flox-EGFP allele were identified by the detection of the 5′ arm and 3′ arm of the *Pcdh17* and *Pcdh10* genes by genomic polymerase chain reaction (PCR), respectively. *Pcdh flox-EGFP* mice were crossed with *ACTB-FLPe* mice to remove the EGFP-poly(A) cassette through FRT/FLP-mediated excision to generate *Pcdh flox* mice. To generate constitutive *Pcdh-EGFP* knock-in mice via germline recombination, we used either *Rbp4-Cre* or *Slc17a7-Cre* mice to remove the first exon of each *Pcdh* allele. These Cre lines have been reported to cause germline recombination when inherited from the father (*58*). Germline recombination was confirmed in offspring from *Rbp4-Cre/Pcdh17^floxE/+^* and *Slc17a7-Cre/Pcdh10^floxE/+^* male mice (see fig. S4, E and F), resulting in the generation of *Pcdh17^EGFP/+^* and *Pcdh10*^EGFP/+^ mice. *Pcdh17* wild-type and conditional alleles (flox-EGFP and flox) were identified using PCR assays in which primers P17Fw (5′-CCTAGCGGGAGTGAGGAAAACCAAGC-3′) and P17Rv (5′-CAAAGCCTTTAGCTTGCCTTCAGCCG-3′) amplified a wild-type fragment and a 5′ loxP-inserted fragment, respectively (see fig. S4C). *Pcdh10* wild-type and conditional alleles were detected using PCR assays in which primers P10Fw (5′-CCCATCAGCAGGAAGACAAACCTAGG-3′) and P10Rv (5′-AGAGCGTCTCCAAATCGAGCCTCATT-3′) amplified a wild-type fragment and a 5′ loxP-inserted fragment (see fig. S4D). The presence of the EGFP sequence was confirmed by PCR using primers GFP-Fw (5′-ATCTTCTTCAAGGACGACGGCAACTACAAG-3′) and GFP-Rv (5′-AGAGTGATCCCGGCGGCGGTCACGA-3′), which yielded a single band. *Pcdh17^EGFP/+^* and *Pcdh10*^EGFP/+^ mice were identified by the presence of both the EGFP sequence and the wild-type *Pcdh* allele, and the absence of the 5′ loxP-inserted fragment, as determined by PCR using the primers described above (see fig. S4, E and F). *Pcdh* cKO mice were generated by mating *Pcdh* flox mice with *A2A-Cre* mice. Immunostaining confirmed decreases in PCDH17 and PCDH10 proteins in *Pcdh* cKO mice (see fig. S6, A and B). *Pcdh17* and *Pcdh10 flox-EGFP* and *flox* mice were backcrossed with C57BL/6J mice at least five times and maintained on a C57BL/6J background. To minimize experimental variability, we used littermates as controls. Specifically, we crossed *Pcdh^flox/+^* males with *Pcdh^flox/+^* females to obtain *Pcdh^flox/flox^* and *Pcdh^+/+^* mice within the same litter for comparison.

### AAV preparation and injections

Recombinant AAVs were produced using AAVpro 293 T cells (Takara Bio, 632273; RRID:CVCL_B0XW) transfected with the plasmids described in the DNA Constructs section using polyethylenimine. For iSPN-specific enhancer AAV, AAV(PHP.eB)-AiE0452h-tdTomato was produced by cotransfecting pAAV-AiE0452h-tdTomato with pAdDeltaF6 and pUCmini-iCAP-PHP.eB. For anterograde transsynaptic labeling, AAV1-hSyn-Cre was produced by co-transfecting pAAV-hSyn-Cre with pAdDeltaF6 and pAAV2/1. For sparse labeling of SPNs via retro-orbital injection, AAV(PHP.eB)-hSyn-EGFP-CAAX was produced by co-transfecting pAAV-hSyn-EGFP-CAAX with pAdDeltaF6 and pUCmini-iCAP-PHP.eB. Five days after transfection, the cells and media containing AAVs were collected, and the cells were lysed. After removing cell debris, AAVs were purified by ultracentrifugation and suspended in phosphate-buffered saline (PBS). To examine region-specific physiological connections, AAV-CAG-Flex-ChR2-tdTomato was coinjected with AAV-CAG-Cre. These AAVs were produced at the Boston Children’s Hospital Viral Core. AAV titers were determined by real-time quantitative PCR. The titers of the viruses used for injections in this study were as follows: AAV(PHP.eB)-AiE0452h-tdTomato [2 × 10^12^ genome copies (GC)/ml], AAV1-hSyn-Cre (5 × 10^13^ GC/ml), AAV(PHP.eB)-hSyn-EGFP-CAAX (1 × 10^12^ to 5 × 10^12^ GC/ml), AAV-CAG-Flex-ChR2-tdTomato (1.0 × 10^13^ GC/ml), and AAV-CAG-Cre (1.0 × 10^13^ GC/ml).

For AAV stereotaxic injections, adult mice were anesthetized with a mixture of three anesthetics: medetomidine hydrochloride (0.3 mg/kg), midazolam (4 mg/kg), and butorphanol tartrate (5 mg/kg). Mice were secured in a stereotaxic frame (Stoelting). A small craniotomy was performed above the target structures, and a 33-gauge injection needle was attached to a 25-μl syringe (Neuros Syringe, Hamilton) was inserted into the target brain regions. The syringe was controlled by a motorized microinfusion pump (Legato 130, KD Scientific). A total of 200 nl of AAV was delivered bilaterally into the anterior and posterior striatum at a constant flow rate of 100 nl/min. The coordinates relative to bregma and the dural surface were as follows: anterior-posterior (AP), +1.6 mm; medial-lateral (ML), ±1.3 mm; dorsal-ventral (DV), –2.5 mm for the anterior striatum and AP, +0.6 mm; ML, ±1.8 mm; and DV, –3.0 mm for the posterior striatum. After the surgery, mice were kept on a heating pad at 37°C until they fully recovered from anesthesia and were then returned to their home cages. For experiments involving iSPN-specific labeling, 7-month-old *Pcdh17^EGFP/+^* and *Pcdh10^EGFP/+^* mice were injected and analyzed 12 days after AAV injection. For anterograde transsynaptic labeling, 2- to 7-month-old *Rosa^STOP-tdT^* mice (both heterozygotes and homozygotes) were used, and mice were analyzed 3 to 4 weeks after AAV infection to allow for sufficient transsynaptic reporter expression. For experiments examining physiological connections, 5- to 7-month-old C57BL/6J mice were injected and analyzed 3 to 4 weeks after AAV injection.

For AAV retro-orbital injections for sparse labeling of SPNs, 6- to 9-week-old mice were anesthetized with a mixture of three anesthetics, including medetomidine hydrochloride (0.3 mg/kg), midazolam (4 mg/kg), and butorphanol tartrate (5 mg/kg). A total of 100 μl of AAV suspended in PBS was injected into the retro-orbital sinus using a 30-gauge needle. Mice were analyzed 2 to 3 weeks after AAV infection.

### In vitro coculture assay

HEK293T cells (AAVpro 293 T cells; Takara Bio) were maintained in Dulbecco’s modified Eagle’s medium supplemented with 10% fetal bovine serum and transfected using polyethylenimine. For primary neuronal cultures, striatal cells were prepared from wild-type E17.5 mice (ICR, males and females). Tissues were dissociated in 0.25% papain and 1% deoxyribonuclease I, and 1 × 10^5^ striatal cells were plated on poly-l-lysine–coated glass coverslips (12-mm diameter, Matsunami Glass) in AscleStem Neuronal Medium with AscleStem Neuronal Supplement (Nacalai Tesque). For coculture experiments, HEK293T cells were transfected with plasmids encoding PCDH17-EGFP, PCDH10-EGFP, or EGFP, when the primary neuronal cultures reached days in vitro (DIV)9. On DIV10, 2000 to 4000 transfected HEK293T cells were seeded onto the primary striatal neurons. The cocultures were maintained for 24 hours to allow for potential synaptic differentiation before being processed for immunocytochemistry.

### Immunocytochemistry

For coculture experiments, neurons and HEK293T cells were fixed with 4% paraformaldehyde (PFA)/4% sucrose in PBS for 15 min at 37°C. Fixed cells were incubated with blocking buffer [2% bovine serum albumin (BSA)/2% goat serum/0.1% Triton X-100 in PBS] for 30 min at room temperature. Fixed cells were then incubated with primary antibodies (anti-VGAT and anti-GFP) in PBS overnight at 4°C, followed by fluorescent secondary antibodies in PBS for 1 hour at room temperature. Between incubation steps, cells were washed three times with PBS. Coverslips were mounted with Fluoromount-G (Electron Microscopy Sciences).

### Immunohistochemistry

Male and female mouse brains were perfused with 4% PFA in PBS, postfixed in 4% PFA in PBS overnight at 4°C, and then cryoprotected in 30% sucrose in PBS at 4°C. For PCDH17 and PCDH10 staining, mouse brains were perfused with 2% PFA in PBS without postfixation. For Lhx6, PCDH17, and PCDH10 staining, mouse brains were perfused with 3% glyoxal/0.8% acetic acid in PBS, as previously described (*60*). These brains were postfixed in the same glyoxal-acetic acid fixative overnight at 4°C and then cryoprotected in 30% sucrose in PBS at 4°C. Brains were frozen and sectioned at 16-μm thickness on a CM3050S or CM1950 cryostat (Leica). For PSD-95 staining, sections were pretreated with pepsin (1 mg/ml) in 0.2 N HCl for 5 min at room temperature before staining. To enhance the dendrite signal for synaptic puncta analysis, sections were costained with anti-dsRed antibodies. Sections were incubated with blocking buffer (2% BSA/2% goat serum/0.1% Triton X-100 in PBS) for 1 hour at room temperature, incubated with primary antibodies in blocking buffer overnight at 4°C, and incubated with fluorescent secondary antibodies (1:500; Thermo Fisher Scientific) in blocking buffer for 2 hours at room temperature. Sections were washed with PBS between incubation steps; however, for glyoxal-fixed tissues, PBS containing 0.1% Triton X-100 was used for all washing steps. Sections were mounted with Fluoromount-G.

### Imaging

#### Imaging of PCDH17 and PCDH10 staining

Images were taken with a BZ-X810 fluorescence microscope (Keyence), an LSM700 confocal microscope (Zeiss), or an FV3000 confocal microscope (Evident). Images were obtained with the identical acquisition settings regarding the exposure time, laser power, detector gain, and amplifier offset. For wide-field imaging, images were obtained by stitching several adjacent images together. The average signal intensity was quantified using ImageJ. The regions of interest (ROIs) analyzed in fig. S6 were the total area of the anterior and posterior striatum, inner and outer GPe, and posterior SNr (PCDH17; fig. S6, A and E) and the total area of the anterior and posterior striatum, inner and outer GPe, and anterior SNr (PCDH10; fig. S6, B and F). In addition, the total areas of the cerebral cortex, hippocampus, and thalamus were analyzed (fig. S6, C and D). Signal intensities in the corpus callosum (for the striatum, cerebral cortex, and hippocampus), internal capsule (for the GPe and thalamus), and cerebral peduncle (for the SNr) were calculated as the background signals. The background signals were subtracted from the signal intensities in the ROIs. For fig. S7 (A and B), single-plane high-resolution images for PCDH17/PCDH10, D1R, and D2R in the anterior and posterior striatum were acquired at a resolution of 1024 × 1024 pixels using a 100× objective lens with 1× zoom. The cell bodies (ROIs) of D1R-positive dSPNs and D2R-positive iSPNs were identified on the basis of the D1R/D2R signal distributions (*21*). The average intensities of PCDH17 or PCDH10 in the anterior and posterior striatum were quantified within the ROIs. Signal intensities in the nuclei of these cells were considered as background and were subtracted from the signal intensities within the ROIs.

#### Quantification of Pcdh17 and Pcdh10 mRNA expression

Double fluorescent in situ hybridization images were analyzed using ImageJ. The signal intensities in the corpus callosum for the quantification in the striatum, internal capsule for GPe/GPi, and cerebral peduncle for SNr were calculated as the background signals. Background-subtracted binary images were prepared. To assess coexpression, *Pcdh17* and *Pcdh10* binary images were overlaid. Cells were manually counted as double positive if the overlap between *Pcdh17* and *Pcdh10* signals occupied more than 10% of the total signal area. Otherwise, *Pcdh*-positive cells were categorized as either *Pcdh10* only or *Pcdh17* only. The percentages of each cell type were calculated, and the results were visualized as pie charts. The numbers of cells analyzed were striatum (1168 cells from two fields), GPe (709 cells from three fields), GPi (187 cells from two fields), and SNr (234 cells from one field).

#### Imaging PCDH17^+^ and PCDH10^+^ inhibitory synapses in the GPe

Sagittal brain sections from *Pcdh17^EGFP/+^* and *Pcdh10^EGFP/+^* mice were immunostained with antibodies against EGFP, VGAT, and NeuN to visualize inhibitory terminals and GPe neurons. To identify structurally apposed synaptic sites, quad-color immunostaining was also performed by including an antibody against the inhibitory postsynaptic marker Gephyrin. Images were taken with an FV3000 confocal microscope (Evident). Images were obtained with the identical acquisition settings regarding the exposure time, laser power, detector gain, and amplifier offset. For fig. S5 (A and C), single-plane images were acquired at a resolution of 1024 × 1024 pixels using a 20× objective lens with 1× zoom; for fig. S5 (B, D, E, and F), images at 1024 × 1024 pixels were acquired as a z-stack (eight optical sections, 0.43-μm step size) using a 60× objective lens with a 2× zoom and subjected to maximum intensity projection. Synaptic targeting in the GPe was quantified with ImageJ. Background intensity for VGAT, Gephyrin, and NeuN was determined using the Auto Threshold plugin. For EGFP, signal intensities within the nuclei of PCDH-negative cells were used to define background levels. Background-subtracted binary images were prepared for all channels. VGAT binary images were overlaid onto EGFP binary images, and EGFP^+^/VGAT^+^ puncta, representing PCDH-positive inhibitory terminals, were extracted. Similarly, EGFP^+^/Gephyrin^+^ puncta were extracted to define PCDH-positive postsynaptic sites. NeuN signals were also binarized to define the neuronal cell body. The number of EGFP^+^/VGAT^+^ puncta per EGFP^+^/NeuN^+^ and EGFP^−^/NeuN^+^ GPe cell body was quantified using ImageJ’s “Analyze Particles” function. Puncta smaller than 5 pixels were excluded from analysis. Structurally apposed synapses, defined by the close apposition of EGFP^+^/VGAT^+^ presynaptic puncta and EGFP^+^/Gephyrin^+^ postsynaptic puncta on the cell body, were manually quantified according to criteria adapted from previously established methods (*24*, *39*).

#### Imaging iSPN-specific inhibitory terminals in the GPe

Sagittal brain sections were prepared from *Pcdh17^EGFP/+^* and *Pcdh10^EGFP/+^* mice that had received injections of AAV(PHP.eB)-AiE0452h-tdTomato into either the anterior or posterior striatum (Fig. 2, C and F). Sections were immunostained with antibodies against EGFP, VGAT, and NeuN to visualize inhibitory terminals and GPe neurons. Images were taken with an FV3000 confocal microscope (Evident) with the identical acquisition settings regarding the exposure time, laser power, detector gain, and amplifier offset. For Fig. 2 (D and G), single-plane images were acquired at a resolution of 1024 × 1024 pixels using a 10× objective lens with 1× zoom; multiple adjacent fields were stitched to visualize the iSPN → GPe pathway. For Fig. 2 (E and H), images at 1024 × 1024 pixels were acquired as a z-stack (eight optical sections, 0.43-μm step size) using a 60× objective lens with a 2× zoom and subjected to maximum intensity projection. iSPN-specific synaptic targeting in the GPe was quantified with ImageJ. Background intensity for VGAT and NeuN was determined using the Auto Threshold plugin. For tdTomato, signal intensities within the nuclei of GPe cells were used to define background levels. Background-subtracted binary images were prepared. VGAT binary images were overlaid onto tdTomato binary images to extract tdTomato^+^/VGAT^+^ puncta, representing inhibitory terminals originating from the anterior or posterior striatal iSPNs, which predominantly consist of PCDH17^+^ and PCDH10^+^ populations, respectively. NeuN signals were also binarized to define the neuronal cell body. The number of tdTomato^+^/VGAT^+^ puncta per EGFP^+^/NeuN^+^ and EGFP^−^/NeuN^+^ GPe cell body was quantified using ImageJ’s “Analyze Particles” function. Puncta smaller than 5 pixels were excluded from analysis.

#### Imaging of anterograde transsynaptic labeling from the striatum to the GPe

Sagittal brain sections were prepared from *Rosa^STOP-tdT^* mice that had received injections of AAV1-hSyn-Cre into either the anterior or posterior striatum (Fig. 2, I and L). Sections were immunostained with antibodies against Calbindin and NeuN to delineate GPe subregions and identify neuronal cell bodies, respectively. Images were taken with an FV3000 confocal microscope (Evident) with the identical acquisition settings regarding the exposure time, laser power, detector gain, and amplifier offset. For Fig. 2 (J and M), single-plane low magnification images were acquired at a resolution of 1024 × 1024 pixels using a 10× objective lens with 1× zoom; multiple adjacent fields were stitched to visualize the iSPN → GPe pathway. The GPe was partitioned into inner and outer domains based on Calbindin immunoreactivity and anatomical landmarks. For Fig. 2 (K and N), single-plane high magnification images in the inner and outer GPe were acquired at a resolution of 1024 × 1024 pixels using a 60× objective lens with 1× zoom. Within these GPe regions, the numbers of NeuN-positive neurons (total neurons) and tdTomato-positive/NeuN-positive neurons (transsynaptically labeled neurons) were manually counted. Labeling efficiency was expressed as the percentage of transsynaptically labeled neurons relative to the total NeuN-positive population (% of tdTomato^+^ NeuN^+^/Total NeuN^+^ neurons).

#### Imaging of D1R and D2R staining

Images were taken with an LSM700 confocal microscope (Zeiss) or an FV3000 confocal microscope (Evident). For each antibody, images were obtained with the identical acquisition settings regarding the exposure time, laser power, detector gain, and amplifier offset. For Fig. 3 (A to D), single-plane images were acquired at a resolution of 1024 × 1024 pixels using a 25× objective lens with 1× zoom; for fig. S8 (A to D), single-plane images were acquired at a resolution of 1024 × 1024 pixels using a 10× objective lens with 1× zoom, and ROIs corresponding to the anterior and posterior striatum were subsequently defined. For each image, the green (D2R) and red (D1R) channels were separated. For each channel, the background was corrected by subtracting the average intensity of three random points from the entire image using ImageJ. The random points were chosen using an online random number generator, which provided three pairs of random numbers between 1 and 1024, where each pair was used as the *x* and *y* coordinates. If a point fell into an area with no signal, then it was replaced with another random point. The pixel intensity arrays were then exported to finish the analysis using Python. Specifically, for the D1R array, the data was smoothed by averaging the intensities over squares measuring 5 × 5 pixels. The averaged intensities were then normalized by setting the maximum intensity to 1, the minimum intensity to 0, and scaling all the other intensities accordingly. The array was then flattened, with each row being concatenated with the previous and following rows, creating a one-dimensional vector containing all the rows of the D1R array. These steps were then repeated with the D2R array. The similarity coefficient was calculated by taking the normalized dot product of the two vectors, which measures how correlated the D1R and D2R intensities are in each 5 × 5 region. The contour maps were plotted in Python using matplotlib. For Fig. 3 (E to H), single-plane images were acquired at a resolution of 1024 × 1024 pixels using a 60× objective lens with 1× zoom. To measure the nearest neighbor distance, dSPNs and iSPNs were visually identified based on D1R and D2R signal distribution around the cell bodies. The cell bodies were then manually plotted using Illustrator (Adobe Systems). Nearest neighbor distances were quantified using the BioVoxxel Toolbox plugin in ImageJ, and the average nearest neighbor distance per image was calculated.

#### Imaging of cell morphologies

For the imaging of cell morphologies of dSPNs and iSPNs in the striatum, mice that underwent retro-orbital injections were perfused with 4% PFA in PBS. Brains were removed, postfixed in 4% PFA in PBS overnight at 4°C, and cryoprotected in 30% sucrose in PBS at 4°C. Thick sections (100 μm) were cut on a CM1950 cryostat and collected in PBS. Sections were washed with PBS and then mounted with Fluoromount-G.

For imaging of cell bodies and dendrites of dSPNs and iSPNs, images of mEGFP and tdTomato fluorescence were taken with an FV3000 confocal microscope from the anterior and posterior striatum. Images at 512 × 512 pixels were acquired as a z-stack (30 optical sections, 1.5 μm step size) using a 20× objective lens with a 1× zoom. Z-stack images were subjected to maximum projection. Images were obtained with the identical acquisition settings regarding the exposure time, laser power, detector gain, and amplifier offset. dSPNs and iSPNs were determined by whether the cell body signal of mEGFP in single-plane images was tdTomato^+^ (iSPNs) or tdTomato^−^ (dSPNs). The cell body size (area) of dSPNs and iSPNs was measured using ImageJ. For the analysis of dendritic crossings, maximum intensity images were inverted and contrast-enhanced using Photoshop (Adobe Systems). Dendritic crossings in single SPNs were manually quantified by counting the number of branch overlaps.

#### Imaging of axon terminals

In *A2A-Cre;Rosa^STOP-tdT^;Pcdh^flox-EGFP^* mice, the EGFP signal inside the GPe visualizes the area of axonal innervation from *Pcdh*-expressing iSPNs. Sagittal sections were stained for EGFP, and whole brain images were obtained by stitching several adjacent images together using a BZ-X810 fluorescence microscope (Keyence) with 4× lens. Image analysis was performed using ImageJ. The signal intensity of the internal capsule was calculated as the background signal and subtracted from that of the GPe. The analysis of axon terminals was done using a 50-pixel wide line scan crossing the GPe (see fig. S10). To consistently localize this line scan, the following steps were taken: The DAPI signal was used to find the granule cell layer of the dentate gyrus (DG). Hereafter, the apex and the anterior end of the upper blade of the DG were connected with a straight line (fig. S10, B and C). Next, a parallel line to the first one was drawn through the geometrical center of the GPe, which was determined using the tdTomato signal (fig. S10, A to D). This line was then used to perform a line scan oriented from the anterior side of the GPe (closer to the striatum) to the posterior side of the GPe (fig. S10, E and F). The highest axonal density was set as 1, and the remaining values were normalized accordingly. Line scans were performed on two to four images per mouse and then averaged. Thereafter, the EGFP signal represented by the average line scan was evaluated by introducing a threshold intensity value. For each mouse, the average EGFP intensity of the medial geniculate nucleus (MGN) was multiplied by the factor 1.5 (fig. S10E, white box), normalized according to the values obtained in the line scan, and used as an individual threshold value defining the border of axonal innervation. At this intensity value, on the *y* axis of the line scan, a horizontal bar was introduced. Doing so yielded two crossing points with the average EGFP-line scan (fig. S10G). The distance from the beginning of the line scan to the first crossing point (distance 1), the distance between the two crossing points (distance 2), and the distance between the second crossing point and the posterior edge of the GPe (end of line scan, distance 3) were determined and used to compare between the genotypes (fig. S10, H, I, K, and L). To evaluate the density of axon terminals in the GPe (fig. S10, J and M), we measured the EGFP intensity in the GPe and divided it by the EGFP intensity in the striatum. For this purpose, in iSPN-*Pcdh17* cKO mice, we measured intensity in the anterior part of the striatum and the inner region of the GPe; for iSPN-*Pcdh10* cKO mice, the posterior part of the striatum and the posterior part of the GPe were assessed.

#### Imaging of synaptic puncta

Images were taken with an LSM700 confocal microscope (Zeiss) and an FV3000 confocal microscope (Evident). For each individual antibody, images were obtained with the identical acquisition settings regarding the exposure time, laser power, detector gain, and amplifier offset. In the striatum, images at 1024 × 1024 pixels were acquired as a z-stack (5 optical sections, 0.5 μm step size) using a 63× objective lens with a 1.5× zoom. Background intensity was removed by using ImageJ’s ‘rolling ball’ algorithm. For VGLUT1, VGLUT2, and PSD-95 in the striatum, and VGAT in the GPe, puncta on dendrites or axons were quantified using the ‘Synapse on Dendrite’ quantifier plugin for ImageJ (*61*). Thereby, puncta density (number of puncta/dendrite area) and puncta size were determined. For the analysis of VGAT and Gephyrin puncta in the striatum, the number of synaptic puncta per tdTomato^+^ cell body, along with the puncta size, was calculated using the Synapse on Dendrite plugin. In the GPe, z-stack settings were adjusted to 12 optical sections with 0.5-μm step size. Background-subtracted images were prepared, and the density and size of puncta were quantified using ImageJ’s ‘analyze particle’ function. Puncta smaller than 5 pixels were excluded from analysis.

#### Imaging of synaptic puncta in coculture experiments

Cells were imaged with an FV3000 confocal microscope (Evident). Images were obtained with the identical acquisition settings regarding the exposure time, laser power, detector gain, and amplifier offset. Images at 1024 × 1024 pixels were acquired as a z-stack (20 optical sections, 1.0 μm step size) using a 60× objective lens with a 3.0× zoom. VGAT-positive puncta were quantified using ImageJ. Background intensity for VGAT was determined using the Auto Threshold plugin, and background-subtracted binary images were prepared. The density and size of VGAT puncta in contact with GFP-labeled HEK293T cells were quantified using the Analyze Particles function. Puncta smaller than 5 pixels were excluded from analysis.

### Electrophysiology

All electrophysiological experiments and analyses were done blind. Recordings were done on male and female mice from ages P12 to P18. Mice were decapitated, brains were quickly removed, and then parasagittal slices (300 μm) were cut using a VT1200S vibratome (Leica). Slices were cut in an ice-cold cutting solution containing 206 mM sucrose, 2.8 mM KCl, 2 mM MgSO_4_, 1 mM MgCl_2_, 1.25 mM NaH_2_PO_4_, 1 mM CaCl_2_, 10 mM glucose, 26 mM NaHCO_3_, and 0.4 mM ascorbic acid. After cutting, slices were moved into room temperature artificial cerebral spinal fluid (aCSF) containing 125 mM NaCl, 2.5 mM KCl, 2 mM CaCl_2_, 1 mM MgCl_2_, 1.25 mM NaH_2_PO_4_, 16.6 mM glucose, 26 mM NaHCO_3_. Recording was then conducted in the same aCSF, at least 1 hour after cutting. All solutions were continuously bubbled with 95% oxygen/5% carbon dioxide. Neurons were visualized using a customized Scientifica/Olympus microscope. Data were obtained with a Multiclamp 700B amplifier (Molecular Devices), digitized with a Digidata 1440A (Molecular Devices), and collected with Clampex 10.7 (Molecular Devices). Whole-cell patch-clamp recordings were conducted with 4- to 6-MΩ pipette containing 120 mM CsMeSO_4_, 15 mM CsCl, 8 mM NaCl, 0.2 mM EGTA, 2 mM Mg–adenosine 5′-triphosphate (ATP), 0.3 mM Na–guanosine 5′-triphosphate (GTP), 10 mM tetraethylammonium, 10 mM Hepes, 5 mM QX-314 (to block sodium channels), and 0.15% lucifer yellow (to fill the cells). The pH of the internal solution was adjusted to 7.3 with CsOH. For sEPSCs, cells were held at −70 mV. The aCSF was supplemented during recordings with 50 μM picrotoxin (to block inhibition) and warmed to 32°C. Last, sEPSCs were analyzed using Minianalysis (Synaptosoft).

To record sIPSCs in the GPe, sagittal sections were prepared as above. We used the same aCSF (supplemented with 25 μM DL-APV and 10 μM CNQX to block excitation) and internal solutions. Neurons were targeted along the edge of the GPe (in the putative PCDH10^+^ region) based upon tdTomato fluorescence. Neurons were held at 10 mV, and sIPSCs were recorded.

For short-term plasticity, parasagittal sections (300 μm) were cut from P11 to P23 mice as described above in an ice-cold solution containing 110 mM choline chloride, 25 mM NaHCO_3_, 2.5 mM KCl, 7 mM MgCl_2_, 0.5 mM CaCl_2_, 1.25 mM NaH_2_PO_4_, 25 mM glucose, 11.6 mM ascorbic acid, and 3.1 mM pyruvic acid. Then, slices were transferred to a solution containing 125 mM NaCl, 2.5 mM KCl, 2 mM CaCl_2_, 1 mM MgSO_4_, 1.25 mM NaH_2_PO_4_, 26 mM NaHCO_3_, and 20 mM glucose. During recording, this solution was also supplemented with 50 μM picrotoxin. Cells were patched in the anterior striatum with an internal solution containing 135 mM CsCl, 10 mM Hepes, 1 mM EGTA, 1 mM Li-GTP, 4 mM Mg-ATP, 5 mM MgCl_2_. Cells were held at −70 mV. Signals were filtered at 3 kHz and digitized at 20 kHz. Two micropipettes were filled with 3 M NaCl and were placed in the cortex at the border of the striatum. The paired pulse ratio was measured with interstimulus intervals of 20, 30, 50, 70, 100, and 200 ms. To measure short-term depression, EPSCs were evoked by 200 stimuli at 10 Hz.

For optogenetic recording, we used the same aCSF and internal solutions as in sEPSC and IPSC recordings (supplemented with 25 μM DL-APV and 10 μM CNQX to block excitation). Cells were held at 10 mV. Responses were evoked by a 5-ms light pulse (470 nm) delivered every 10 s for a total of 5 to 10 sweeps. One to three sweeps were conducted with no light pulse as a control. Cells were targeted on the basis of the presence or absence of red fluorescence. Analysis was based upon whether there was a time-locked consistent response evoked by light stimulation in all sweeps or not.

### Behavioral tests

All data acquisition and analyses were carried out by an individual blind to the genotype. Two- to 4-month-old male mice were used for experiments. Because female mice have estrous cycles that may result in more behavioral variability than male mice (*62*), we used male mice in the behavioral studies.

#### Open field test

Mice were placed in the center of an open-field apparatus [41 cm by 41 cm by 38 cm (*W* × *D* × *H*)] illuminated at 30 lux and allowed to move freely for 15 min. Distance traveled for each 1 min, total distance traveled, distances traveled for the first 5 min and the next 10 min, and time spent in the center area (20 cm by 20 cm; about 25% of the total area) were recorded and analyzed with EthoVision XT software (Noldus).

#### *Acoustic startle response*, *sensory habituation*, *and prepulse inhibition tests*

The startle reflex measurement system (Kinder Scientific) was used for assessing acoustic startle responses, habituation of the acoustic startle response, and prepulse inhibition. A test session began by placing a mouse in a chamber, where it was left undisturbed for 5 min. The startle response was recorded for 250 ms starting with the onset of a startle stimulus. The background noise level in the chamber was 60 dB. The peak startle amplitude during the 250-ms sampling window was recorded as a measure of the startle response. The average intertrial interval was 15 s (range, 10 to 20 s). The acoustic startle response test was done on day 1. The intensities of startle stimuli were 60, 70, 80, 90, 100, 110, and 120 dB. The habituation test of the acoustic startle response was done on day 2. The startle stimuli (110 dB) were applied 50 times. Normalized startle responses were determined by dividing each response by the mean of the first two responses. For the analysis, 10 normalized startle responses were binned. For the quantitative assessment of the habituation, the average of the last 20 startle responses was divided by the average of the first two responses. The prepulse inhibition test was done on day 3. The prepulse sound was presented 100 ms before the startle stimulus (110 dB), and its intensity was 70, 74, 78, or 82 dB. Each trial was presented in pseudorandom order. The following formula was used to calculate the % prepulse inhibition of the startle response: 100 − [100 × (startle response on prepulse trials/startle response on 110 dB startle trials)].

#### Elevated plus maze test

The elevated plus maze consisted of two open arms and two closed arms of the same size (35 cm by 6 cm) extending from the central area (6 cm by 6 cm). The maze was elevated 75 cm from the ground and illuminated at 3 lux. Mice were placed in the central square of the maze facing one of the open arms and allowed to move for 10 min. Times spent in the open arm, closed arm, and center area were recorded and analyzed with EthoVision XT software.

#### Light-dark transition test

The light-dark box consisted of two compartments: a white (light) box without a lid (500 lux) and a black (dark) box with a lid (both 25 cm by 25 cm by 25 cm). The two boxes were separated by a vertical sliding door that remained open (4 cm by 6 cm). Mice were placed in the black box and allowed to move between boxes for 10 min. Times spent in the light box and dark box and the transition numbers between boxes were recorded and analyzed with EthoVision XT software.

#### Behavioral experiments with a touchscreen operant system

The test was conducted in a touchscreen operant chamber equipped with an infrared touchscreen, a black mask with two windows, a liquid dipper, and a reward tray (Lafayette Instrument Company). Mice could access 10% condensed milk (Borden Sugar Condensed Eagle Brand Milk) as a reward from the reward tray. The operant chamber is controlled using ABET II (Lafayette Instrument Company) and Whisker (Lafayette Instrument Company) software. Throughout the testing, the mice’s body weights were maintained at 85% of free-feeding weight by restriction feeding. During restriction feeding, mice could access around 2 g of regular mouse chow per day.

#### Pretraining

Pre-training involved three stages. Mice were required to meet a set criterion for each stage before proceeding to the next stage. In stage 1 (acclimation), mice were placed in the chamber for 60 min and exposed to 200 μl of condensed milk in the reward tray. Once the mice had consumed all the milk in 60 min, they proceeded to the next stage. In stage 2 (reward acquisition training), the mice were trained to collect a milk reward (20 μl each time) from the reward tray after the following cues: a high-pitched tone and illumination of the tray light. After the reward was collected, the tray illumination was turned off, and a 30-s intertrial interval was initiated before the delivery of the next reward. Mice that successfully completed 40 trials in 60 min proceeded to the next stage. In stage 3 (stimulus touch training), mice were trained to touch visual stimuli that were randomly presented in one of the two spatial locations on the screen. Forty different shapes were used for the visual stimuli, none of which were used for the visual discrimination or reversal learning task. If mice touch the blank screen, then no reward is given. Touching the visual stimulus results in its disappearance, accompanied by the delivery of the reward, a high-pitched tone, and tray illumination. Reward collection turned off the tray illumination and initiated the next trial. Mice that successfully completed 60 trials in 60 min were considered to have met the criterion in stage 3. The days to the criterion were quantified for stages 1 to 3. Touch latency (time from visual stimulus presentation to touch response) for stage 3 and reward latency (time from reward delivery to reward retrieval) for stages 2 and 3 were measured. The median of reward latency and touch latency for total trials were calculated using the ABET II analysis program, and the average of those values for each mouse group was plotted on the graph.

#### Visual-guided associative learning task

In the visual-guided associative learning task (*see* Fig. 7, A and B), mice were trained to touch the correct visual stimulus from two different visual stimuli that were randomly presented on the screen. Images of a fan and marbles were used for the visual stimuli. The correct visual stimulus was counterbalanced in half of the mouse groups. Touching the correct visual stimulus (correct response) results in its disappearance, accompanied by the delivery of the reward, a high-pitched tone, and tray illumination. Reward collection turned off the tray illumination and initiated the next trial. Touching the incorrect visual stimulus (incorrect response) results in a 5-s timeout accompanied by a low-pitched tone and the illumination of a house light. After the timeout, the tray light illuminated so that the mouse could nose-poke the tray to initiate the next trial. One session was conducted per day and lasted 60 min, with a maximum of 60 trials. Mice that successfully achieved an average correct response rate of at least 80% in 60 trials over two consecutive days were considered to have met the criterion in the visual-guided associative learning task. At least four sessions were completed, regardless of whether this criterion was met. Percentage of correct responses (correct responses/60 trials), total trials required to criterion, total errors made to criterion, and correct response latency (time from visual stimulus presentation to correct touch response) were measured. In Fig. 7 (C and D) (correct response) and fig. S15 (G and H) (correct response latency), because mice that met the criterion earlier than day 9 were immediately moved to the reversal learning task, the values when the mice met the criterion were repeatedly plotted until day 9 in the graphs. The median of correct response latency was calculated using the ABET II analysis program.

#### Reversal learning task

In the reversal learning task, the order of the task was the same as in the visual-guided associative learning task, but for each mouse, the correct visual stimulus was switched to the other visual stimulus. One session was conducted per day and lasted 60 min, with a maximum of 60 trials. However, because a few mice had a large decrease in trial number per day in the early stages of reversal learning, we combined trials from the following day to adjust the number of trials to 60 per session. Mice that successfully achieved an average correct response rate of at least 80% in 60 trials over two consecutive days were considered to have met the criterion for reversal learning. Regardless of whether this criterion was met, at least 13 sessions were completed. In addition to the analysis in the visual-guided associative learning task, total errors in the early (correct response 0 to 40%), mid (correct response, 0 to 60%), and late (correct response, 60% criterion) phases were determined. Perseverative errors for sessions 1 to 5 were also measured, where four consecutive incorrect responses were counted as one perseverative error.

### Statistics and reproducibility

Statistical analyses were performed using GraphPad Prism software. The statistical tests performed were two-tailed Student’s *t* test, Mann-Whitney *U* test, one-way analysis of variance (ANOVA), followed by the Šidák test, two-way ANOVA followed by the Šidák test, and two-way repeated-measures ANOVA, followed by the Šidák test as indicated in the results and figure legends. All data are expressed as means ± SEM. Sample sizes (*n*) are indicated in the figure legends. Significance was set as *P < 0.05, **P < 0.01, ***P < 0.001, or ****P < 0.0001 for all data. The sample sizes were not predetermined by statistical methods; however, our sample sizes were similar to those reported in previous publications in the field (*10*, *61*, *63*). All experiments were reproduced across at least two independent cohorts of mice. Steps in the experiments were randomized to minimize the effects of confounding variables, including how mice were chosen for experiments, order of treatments, etc. Imaging was done in the same fashion among conditions. For quantification, cells and fields from brain sections were chosen randomly from the region of interest. No data points were excluded from any experiments.
