# Supplementary material for: δ2-Protocadherins organize parallel indirect basal ganglia circuits
Source: Sci Adv. 2026 Aug 21;12(34):eaec8746. doi: 10.1126/sciadv.aec8746 (PMC13496169; doi:10.1126/sciadv.aec8746)
Supplement: Supplementary file 1 [file sciadv.aec8746_sm.pdf]

Science Advances
AAAS

Supplementary Materials for

δ2-Protocadherins organize parallel indirect basal ganglia circuits

Naosuke Hoshina *et al.*

Corresponding author: Hisashi Umemori, hisashi.umemori@childrens.harvard.edu;
Naosuke Hoshina, naosuke.hoshina@aoni.waseda.jp

Sci. Adv. **12**, eaec8746 (2026)
DOI: 10.1126/sciadv.aec8746

This PDF file includes:

Figs. S1 to S16

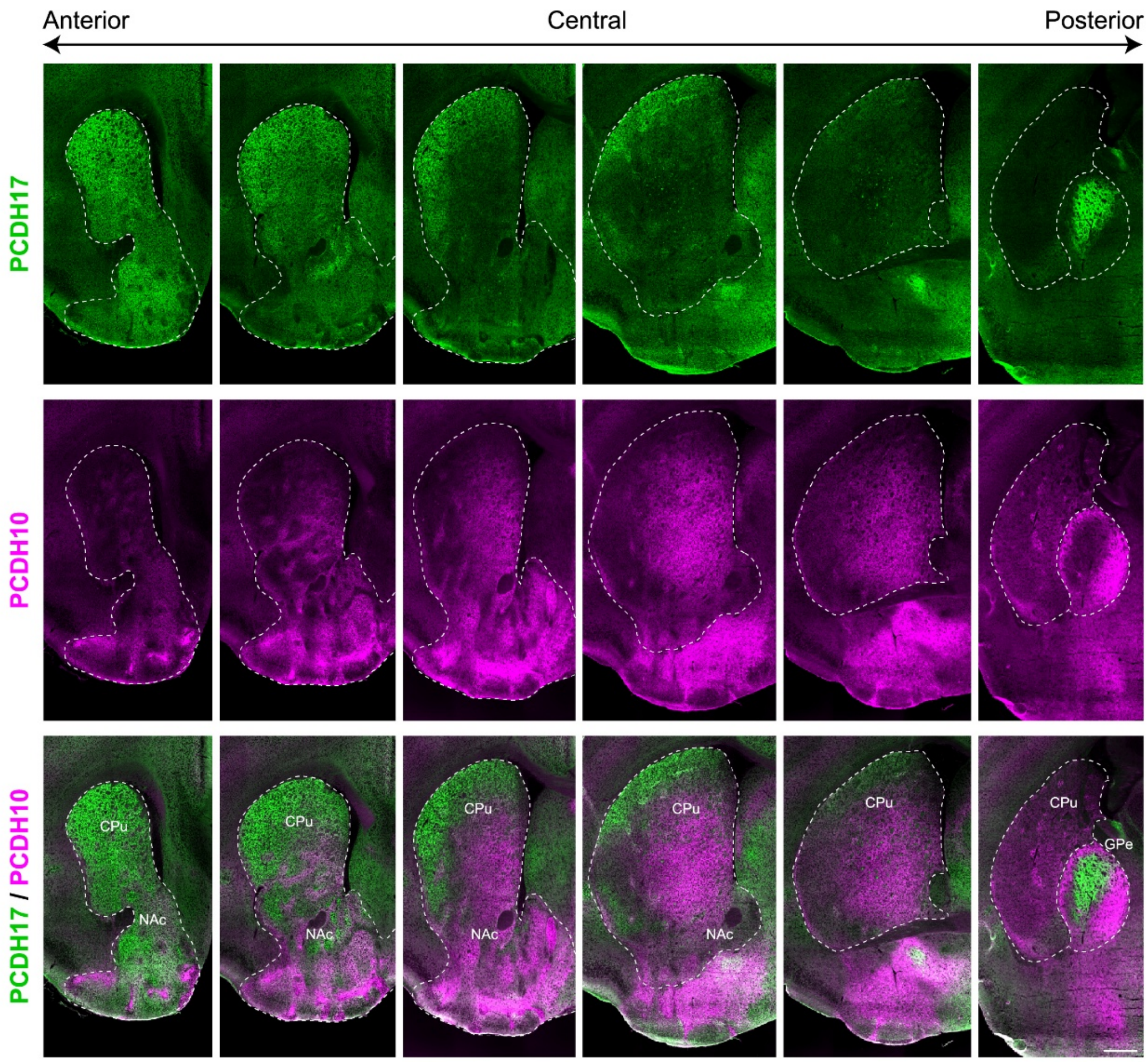

Fig. S1. Complementary expression patterns of PCDH17 and PCDH10 proteins in the striatum.
Double staining for PCDH17 (green) and PCDH10 (magenta) of coronal striatal sections from P11 mice. PCDH17 and PCDH10 proteins show complementary expression patterns in the striatum, including the caudate putamen (CPu) and nucleus accumbens (NAc). The scale bar represents 0.5 mm.

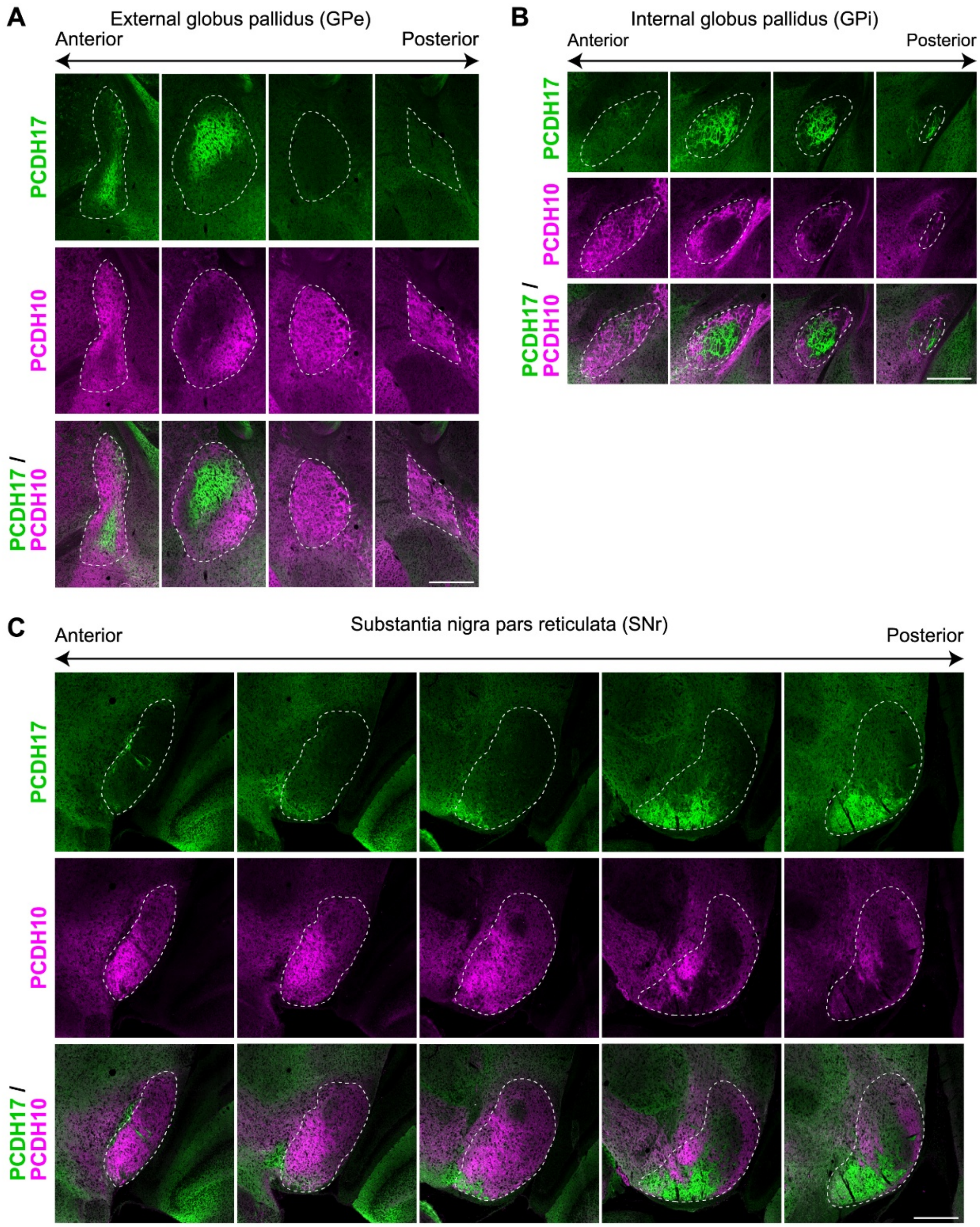

Fig. S2. Complementary expression patterns of PCDH17 and PCDH10 proteins in the GPe, GPi, and SNr.

Double staining for PCDH17 (green) and PCDH10 (magenta) of coronal BG sections from P11 mice. PCDH17 and PCDH10 proteins show complementary expression patterns in the GPe (**A**), GPi (**B**), and SNr (**C**). The scale bars represent 0.5 mm.

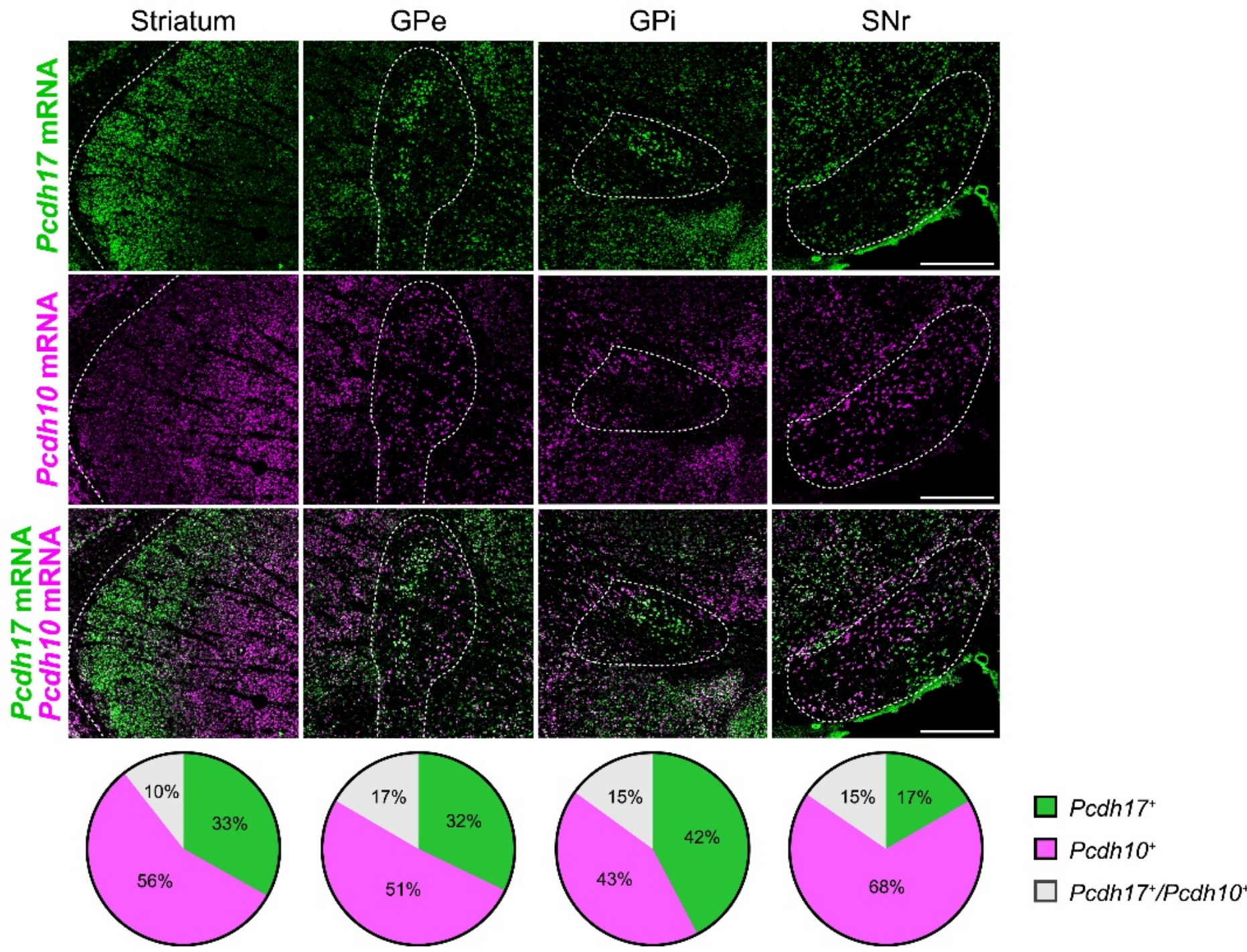

Fig. S3. Complementary expression patterns of *Pcdh17* and *Pcdh10* mRNA in the BG. Double fluorescent *in situ* hybridization for *Pcdh17* (green) and *Pcdh10* (magenta) mRNAs in a sagittal BG section from a P10 mouse. *Pcdh17* mRNA is preferentially expressed in the anterior striatum and inner GP, while *Pcdh10* mRNA is preferentially expressed in the posterior striatum and outer GP. Reproduced from (*10*). Pie charts illustrate the proportions of cells expressing *Pcdh17* only, *Pcdh10* only, and both *Pcdhs* in each basal ganglia nucleus. While most cells only express *Pcdh17* or *Pcdh10*, a subset of cells co-expresses both *Pcdhs*. Number of cells analyzed: 1168 (Striatum), 709 (GPe), 187 (GPi), and 234 (SNr). The scale bars represent 0.5 mm.

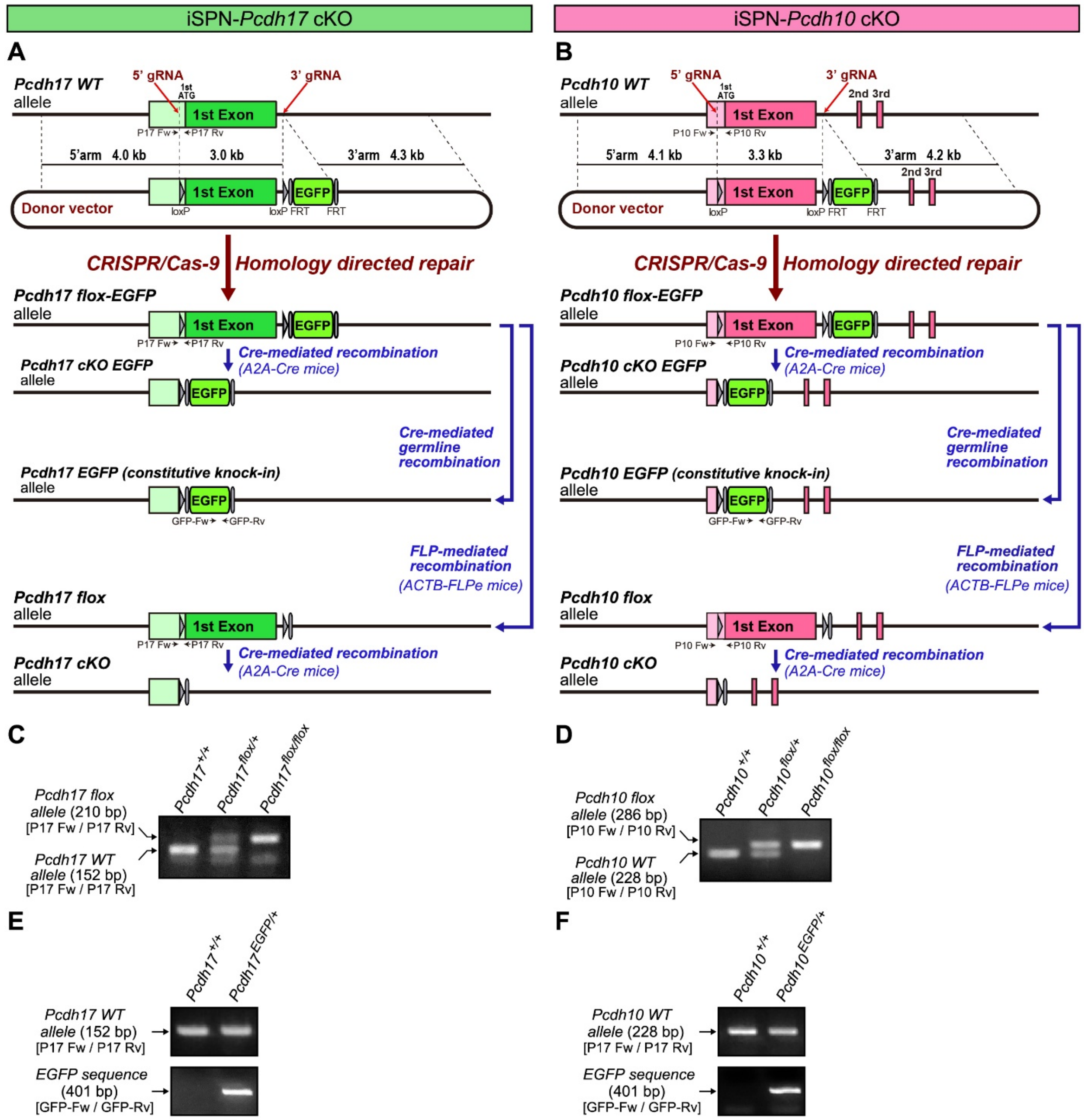

Fig. S4. Generation of iSPN-specific cKO mice for *Pcdh17* and *Pcdh10*.
(**A** and **B**) Schematic illustrations of the strategy for generating conditional knockout (cKO) mice for *Pcdh17* (**A**) and *Pcdh10* (**B**) using the CRISPR/Cas9 system. For each *Pcdh17* and *Pcdh10* gene: the WT allele, donor vector, flox-EGFP allele, cKO-EGFP allele, EGFP knock-in allele, flox allele, and cKO allele are represented. The donor vector contained the loxP-flanked 1st exon and the FRT-flanked EGFP cassette, along with ~4 kb of 5' and 3' homology arms. Two gRNAs, 5' and 3', are designed to remove the 1st exon from the WT allele and replace it with the sequence from the donor vector by homology-directed repair, which generates the *Pcdh*-flox-EGFP allele. The iSPN-specific *Pcdh*-cKO-EGFP allele was produced by mating *Pcdh*-flox-EGFP mice with *A2A-Cre* mice. In *Pcdh*-cKO-EGFP mice, the *Pcdh* promoter drives EGFP

expression in Cre-positive iSPNs. *Pcdh*-EGFP-knock-in mice were generated via Cre-mediated germline recombination. The *Pcdh*-flox-EGFP allele was converted to the *Pcdh*-flox allele via the FRT-FLP recombination system by crossing with *ACTB-FLPe* mice. iSPN-specific *Pcdh*-cKO mice were generated by mating *Pcdh*-flox mice with *A2A-Cre* mice. P17 Fw and P17 Rv are the *Pcdh17* flox mice genotyping primers. P10 Fw and P10 Rv are the *Pcdh10* flox mice genotyping primers. GFP Fw and GFP Rv are genotyping primers for the EGFP cassette.
(**C**) Genotyping PCR results for $Pcdh17^{+/+}$, $Pcdh17^{flox/+}$, and $Pcdh17^{flox/flox}$ mice.
(**D**) Genotyping PCR results for $Pcdh10^{+/+}$, $Pcdh10^{flox/+}$, and $Pcdh10^{flox/flox}$ mice.
(**E**) Genotyping PCR results for $Pcdh17^{+/+}$ and $Pcdh17^{EGFP/+}$ mice.
(**F**) Genotyping PCR results for $Pcdh10^{+/+}$ and $Pcdh10^{EGFP/+}$ mice.

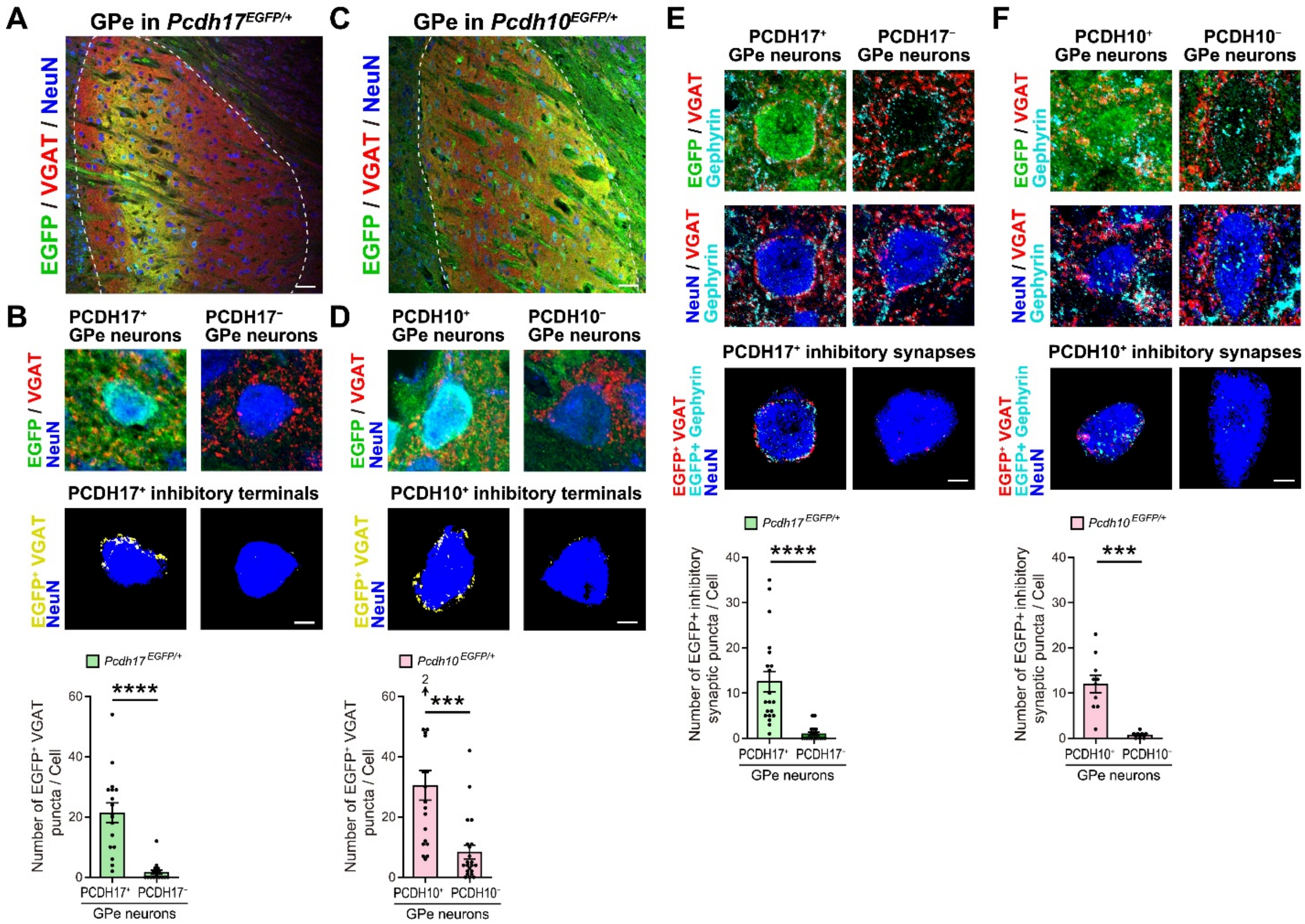

Fig. S5. PCDH17- and PCDH10-positive inhibitory terminals synapse on GPe neurons with matching PCDH expression.

(**A**) Sagittal sections of the GPe from P11 *Pcdh17*$^{EGFP/+}$ mice were immunostained for EGFP (green), VGAT (red), and NeuN (blue). EGFP labels both PCDH17^{+} GPe neurons and PCDH17^{+} inhibitory terminals.

(**B**) PCDH17^{+} inhibitory terminals targeting the cell body of PCDH17^{+} GPe neurons (EGFP^{+}/NeuN^{+}) and PCDH17$^{–}$ GPe neurons (EGFP$^{–}$/NeuN^{+}). PCDH17^{+} inhibitory terminals (EGFP^{+}/VGAT^{+} yellow puncta) were extracted by overlaying binarized EGFP and VGAT signals. NeuN signals were also binarized to identify cell bodies (blue). Quantification revealed that PCDH17^{+} inhibitory terminals selectively innervate PCDH17^{+} GPe neurons ($P < 0.0001$). n = 17 cells.

(**C**) Sagittal sections of the GPe from P13 *Pcdh10*$^{EGFP/+}$ mice were immunostained for EGFP (green), VGAT (red), and NeuN (blue). EGFP labels both PCDH10^{+} GPe neurons and PCDH10^{+} inhibitory terminals.

(**D**) PCDH10^{+} inhibitory terminals targeting the cell bodies of PCDH10^{+} GPe neurons (EGFP^{+}/NeuN^{+}) and PCDH10$^{–}$ GPe neurons (EGFP$^{–}$/NeuN^{+}). PCDH10^{+} inhibitory terminals (EGFP^{+}/VGAT^{+} yellow puncta) were extracted by overlaying binarized EGFP and VGAT signals. NeuN signals were also binarized to identify cell bodies (blue). Quantification revealed that PCDH10^{+} inhibitory terminals preferentially innervate PCDH10^{+} GPe neurons ($P = 0.0001$). n = 19–22 cells.

(**E**) PCDH17^{+} inhibitory synapses on the cell body of PCDH17^{+} GPe neurons (EGFP^{+}/NeuN^{+}) and PCDH17^{-} GPe neurons (EGFP^{-}/NeuN^{+}). PCDH17^{+} inhibitory synapses (EGFP^{+}/VGAT^{+} red puncta [pre] apposed to EGFP^{+}/Gephyrin^{+} cyan puncta [post]) were extracted by overlaying binarized EGFP, VGAT, and Gephyrin signals. NeuN signals were also binarized to identify cell bodies (blue). Quantification of PCDH17^{+} inhibitory synapses on GPe neurons revealed that PCDH17^{+} inhibitory synapses selectively form on PCDH17^{+} GPe neurons ($P < 0.0001$). n = 19–20 cells.
(**F**) PCDH10^{+} inhibitory synapses on the cell body of PCDH10^{+} GPe neurons (EGFP^{+}/NeuN^{+}) and PCDH10^{-} GPe neurons (EGFP^{-}/NeuN^{+}). PCDH10^{+} inhibitory synapses (EGFP^{+}/VGAT^{+} red puncta [pre] apposed to EGFP^{+}/Gephyrin^{+} cyan puncta [post]) were extracted by overlaying binarized EGFP, VGAT, and Gephyrin signals. NeuN signals were also binarized to identify cell bodies (blue). Quantification of PCDH10^{+} inhibitory synapses on GPe neurons revealed that PCDH10^{+} inhibitory synapses selectively form on PCDH10^{+} GPe neurons ($P = 0.0001$). n = 8–10 cells.
The scale bars represent 50 µm (**A**, **C**) and 5 µm (**B**, **D**, **E** and **F**). Data are mean ± SEM. ***$P < 0.001$, ****$P < 0.0001$ by Student's t-test.

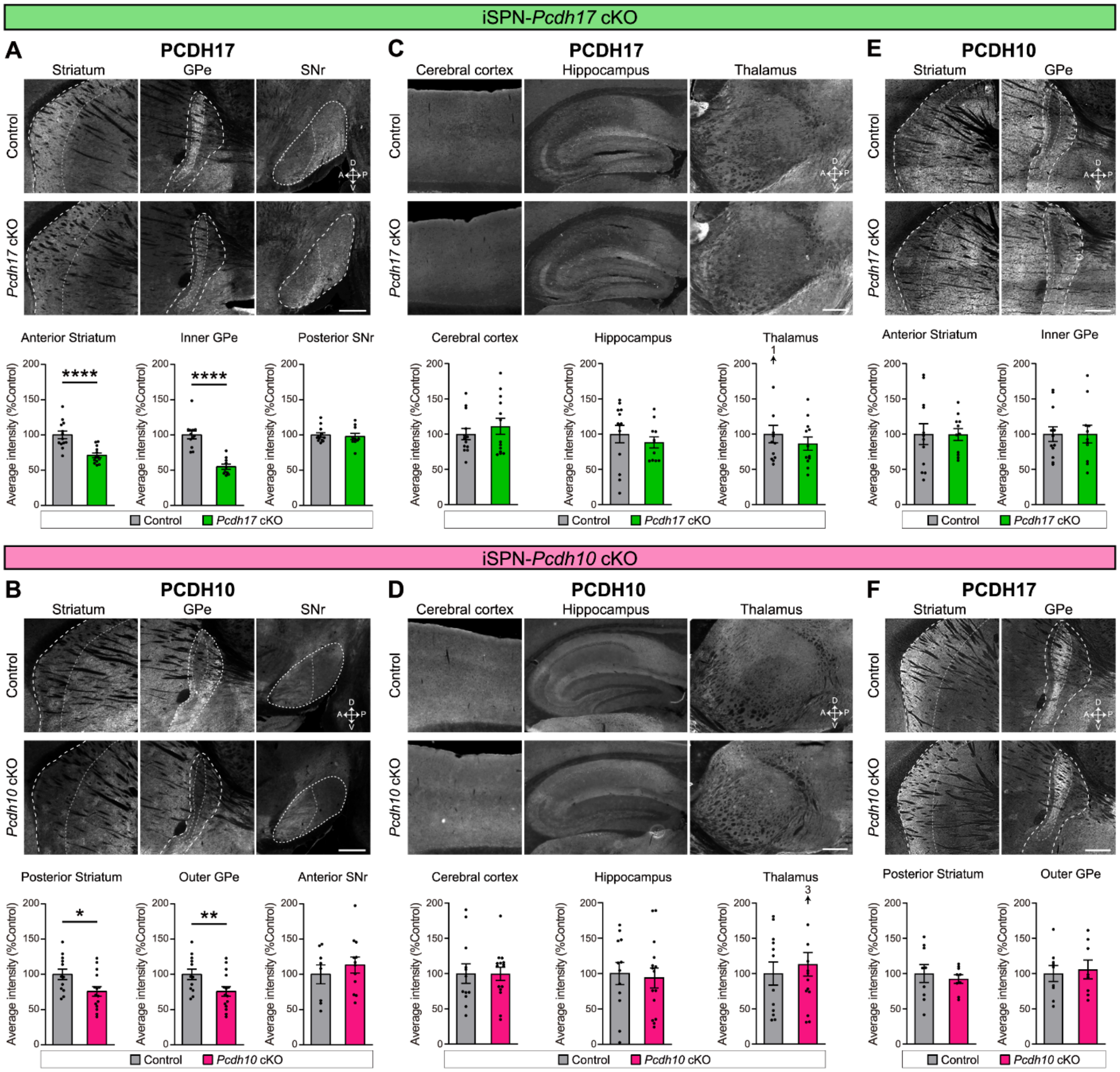

Fig. S6. Conditional knockout of PCDH17 or PCDH10 in indirect BG circuits.

(**A**) (Top) Immunostaining for PCDH17 of BG sections from adult control (*A2A-Cre;Pcdh17*$^{+/+}$) and *Pcdh17* cKO (*A2A-Cre;Pcdh17*$^{flox/flox}$) mice. (Bottom) Average intensities of PCDH17 staining signals in the anterior striatum, inner GPe, and posterior SNr were quantified. *Pcdh17* cKO mice show reduced PCDH17 staining intensity in the striatum ($P < 0.0001$) and GPe ($P < 0.0001$), but not in the SNr ($P = 0.6854$), compared to control mice. n = 10–13 fields from 3 mice per genotype.

(**B**) (Top) Immunostaining for PCDH10 of BG sections from adult control (*A2A-Cre;Pcdh10*$^{+/+}$) and *Pcdh10* cKO (*A2A-Cre;Pcdh10*$^{flox/flox}$) mice. (Bottom) Average intensities of PCDH10 staining signals in the posterior striatum, outer GPe, and anterior SNr were quantified. *Pcdh10* cKO mice show reduced PCDH10 staining intensity in the striatum ($P = 0.0240$) and GPe ($P = 0.0050$), but not in the SNr ($P = 0.4590$), compared to control mice. n = 8–15 fields from 4–5 mice per genotype.

(**C**) (Top) Immunostaining for PCDH17 of BG sections from adult control (*A2A-Cre;Pcdh17*$^{+/+}$) and *Pcdh17* cKO (*A2A-Cre;Pcdh17*$^{flox/flox}$) mice. (Bottom) Average intensities of PCDH17 staining signals in the cerebral cortex, hippocampus, and thalamus were quantified. *Pcdh17* cKO mice show similar levels of PCDH17 staining intensity in these areas compared to control mice (cerebral cortex; P = 0.4325, hippocampus; P = 0.4456, thalamus; P = 0.3928). n = 11–13 fields from 3 mice per genotype.
(**D**) (Top) Immunostaining for PCDH10 of BG sections from adult control (*A2A-Cre;Pcdh10*$^{+/+}$) and *Pcdh10* cKO (*A2A-Cre;Pcdh10*$^{flox/flox}$) mice. (Bottom) Average intensities of PCDH10 staining signals in the cerebral cortex, hippocampus, and thalamus were quantified. *Pcdh10* cKO mice show similar levels of PCDH10 staining intensity in these areas compared to control mice (cerebral cortex; P = 0.9757, hippocampus; P = 0.7835, thalamus; P = 0.5860). n = 12–15 fields from 4–5 mice per genotype.
(**E**) (Top) Immunostaining for PCDH10 of BG sections from 3-week-old control and *Pcdh17* cKO mice. (Bottom) Average intensities of PCDH10 staining signals in the anterior striatum and inner GPe were quantified. *Pcdh17* cKO mice show similar levels of PCDH10 staining intensity in the striatum (P = 0.9751) and GPe (P = 0.9945) compared to control mice. n = 11–12 fields from 6 mice per genotype.
(**F**) (Top) Immunostaining for PCDH17 of BG sections from 3-week-old control and *Pcdh10* cKO mice. (Bottom) Average intensities of PCDH17 staining signals in the posterior striatum and outer GPe were quantified. *Pcdh10* cKO mice show similar levels of PCDH17 staining intensity in the striatum (P = 0.5949) and GPe (P = 0.7452) compared to control mice. n = 8–9 fields from 5 mice per genotype.
Longer dashed lines outline the striatum, GPe, and SNr, and shorter dashed lines indicate subregional borders (anterior/posterior striatum, inner/outer GPe, and anterior/posterior SNr) (**A**, **B**, **E**, and **F**). The scale bars represent 0.5 mm. Data are mean ± SEM. *P < 0.05, **P < 0.01, ****P < 0.0001 by Student's *t*-test. GPe, external globus pallidus; GPi, internal globus pallidus; SNr, substantia nigra pars reticulata.

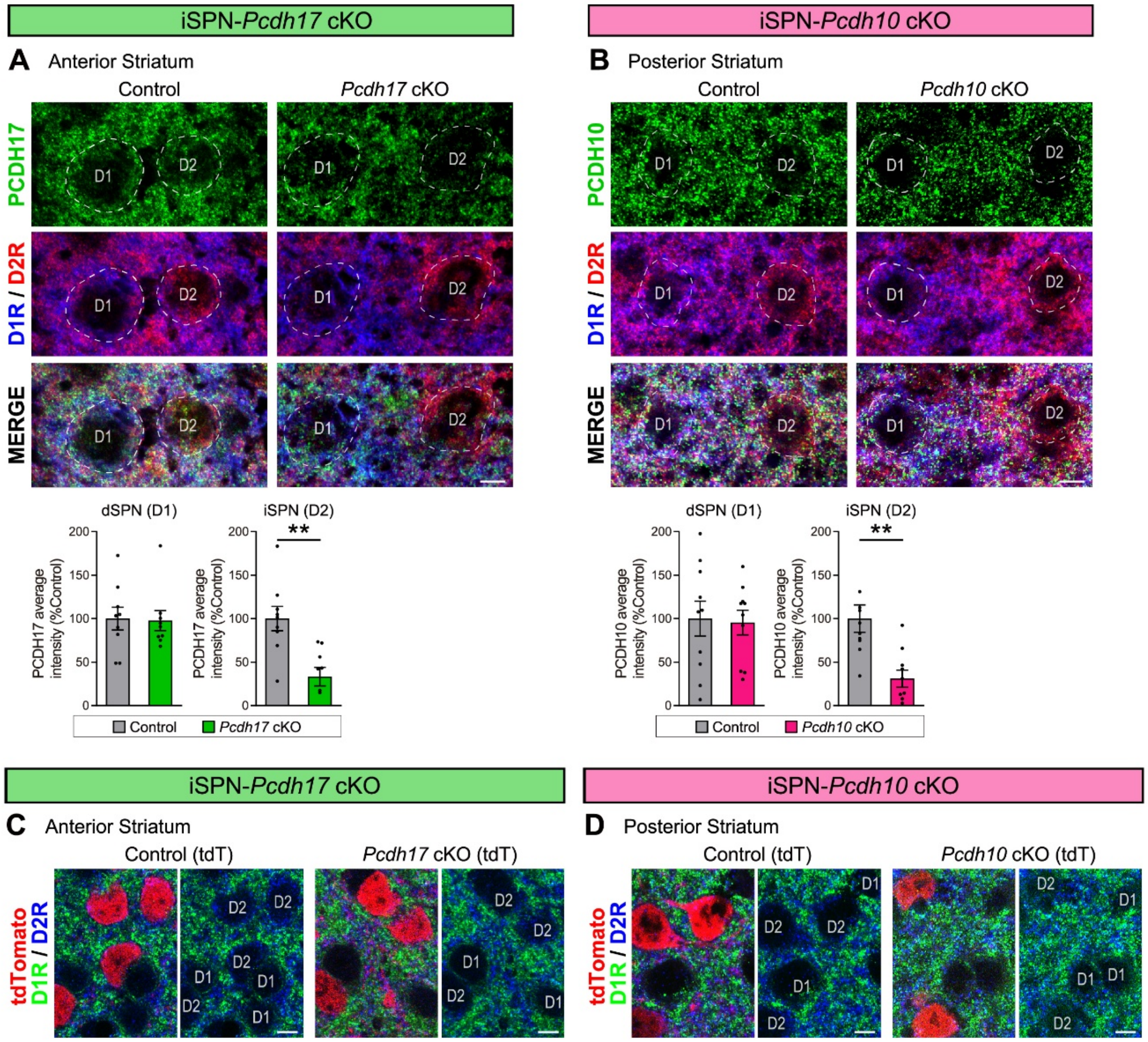

Fig. S7. iSPN-specific knockout of PCDH17 or PCDH10 in the striatum.

(**A**) Triple staining for PCDH17 (green), D1R (blue), and D2R (red) in the anterior striatum of 3-week-old control and *Pcdh17* cKO mice. Average intensities of PCDH17 staining signals on the cell bodies of dSPNs (D1R-positive) and iSPNs (D2R-positive) were quantified. *Pcdh17* cKO mice show significantly reduced PCDH17 staining signals in iSPNs but not in dSPNs compared to control mice (P = 0.8954 for dSPN, P = 0.0016 for iSPN). n = 9 cells for iSPNs and dSPNs from 5 mice per genotype.

(**B**) Triple staining for PCDH10 (green), D1R (blue), and D2R (red) in the posterior striatum of 3-week-old control and *Pcdh10* cKO mice. Average intensities of PCDH10 staining signals on the cell bodies of dSPNs and iSPNs were quantified. *Pcdh10* cKO mice show significantly reduced PCDH10 staining signals in iSPNs but not in dSPNs compared to control mice (P = 0.8550 for dSPN, P = 0.0015 for iSPN). n = 10 cells for iSPNs and dSPNs from 5 mice per genotype.

(**C** and **D**) iSPN cells were labeled with tdTomato by crossing *A2A-Cre* mice with *Rosa*$^{STOP\text{-}tdT}$ mice. Staining for D1R (green) and D2R (blue) in the anterior striatum of 3-week-old control

(tdT) and *Pcdh17* cKO (tdT) mice (**C**) and in the posterior striatum of 3-week-old control (tdT) and *Pcdh10* cKO (tdT) mice (**D**). tdTomato-positive iSPN cells are D2R-positive across all genotypes.
Dashed lines outline the cell bodies of iSPNs and dSPNs (**A** and **B**). The scale bars represent 5 μm. Data are mean ± SEM. $^{**}P < 0.01$ by Student's *t*-test.

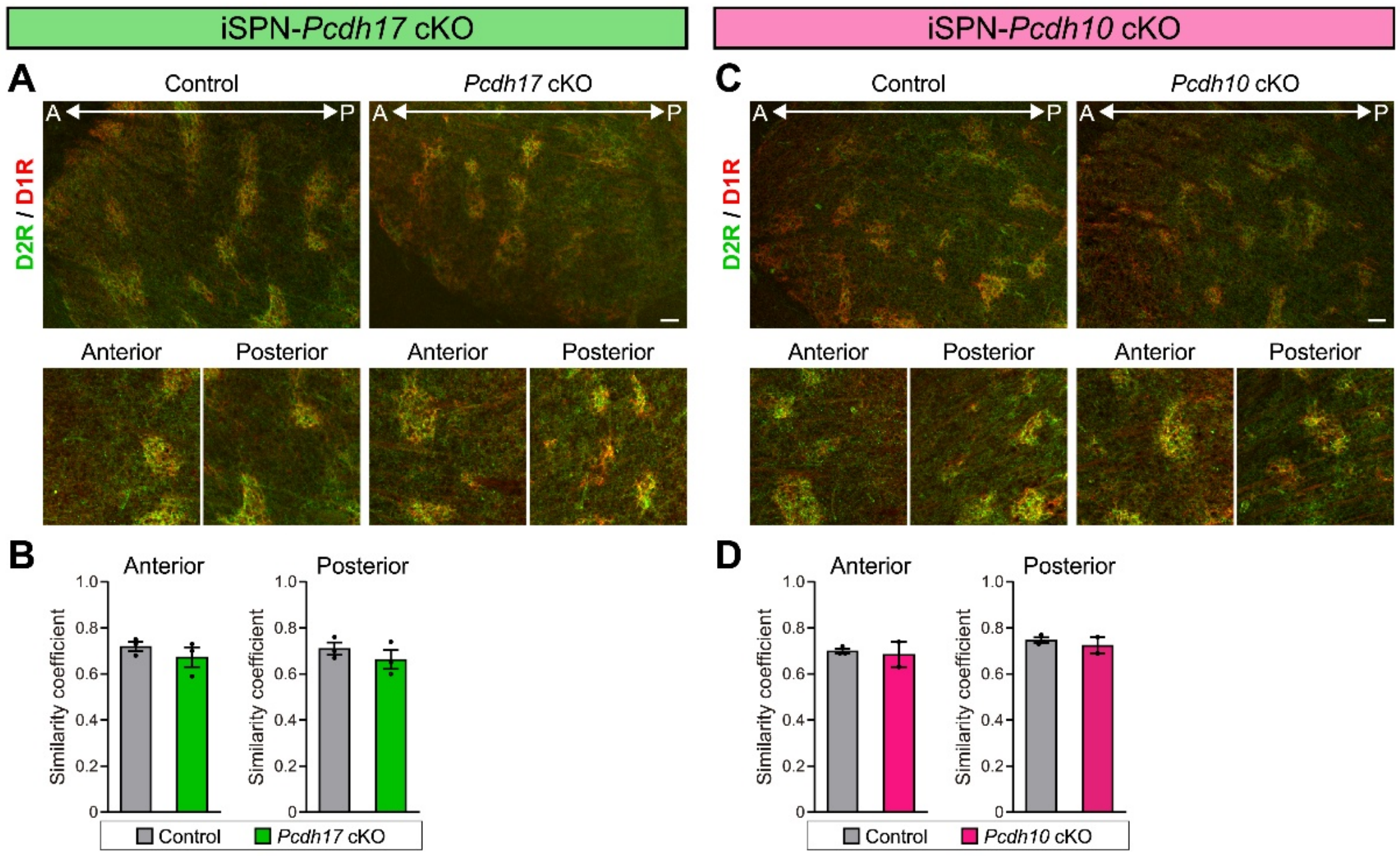

Fig. S8. Normal D1R and D2R distribution in the striatum of iSPN-*Pcdh* cKO mice at postnatal day 0.

(**A**) Double staining for D2R (green; iSPNs) and D1R (red; dSPNs) of anterior and posterior striatal sections from P0 *Pcdh17* cKO and control mice. (**B**) Quantification of the similarity coefficient in the anterior ($P = 0.3804$) and posterior ($P = 0.3928$) striatum. $n = 3$ fields from 3 mice per genotype. (**C**) Double staining for D1R and D2R of anterior and posterior striatal sections from P0 *Pcdh10* cKO and control mice. (**D**) Quantification of the similarity coefficient in the anterior ($P = 0.8220$) and posterior ($P = 0.4439$) striatum. $n = 3$ fields from 3 mice per genotype.

The scale bars represent 50 µm. Data are mean ± SEM. *$P < 0.05$ by Student's *t*-test.

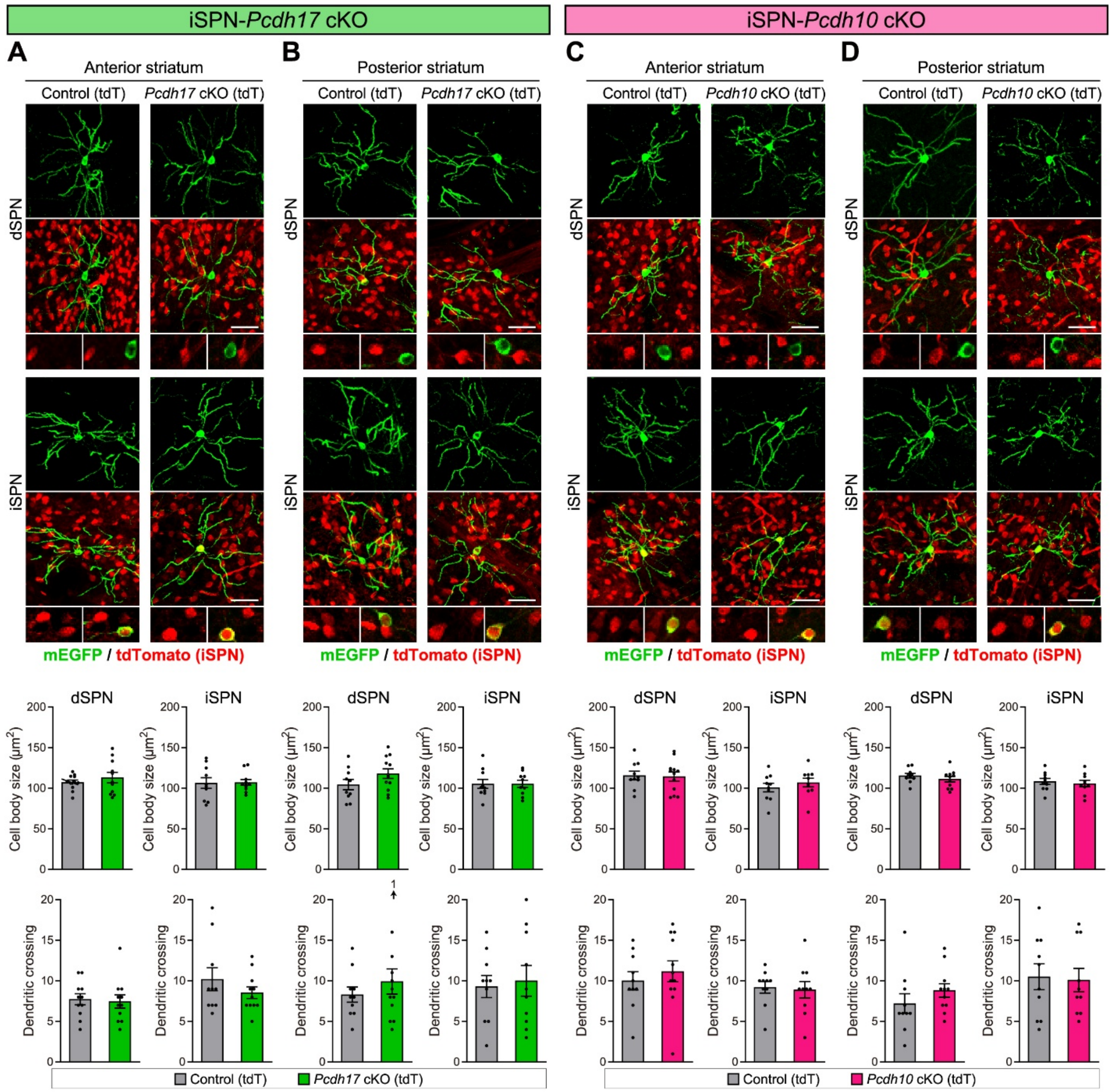

Fig. S9. The morphology of dSPNs and iSPNs is normal in iSPN-*Pcdh* cKO mice.
(**A** to **D**) The morphology of dSPNs and iSPNs, assessed by labeling with membrane-EGFP (mEGFP), in the anterior and posterior striatum of adult *Pcdh* cKO (tdT) and control mice (tdT). tdTomato$^+$ cells are iSPNs. Single-plane images of the cell bodies are magnified in the bottom panels to demonstrate mEGFP$^+$/tdTomato$^-$ cells (dSPNs) and mEGFP$^+$/tdTomato$^+$ cells (iSPNs). (**A** and **B**) The cell body size and number of dendritic crossings of dSPNs and iSPNs in both the anterior (**A**; cell body size; P = 0.4130 for dSPN, P = 0.9166 for iSPN, dendritic crossings; P = 0.8006 for dSPN, P = 0.2964 for iSPN) and posterior (**B**; cell body size; P = 0.1269 for dSPN, P = 0.9879 for iSPN, dendritic crossings; P = 0.4068 for dSPN, P = 0.7667 for iSPN) striatum are similar between control (tdT) and *Pcdh17* cKO (tdT) mice. n = 10-12 cells for iSPNs and dSPNs in the anterior and posterior striatum from 3 mice per genotype. (**C** and **D**) The cell body size and number of dendritic crossings of dSPNs and iSPNs in both the anterior (**C**; cell body size; P = 0.8712 for dSPN, P = 0.4308 for iSPN, dendritic crossings; P = 0.5123 for dSPN, P = 0.8116

for iSPN) and posterior (**D**; cell body size; $P = 0.3249$ for dSPN, $P = 0.6316$ for iSPN, dendritic crossings; $P = 0.2761$ for dSPN, $P = 0.8551$ for iSPN) striatum are similar between control (tdT) and *Pcdh10* cKO (tdT) mice. $n = 10$–12 cells for iSPNs and dSPNs in the anterior and posterior striatum from 3 mice per genotype.
The scale bars represent 50 µm. Data are mean ± SEM. Student's *t*-test.

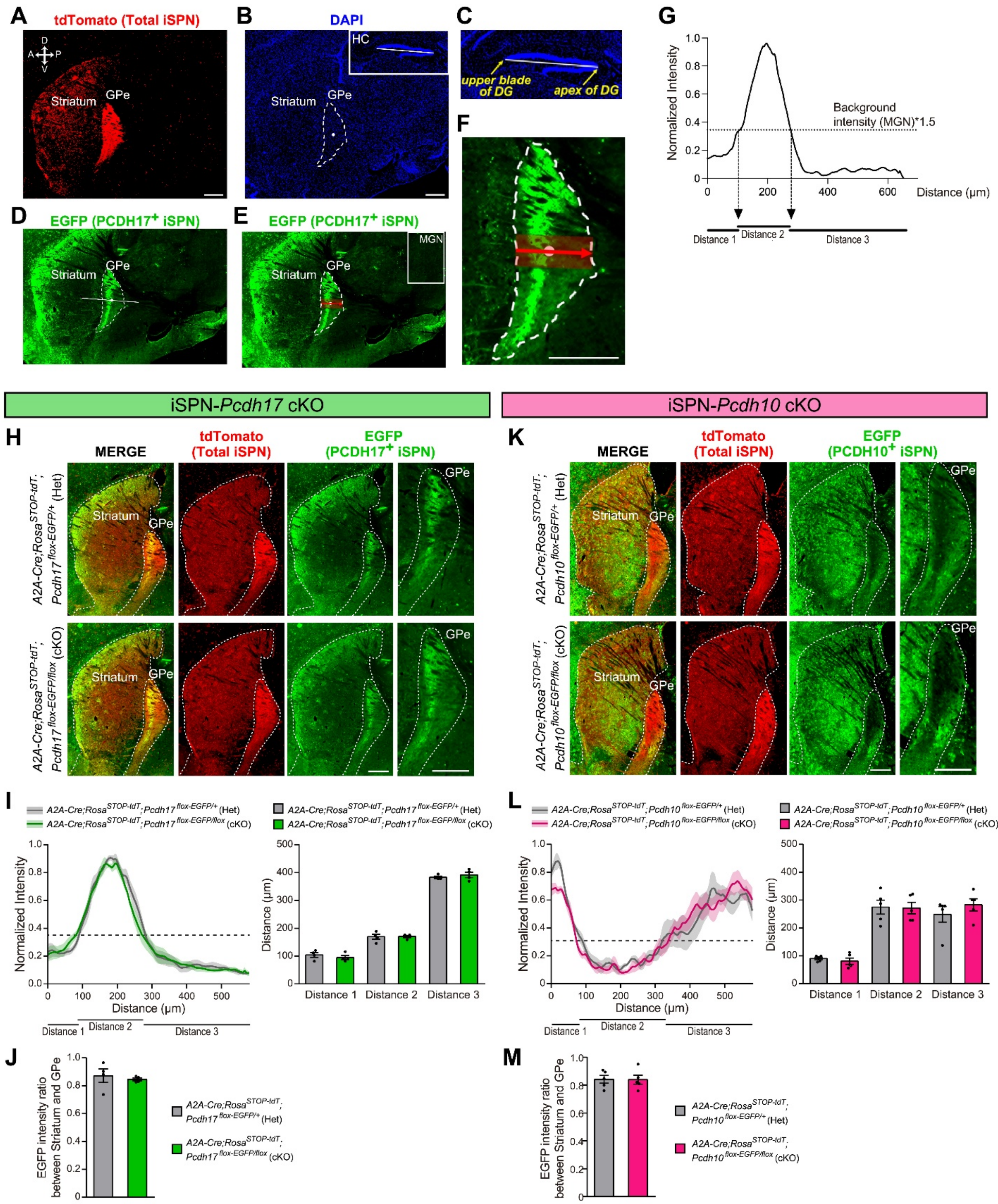

Fig. S10. Normal iSPN axonal targeting in iSPN-*Pcdh* cKO mice.
(**A** to **G**) Evaluation of iSPN axon targeting in the GPe. (**A**) The tdTomato signal labels all the iSPNs within the striatum and their axonal projections to the GPe in red. The outline of the tdTomato signal in the GPe (dashed, white line) was used to determine the geometrical center of the GPe (white dot within dashed outline). (**B** and **C**) DAPI staining was used to construct a line

in the hippocampus (HC, white box in **B**). The line connects the apex of the dentate gyrus (DG) and the anterior end of the upper blade of the DG (**C**). A parallel to this line was then drawn through the geometrical center of the GPe (**B**). (**D**) Immunostaining for EGFP (green) visualizes the area of iSPN axonal projection within the GPe as a result of EGFP expression under the *Pcdh* promoter in iSPNs. (**E** and **F**) The line through the geometrical center was used to perform a 50-pixel wide line scan crossing the GPe from the anterior to the posterior side (the red arrow indicates the orientation of the line scan; width of the line scan is represented by a red box). (**G**) The resulting line scan (the highest intensity in the scan was set as 1 and the remaining values were normalized accordingly) was evaluated. The background intensity was calculated from the mean intensity in the medial geniculate nucleus (MGN, white box in **E**) and introduced in the line scan (dotted line). This resulted in two crossing points with the line scan, which marked the borders of axonal innervation. We then calculated the distances between the beginning of the line scan and the first border of axonal innervation (Distance 1), the first and second borders of axonal innervation (Distance 2), and the second border of axonal innervation and the end of the line scan (Distance 3).
(**H** to **M**) Normal iSPN axonal targeting in iSPN-*Pcdh* cKO (*A2A-Cre;Rosa*$^{STOP\text{-}tdT}$*;Pcdh*$^{flox\text{-}EGFP/flox}$) mice. (**H**) Immunostaining for tdTomato (red: labels all iSPNs) and EGFP (green: labels PCDH17-expressing iSPNs) of BG sections from adult *A2A-Cre;Rosa*$^{STOP\text{-}tdT}$*;Pcdh17*$^{flox\text{-}EGFP/+}$ (Het) and *A2A-Cre;Rosa*$^{STOP\text{-}tdT}$*;Pcdh17*$^{flox\text{-}EGFP/flox}$ (cKO) mice. (**I**) Quantification of the EGFP intensities in the GPe. Line scan (left; dotted line indicates background intensity) and quantification of distances (right) are shown in the graphs. The axonal targeting of PCDH17-expressing iSPNs to the GPe is similar between Het and cKO mice (Distance 1; $P = 0.4191$, Distance 2; $P = 0.9172$, Distance 3; $P = 0.4499$). $n = 4$ mice per genotype. (**J**) EGFP intensity ratio between the anterior striatum and the inner GPe are similar between *A2A-Cre;Rosa*$^{STOP\text{-}tdT}$*;Pcdh17*$^{flox\text{-}EGFP/flox}$ mice (cKO) and *A2A-Cre;Rosa*$^{STOP\text{-}tdT}$*;Pcdh17*$^{flox\text{-}EGFP/+}$ mice (Het) ($P = 0.6225$). (**K**) Immunostaining for tdTomato (red) and EGFP (green: PCDH10-expressing iSPNs are labeled) of BG sections from adult *A2A-Cre;Rosa*$^{STOP\text{-}tdT}$*;Pcdh10*$^{flox\text{-}EGFP/+}$ (Het) and *A2A-Cre;Rosa*$^{STOP\text{-}tdT}$*;Pcdh10*$^{flox\text{-}EGFP/flox}$ (cKO) mice. (**L**) Quantification of the EGFP intensities in the GPe. The axonal targeting of PCDH10-expressing iSPNs to the GPe is similar between Het and cKO mice (Distance 1; $P = 0.4547$, Distance 2; $P = 0.9182$, Distance 3, $P = 0.3604$). $n = 5$ mice per genotype. (**M**) EGFP intensity ratio between the posterior striatum and the outer GPe are similar between *A2A-Cre;Rosa*$^{STOP\text{-}tdT}$*;Pcdh10*$^{flox\text{-}EGFP/flox}$ mice (cKO) and *A2A-Cre;Rosa*$^{STOP\text{-}tdT}$*;Pcdh10*$^{flox\text{-}EGFP/+}$ mice (Het) ($P = 0.7831$).
The scale bars represent 0.5 mm. Data are mean ± SEM. Student's *t*-test.

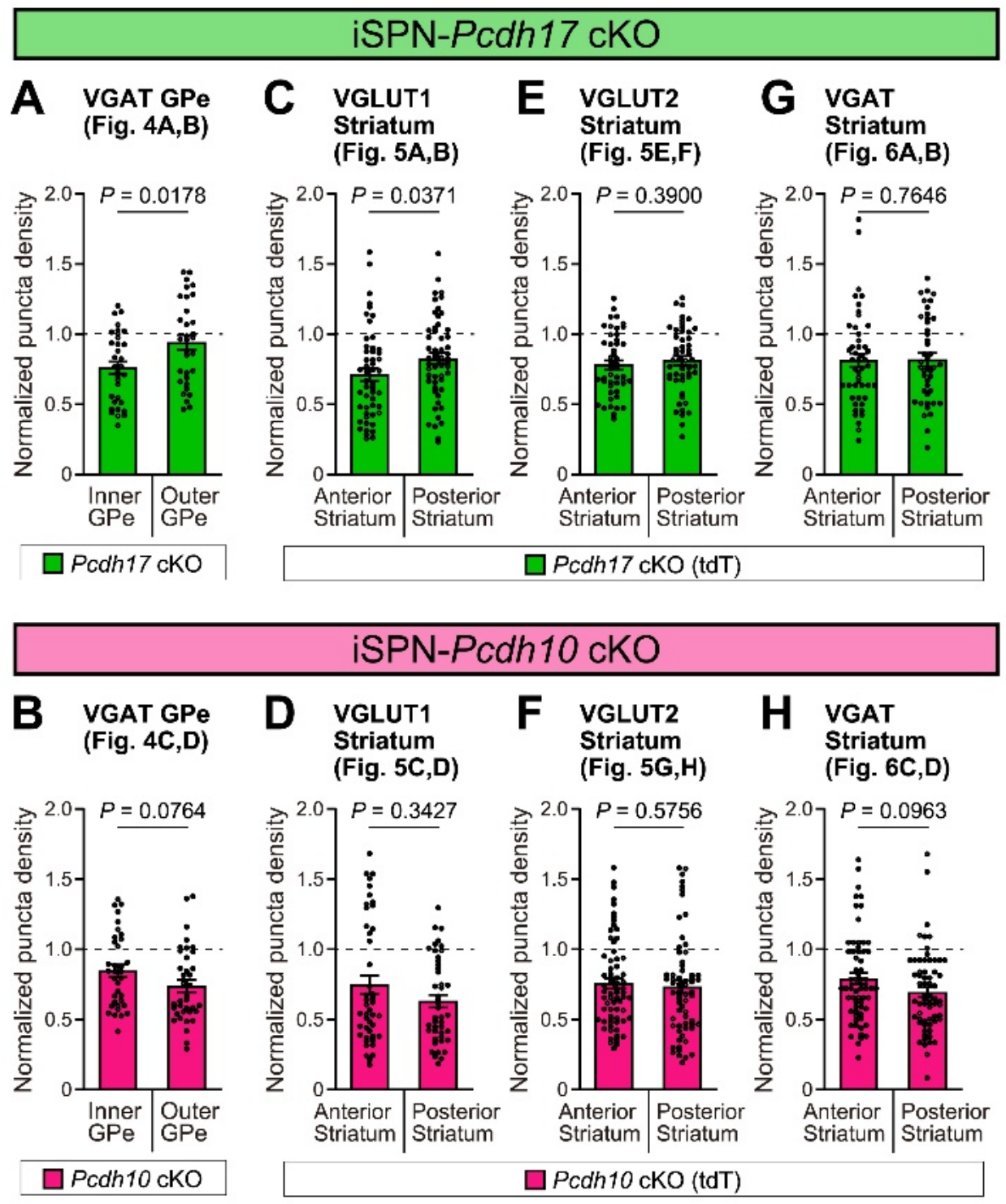

Fig. S11. Comparison of normalized synaptic puncta density between the inner and outer GPe, as well as between the anterior and posterior striatum, in iSPN-*Pcdh* cKO mice.
(**A** and **B**) Comparison of normalized VGAT puncta density between the inner and outer GPe in *Pcdh17* cKO and *Pcdh10* cKO mice from Fig. 4, A to D.
(**C** and **D**) Comparison of normalized VGLUT1 puncta density between the anterior and posterior striatum in *Pcdh10* cKO (tdT) and *Pcdh17* cKO (tdT) mice from Fig. 5, A to D.
(**E** and **F**) Comparison of normalized VGLUT2 puncta density between the anterior and posterior striatum in *Pcdh10* cKO (tdT) and *Pcdh17* cKO (tdT) mice from Fig. 5, E to H.
(**G** and **H**) Comparison of normalized VGAT puncta density between the anterior and posterior striatum in *Pcdh10* cKO (tdT) and *Pcdh17* cKO (tdT) mice from Fig. 6, A to D.
Data are mean ± SEM. Mann-Whitney U tests were performed between different regions in iSPN-*Pcdh* cKO mice, and the *P*-values are indicated.

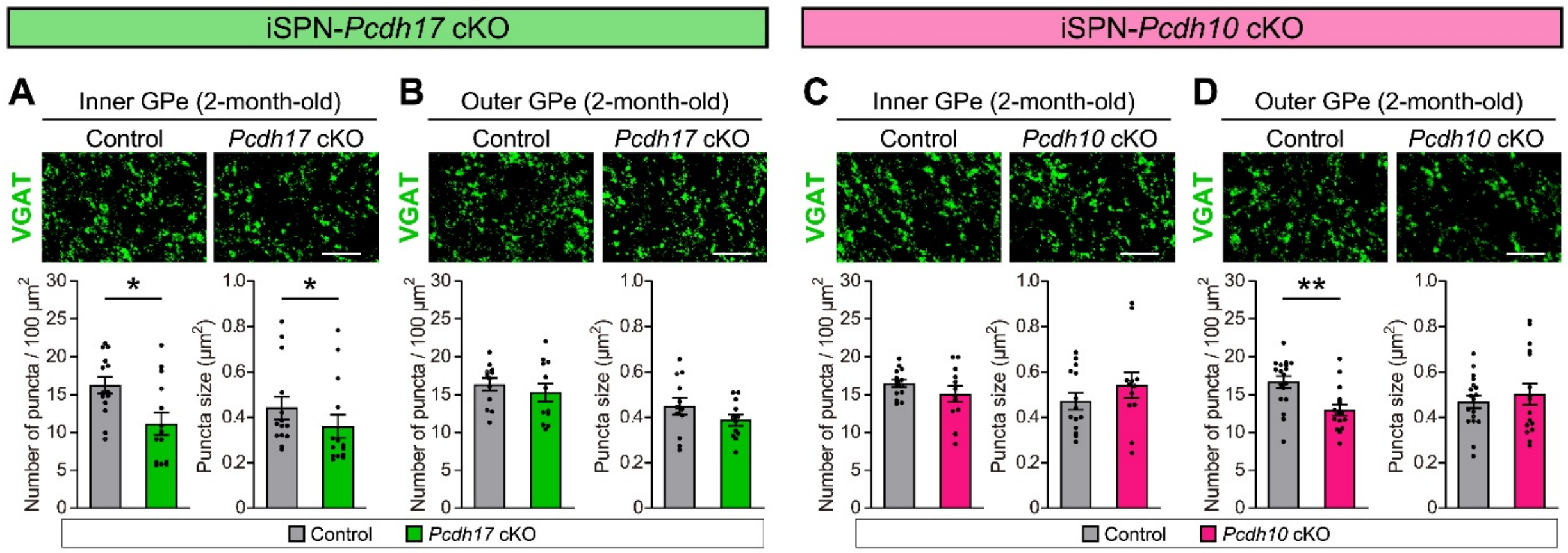

Fig. S12. Region-specific inhibitory synapse impairment in the GPe of adult *Pcdh* cKO mice.

(**A** to **D**) VGAT puncta in the inner GPe and outer GPe of 2-month-old mice. (**A** and **B**) In *Pcdh17* cKO mice, VGAT density is significantly decreased in the inner GPe (**A**; $P = 0.0141$), but not in the outer GPe (**B**; $P = 0.4776$). VGAT size is also significantly decreased in the inner GPe (**A**; $P = 0.0395$), but not in the outer GPe (**B**; $P = 0.1978$). (**C** and **D**) In *Pcdh10* cKO mice, VGAT density is significantly decreased in the outer GPe (**C**; $P = 0.0021$), but not in the inner GPe (**D**; $P = 0.4319$). VGAT size is not changed in the inner or outer GPe (**C**; $P = 0.4940$, **D**; $P = 0.9999$).

$n = 12$–16 fields from 3–4 mice/genotype. The scale bars represent 2 µm. *$P < 0.05$, **$P < 0.01$ by Mann-Whitney U test.

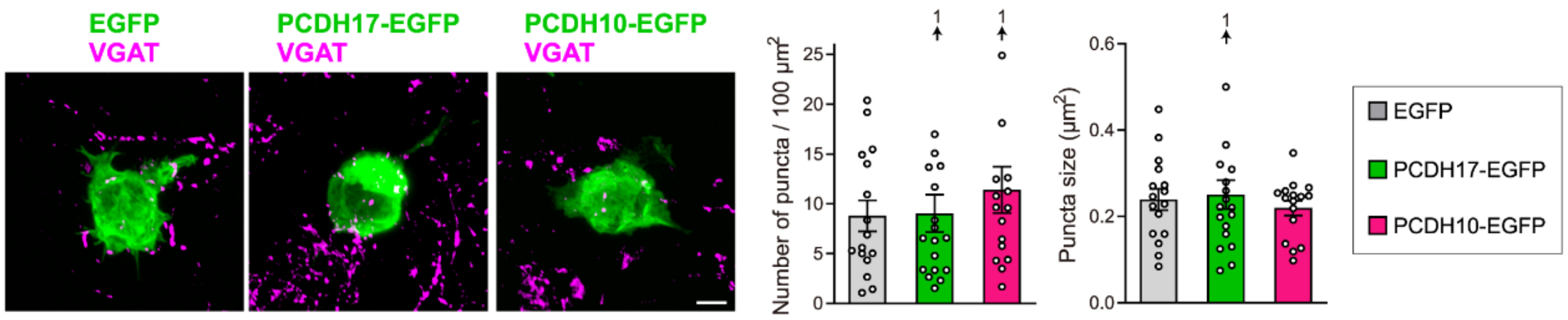

Fig. S13. PCDH17 and PCDH10 do not induce inhibitory presynaptic differentiation in a heterologous co-culture system.

HEK293T cells overexpressing EGFP, PCDH17-EGFP, or PCDH10-EGFP were cocultured with striatal neurons at DIV 10 and stained for VGAT (to label inhibitory presynaptic terminals) and GFP (to label HEK293T cells) at DIV 11. The density and size of VGAT puncta on the HEK293T cells were similar among the three conditions (density; $P = 0.9951$ for EGFP vs. PCDH17; $P = 0.5861$ for EGFP vs. PCDH10; size; $P = 0.9436$ for EGFP vs. PCDH17; $P = 0.8478$ for EGFP vs. PCDH10). Data were collected from n = 16 (EGFP), 18 (PCDH17-EGFP), and 16 (PCDH10-EGFP) cells from three independent experiments. One-way ANOVA followed by the Sidak test.

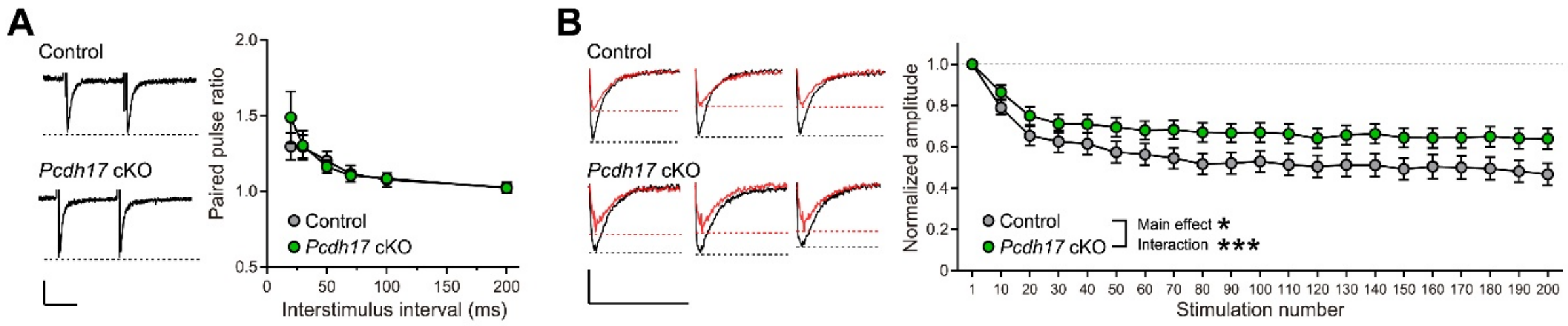

Fig. S14. Reduced synaptic depression at cortico-striatal synapses in *Pcdh17* cKO mice.
(A) Paired-pulse ratio (PPR) of evoked EPSCs at cortico-striatal synapses in the anterior striatum of P11–P23 *Pcdh17* cKO and control mice. (Left) Sample traces with a 20-ms interstimulus interval. (Right) PPR across a range of interstimulus intervals (20, 30, 50, 70, 100, and 200 ms). PPR is similar between *Pcdh17* cKO and control mice (main effect of genotype: $P = 0.5711$). $n =$ 35 neurons from 6 control mice; 39 neurons from 6 *Pcdh17 cKO* mice.
(B) Synaptic depression induced by prolonged repetitive stimulation (10 Hz, 200 stimuli) at cortico-striatal synapses in the anterior striatum of P11–P23 *Pcdh17* cKO and control mice. (Left) Sample traces showing responses 1–3 (black) and 198–200 (red). (Right) Quantification of normalized EPSC amplitudes (each point represents the average of 10 consecutive responses) reveals weaker synaptic depression in *Pcdh17* cKO mice compared to controls (main effect of genotype: $P = 0.0493$; genotype × stimulus interaction: $P = 0.0002$). $n = 39$ neurons from 6 control mice; 45 neurons from 6 *Pcdh17* cKO mice.
The scale bars represent 100 pA and 10 ms (**A** and **B)**. Data are mean ± SEM. $^{*}P < 0.05$, $^{***}P < 0.001$ by two-way ANOVA followed by the Sidak test (**A**) and two-way repeated-measures ANOVA followed by the Sidak test (**B**).

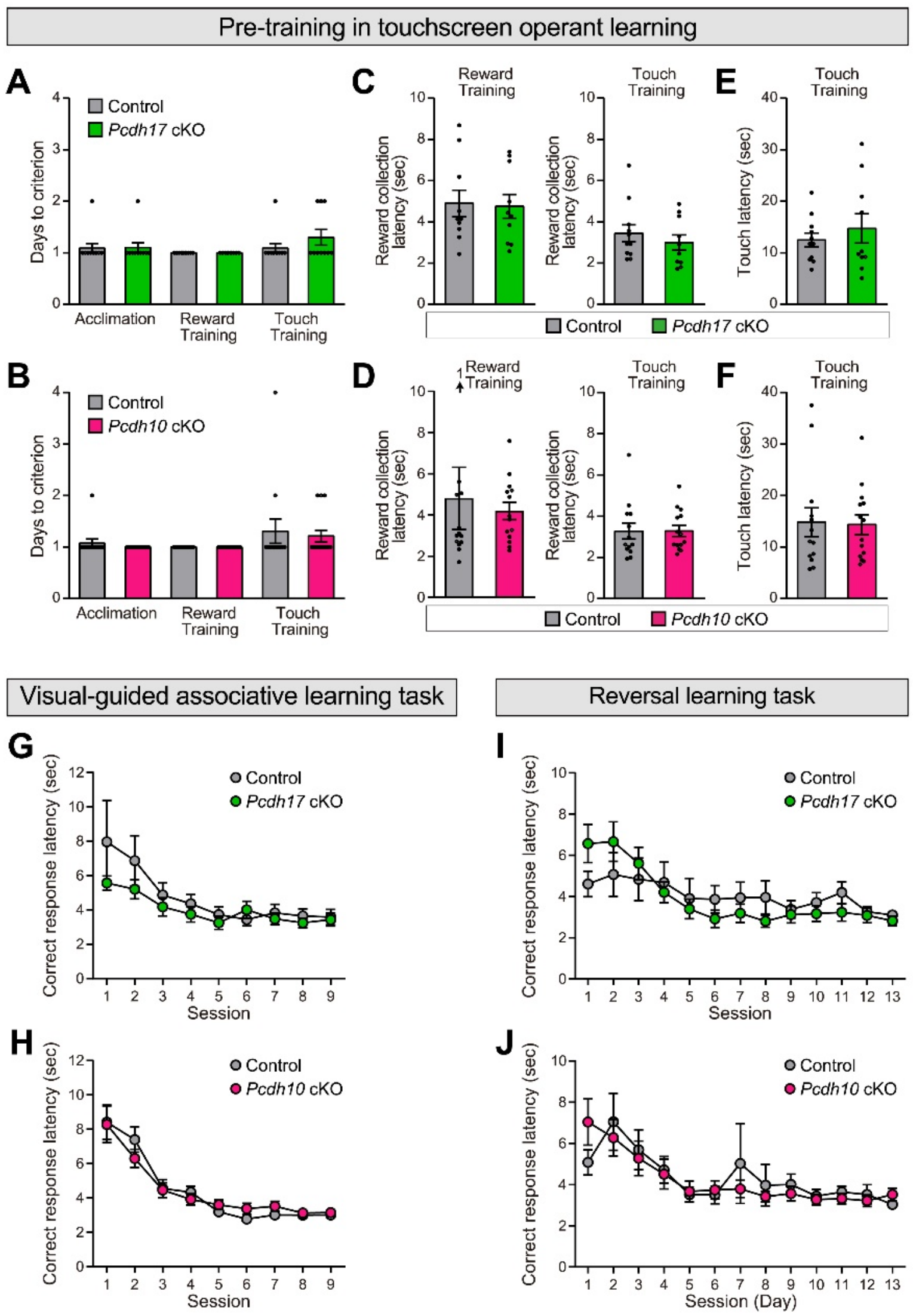

Fig. S15. Parameters during pre-training and correct response latencies during visual-guided associative learning and reversal learning tasks are normal in *Pcdh* cKO mice.
(**A** to **F**) Pre-training. (**A** and **B**) Days to criterion in acclimation (*Pcdh17*; *P* = 0.9469, *Pcdh10*; *P* = 0.3086), reward acquisition training, and stimulus touch training (*Pcdh17*; *P* = 0.2439, *Pcdh10*; *P* = 0.7193) are similar between *Pcdh17* cKO mice and control mice (**A**) and between *Pcdh10* cKO mice and control mice (**B**). (**C** and **D**) Reward latencies during reward training (*Pcdh17*; *P* = 0.8721, *Pcdh10*; *P* = 0.6855) and touch training (*Pcdh17*; *P* = 0.4352, *Pcdh10*; *P* = 0.9851) are similar between *Pcdh* cKO mice and control mice. (**E** and **F**) Touch latencies during touch training (*Pcdh17*; *P* = 0.4747, *Pcdh10*; *P* = 0.8894) are similar between *Pcdh* cKO mice and control mice. n = 11 mice for *Pcdh17* control; 10 for *Pcdh17* cKO; 13 for *Pcdh10* control; and 14 for *Pcdh10* cKO.
(**G** and **H**) Correct response latencies during the visual-guided associative learning task are similar between *Pcdh17* cKO mice and *Pcdh17* control mice (**G**) and between *Pcdh10* cKO mice and *Pcdh10* control mice (**H**). *n* = 10 mice for *Pcdh17 control*; 10 for *Pcdh17 cKO;* 13 for *Pcdh10 control*; and 14 for *Pcdh10 cKO*.

(**I** and **J**) Correct response latencies during the reversal learning task are similar between *Pcdh17* cKO mice and control mice (**I**) and between *Pcdh10* cKO mice and control mice (**J**). *n* = 10 mice for *Pcdh17 control*; 10 for *Pcdh17 cKO;* 13 for *Pcdh10 control*; and 14 for *Pcdh10 cKO*. 2- to 4-month-old male mice were used. Data are mean ± SEM. Student's *t*-test.

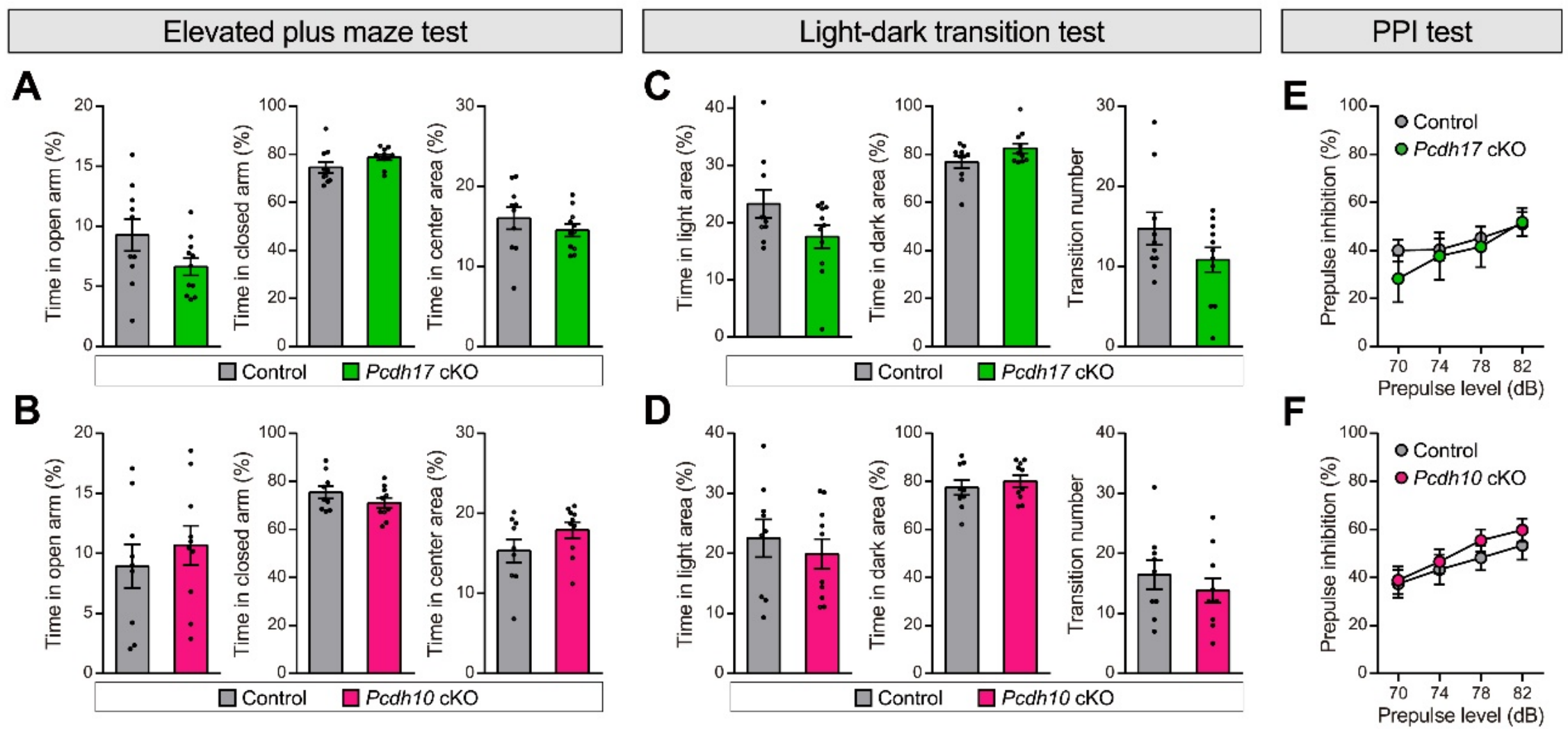

Fig. S16. Normal anxiety levels and sensorimotor gating in *Pcdh* cKO mice.
(**A** and **B**) Elevated plus maze test. There is no significant difference between *Pcdh* cKO and control mice in the time spent in the open arms (*Pcdh17*; *P* = 0.0879, *Pcdh10*; *P* = 0.4779), closed arms (*Pcdh17*; *P* = 0.1137, *Pcdh10*; *P* = 0.1987), or center area (*Pcdh17*; *P* = 0.3435, *Pcdh10*; *P* = 0.1531). *n* = 10 mice for *Pcdh17 control*; 11 for *Pcdh17 cKO;* 9 for *Pcdh10 control*; and 10 for *Pcdh10 cKO*.
(**C** and **D**) Light-dark transition test. There is no significant difference between *Pcdh* cKO mice and control mice in the time spent in the light area (*Pcdh17*; *P* = 0.0866, *Pcdh10*; *P* = 0.5182), dark area (*Pcdh17*; *P* = 0.0866, *Pcdh10*; *P* = 0.5182), or transition numbers (*Pcdh17*; *P* = 0.1405, *Pcdh10*; *P* = 0.4170). *n* = 10 mice for *Pcdh17 control*; 11 for *Pcdh17 cKO;* 9 for *Pcdh10 control*; and 10 for *Pcdh10 cKO*.
(**E** and **F**) PPI test. PPI (%) at different prepulse levels is similar between *Pcdh17* control and *Pcdh17* cKO mice (**E**) as well as between *Pcdh10 control* and *Pcdh10 cKO* mice (**F**). *n* = 11 mice for *Pcdh17 control*; 9 for *Pcdh17 cKO;* 11 for *Pcdh10 control*; and 11 for *Pcdh10 cKO*. 2- to 4-month-old male mice were used. Data are mean ± SEM. Student's *t*-test.
